# Neurophysiological alterations during sensory processing in autism - a meta-analysis

**DOI:** 10.1007/s00787-025-02917-0

**Published:** 2025-11-22

**Authors:** Anjuli Ghosh, Natalia Nasarre-Nacenta, Sarah Baumeister, Nathalie E. Holz, Tobias Banaschewski, Daniel Brandeis, Pascal-M. Aggensteiner, Anna Kaiser

**Affiliations:** 1 Department of Child and Adolescent Psychiatry and Psychotherapy, Medical Faculty Mannheim, Central Institute of Mental Health, Heidelberg University, Mannheim, J 5 · 68159 Germany; 2 German Center for Mental Health (DZPG), partner site Mannheim-Heidelberg-Ulm, Heidelberg, Germany; 3 School of Health and Social Sciences, AKAD University, Stuttgart, Germany; 4 Department of Child and Adolescent Psychiatry and Psychotherapy, University Hospital of Psychiatry, University of Zurich, Zurich, Switzerland; 5 Department of Child and Adolescent Psychiatry/Psychotherapy, University Hospital of Ulm, University of Ulm, Ulm, Germany; 6 Department of Clinical Psychology and Psychotherapy of Childhood and Adolescence, Institute of Psychology, University of Freiburg, Freiburg, Germany

**Keywords:** Autism, Sensory processing, Electroencephalography, Magnetoencephalography, Neurophysiology, Meta-analysis

## Abstract

While sensory processing alterations in autism are well-documented, the neurophysiological correlates remain unclear. This meta-analysis examined differences in early event-related potentials (ERP) and event-related fields (ERF) between autistic and non-autistic individuals using electroencephalography and magnetoencephalography to identify neurophysiological alterations that may underlie variations in sensory perception, communication, and social interaction in autism. Following PRISMA guidelines, a database search was conducted for peer-reviewed studies published from January 1980 onwards. Random-effects meta-analyses were performed using the metafor package in R. Standardised mean-group differences in early ERP/ERF latencies and amplitudes were analysed with moderator analyses exploring demographic and methodological factors, including neurophysiological technique, sensory modality, age group, sex, and language impairment. 145 studies (3778 autistic, 3484 non-autistic participants) were included. Autistic individuals exhibited significantly longer latencies in P/M50 (SMD = 0.44; SE = 0.21; 95% CI 0.03–0.86; *p* = 0.04), P/M100 (SMD = 0.18; SE = 0.08; 95% CI 0.01–0.36; *p* = 0.03), N170 (SMD = 0.33; SE = 0.12; 95% CI 0.10–0.56; *p* = 0.01), and P/M200 (SMD = 0.30; SE = 0.09; 95% CI 0.12–0.48; *p* = 0.00) components. P/M50 showed the greatest latency alteration, with an effect-size nearing medium, especially in individuals with language impairment (Q_B_(2) = 7.70, *p* = 0.02), followed by N170 most notable in autistic adolescents and adults (Q_B_ (3) = 12.30, *p* = 0.01). No significant amplitude alterations were found, and substantial heterogeneity was observed. Neurophysiological characteristics of sensory processing in autism implicate multiple mechanisms and stages given prolonged P/M50- and N170-latency (associated with sensory filtering challenges and social perception alterations, respectively). These component timings show potential as biomarkers, though heterogeneity and modest effect-sizes limit clinical application, highlighting the need for further research.

**Supplementary Information:**

The online version contains supplementary material available at 10.1007/s00787-025-02917-0.

## Introduction

### Sensory alterations in autism and their functional significance

Autism is a lifelong neurodevelopmental condition characterised by alterations in verbal and nonverbal communication, social interaction, patterns of repetitive behaviour and specialised interests [1]. Affecting approximately 1% of the global population [2], autism is among the most prevalent neurodevelopmental conditions worldwide. Sensory alterations are highly prevalent in autism, affecting up to 90% of autistic individuals [3–5]. Also formally recognised as a diagnostic feature of autism in the Diagnostic and Statistical Manual of Mental Disorders, 5th edition (DSM-5) [6] and its Text Revision (DSM-5-TR) [1], as well as the International Classification of Diseases, 11th edition (ICD-11) [7], sensory alterations have been explored as potential (bio-)markers for autism [8, 9]. These alterations can manifest in a variety of ways, including hypersensitivity, avoidance of sensory stimuli, diminished responses to sensory stimulation, and/or sensory seeking behaviour [10, 11]. Hypersensitivity or an intense attraction to specific stimuli, for example, may trigger strong reactions to lights, sounds, physical contact, and odours [12], while hyposensitivity traits, such as walking into objects or not noticing temperature changes are also quite common in autism [13–15]. Although sensory alterations can lead to positive effects, such as improved focus, they often result in negative experiences, causing distraction, anxiety, and limiting daily activities [16]. Research on sensory processing in autism focuses on auditory, tactile, and visual domains. Delayed auditory responses may contribute to language impairment in autistic children, linking slower processing to communication difficulties [17]. In visual processing, a focus on local details may enhance specific strengths but also contribute to difficulties with complex social cues [18]. In tactile processing, altered neural responses to touch may affect (non-)comfort with physical contact and the emotional evaluation of touch [19].

Sensory-based support, such as sensory integration therapy, may reduce distress behaviours and enhance daily life in autistic people, but its long-term benefits remains uncertain, necessitating further research [20–23].

### EEG and MEG: exploring brain dynamics through ERPs and ERFs

Several studies have explored neurophysiological mechanisms associated with sensory processing in autism using electroencephalography (EEG) and magnetoencephalography (MEG). These methods record early event-related potentials (ERPs) and magnetic fields (ERFs). EEG and MEG offer high temporal resolution, essential for examining sensory responses, which occur within the first few hundred milliseconds after stimulus presentation [24]. Both capture overlapping information [25, 26], but EEG is more widely accessible and cost-effective [27], while MEG provides higher spatial resolution for some cortical sources and is contactless [28, 29], benefiting autistic individuals who often exhibit tactile sensitivities [30].

Systematic reviews and meta-analyses, focusing on selected early ERP/ERF components, either targeted directly as primary outcomes or examined within the context of specific paradigms, have often reported latency differences between autistic and non-autistic individuals. Prolonged latencies have for example been documented for M50, P100 and N170 [31, 32] findings that show altered early sensory-processing pathways in autism. Notably, the N170 has been the most prominently identified candidate physiological biomarker for autism [9]; delayed N170 latency has been linked specifically to face-processing and social-communication alterations in autism in multiple studies. Some systematic reviews and meta-analyses also reported reduced component amplitudes in autism. Examples include attenuated mismatch negativity (MMN) amplitude [33, 34] and reduced N200 amplitude [31]. Reductions in P200 amplitude have also been proposed as a potential biomarker for sensory and language-related traits in autism [35]. However, these reviews have remained restricted in scope, and an exhaustive meta-analysis covering all relevant components is still lacking.

A detailed overview of the early ERP/ERF components examined in the current analysis, along with their associated neuropsychological correlates reflecting sensory processing is provided in Table 1.

**Table 1:** Overview of ERP/ERF components and their sensory processing correlates

ERP/ERF component	Sensory processing correlates
P/M50	Preattentive arousal, sensory gating [36, 37]
P/M100	Spatial attention, sensory gating [38, 39]
N100	Orienting response to an unexpected stimulus, matching response with previous stimuli, attentional discrimination [37, 40]
N170	Perception of face processing [36, 41]
P/M200	Attention selection, sensation-seeking [37, 42, 43]
N200	Detection of stimulus change; automatic encoding (N2a/MMN); task-relevant changes (N2b); stimulus classification (N2c) [37]
MMN/MMF	Automatic brain response to stimulus difference or change [37, 44]

### Objectives

Despite several broader systematic reviews [8, 45], to our knowledge, no meta-analysis has yet comprehensively and systematically synthesised all early ERP/ERF components related to sensory processing in autism by quantitatively combining results from relevant studies. Therefore, this meta-analysis focuses on studies utilising EEG and MEG to investigate sensory processing, providing a clearer understanding of the neurophysiological correlates of sensory alterations in autism.

It summarises relevant literature on early ERP/ERF alterations in autistic children, adolescents, and adults compared to non-autistic individuals. The focus was to identify neurophysiological group-level differences in sensory processing (auditory, visual, tactile, olfactory, gustatory) between these two groups. We hypothesized smaller ERP/ERF amplitudes and longer ERP/ERF latencies in autistic individuals compared to non-autistic individuals reflecting alterations in sensory processing in the former group. Furthermore, the current work aimed at addressing heterogeneity by defining (based on previous studies) and analysing potentially relevant demographic (age groups, language impairment, co-occurring condition, medication, sex, IQ), methodological (MEG/EEG, modality of stimulus presentation, task type, for the N170 component additionally condition type) and further study-related (diagnostic criteria, year of publication) moderators. A further aim was to identify gaps in research to be addressed in future studies.

## Methods

### Search strategy

The current meta-analysis was registered on PROSPERO (https://www.crd.york.ac.uk/prospero/display_record.php?RecordID=370924).

The literature search was conducted according to the PRISMA 2020 guidelines [46]. An initial search was performed using the databases MEDLINE (via PubMed), PsycINFO, Web of Science, and the Clinical Trials Register on 22nd and 23rd of November 2022. The following key words were used: autism, EEG or MEG and sensory processing (the specific search strings are presented in sMethods in the online resource). An update of the literature search was conducted on 23rd of April 2024. Additionally, studies from the reference list of five reviews [8, 31, 34, 47, 48] were included. The initial screening was conducted blind and independently by the first and second author, beginning with title and abstract screening followed by full-text screening. Both reviewers worked independently before comparing their decisions, and disagreements (fewer than 5%) were resolved through discussion with the senior authors of this project. Microsoft Excel was used to support the screening process and to detect duplicates. Authors were contacted to inquire about unpublished findings, clarify graphical data, or resolve uncertainties in result interpretation. This was done to ensure we had access to the most comprehensive and accurate information, minimising the risk of missing key results and reducing potential publication bias in our analysis. In total, 139 authors were approached, of whom around 40 (29%) responded, with 18 (13%) ultimately providing data that were incorporated into the analysis.

**Fig. 1:**
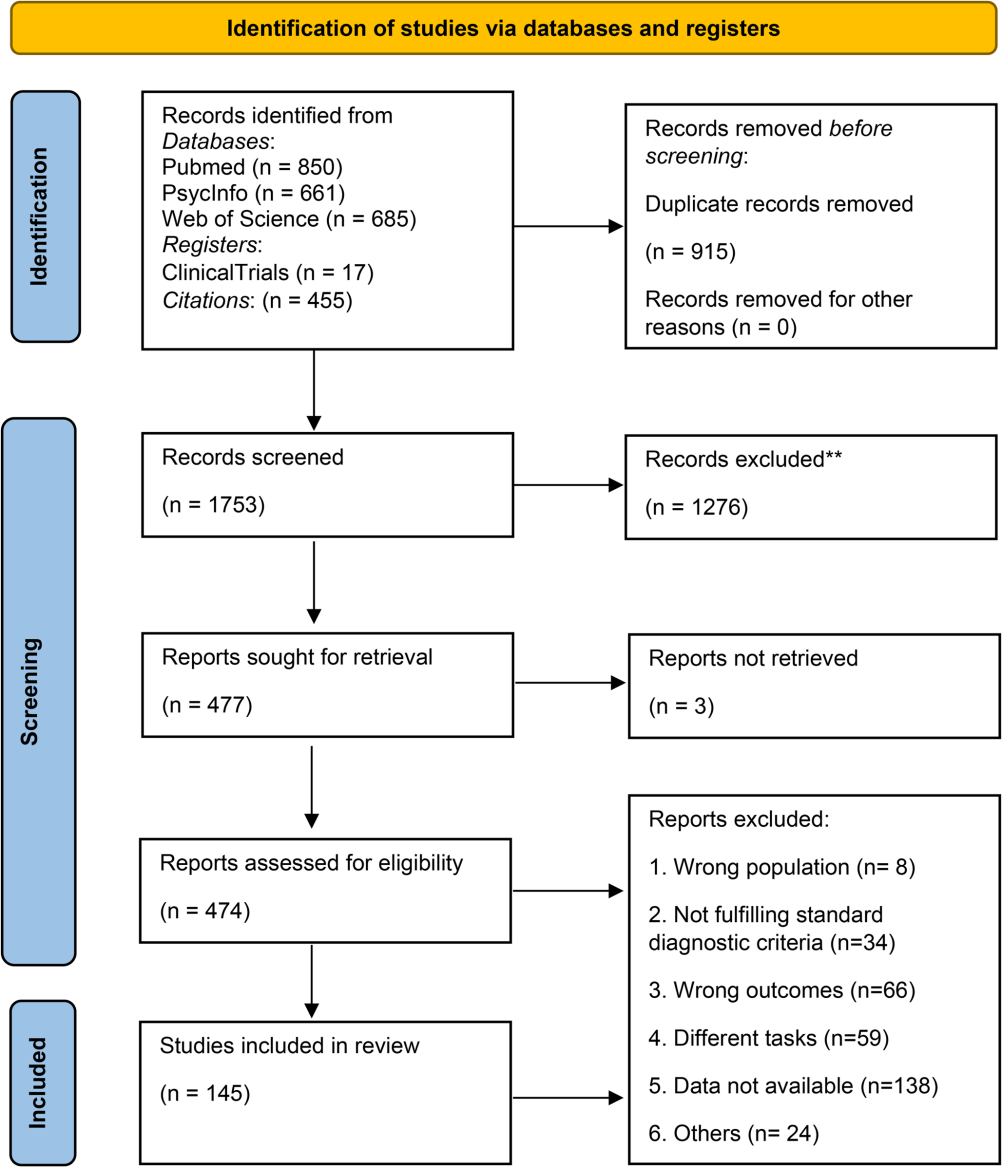
Flowchart of the literature selection process according to the PRISMA guidelines [46]

An initial pool of 1753 references was obtained. Figure 1 provides an overview of the screening process and record counts.

### Eligibility and selection

Studies were included if they were:

- Recording electroencephalography (EEG) or magnetoencephalography (MEG) while participants engaged in tasks about auditory, visual, tactile, olfactory or gustatory perception.
- Reporting quantitative data to compare neurophysiological indices of sensory processing between autistic and non-autistic children, adolescents and adults.
- The autistic group having a standard clinical diagnosis of autism according to: DSM-III, DSM-III-R, DSM-IV, DSM-IV-TR, DSM-V, ICD-10 or ICD-11 or with above cut-off scores on validated rating scales ADOS or ADI-R.
- Published in one of the following languages: English, German, French or Spanish.
- Journal articles published in peer-reviewed journals.
- Published between January 1980 (DSM-III releasing year) and April 2024.
- Sufficient information to calculate the effect size.

### Data coding and extraction

The coding and part of the data extraction were performed using the Covidence software [49]. All relevant variables were collected in a coding sheet (sMethods in the online resource). The first and second author collected, extracted, and coded the data from the studies independently and consulted each other during the process. Disagreements (< 5%) were solved in discussion with the senior authors.

### Quality assessment

A combination of the modified Newcastle-Ottawa Scale (NOS) assessing the quality of nonrandomized studies in meta-analyses [50] and a self-constructed scale evaluating the quality of EEG/MEG recordings was used (sMethods in the online resource).

### Data synthesis

Data analysis and visualisation were conducted via the *metafor* package [51]; (version 4.4.0) in R Studio (version 2023.09.1 + 494) [52]. Standardised mean differences (SMD) in ERP/ERF amplitudes and latencies between autistic and non-autistic individuals were used as effect-size measure [53]. Only studies reporting exact numerical values, specifically means with standard deviations or standard errors (which were converted to standard deviations), were extracted; studies with graphical-only data were included only if the necessary values could be obtained from the authors.

For positive ERP/ERF amplitudes, negative SMD indicated smaller amplitudes in the autistic group compared to the non-autistic group. Conversely, for negative ERP/ERF amplitudes, positive SMD indicated smaller amplitudes in the autistic group. For ERP/ERF latencies, positive SMD reflected longer latencies in the autistic group compared to the non-autistic group (and vice versa). Effect-sizes were calculated using exact statistics from the publications or obtained directly from authors. A mean effect-size was then computed for each ERP/ERF component to assess alterations in amplitude and latency. First multilevel models using random-effects assumptions were fitted to estimate mean effect-sizes, allowing for inferences beyond specific studies [54]. These models address data dependencies from multiple effect-sizes within the same study (e.g. different electrode localizations, different stimuli) [51] and ensure that studies reporting several contrasts do not exert disproportionate influence. Studies were weighted by heteroscedastic sampling variance, and mixed-effects models were used to explore moderator effects. This included demographic (age groups, language impairment, co-occurring condition, medication, sex, IQ), methodological (MEG/EEG, modality of stimulus presentation, task type) and further study-related (diagnostic criteria, year of publication) moderators. All moderators, except IQ and sex were categorical. For sex, we used the proportion of males in the autistic group. For IQ, we extracted the mean and standard deviation values from standardized intelligence tests and used the group mean IQ of the autistic sample as a continuous moderator. Heterogeneity was assessed using Cochrane’s Q-test [53], with Q_W_ indicating residual heterogeneity and Q_B_ testing moderator impact. The REML-estimator measured heterogeneity in effect-sizes. In addition, a study-level effect-size model averaging all contrasts within each study using inverse-variance weights was computed. Importantly, both approaches yielded identical results in terms of mean effect-size estimates, confidence intervals, and significance. This approach was only used to check robustness and to simplify presentation of the forest plots.

Other sensitivity analyses checked robustness and trim-and-fill analyses explored potential publication bias by identifying and adjusting for asymmetry in the funnel plot.

## Results

### Study characteristics

7262 participants across 145 studies were included (19 studies were added during the updated search). 119 studies used EEG and 26 studies MEG. 83 studied children, 24 adolescents, and 38 adults. 20.62% of the participants were female. Characteristics of the included studies can be found in sTable e1 in the online resource. A summary of demographic characteristics across all ERP/ERF components is presented in Table 2. The demographic characteristics displayed separately for each component are represented in sTables 2, 3, 4, 5, 6, 7, 8, 9, 10 and 11, 12, 13, 14 and 15 in the online resource.

**Table 2:** Demographic information across all ERP/ERF components

	Autistic	Non-autistic			
Total *n*	3778	3484	t	df	*p*
Age (years). M (SD)	12.86 (6.92)	13.54 (7.37)	3.99	7028	< 0.0001
Male (%). M (SD)	84.25 (11.52)	74.23 (17.17)	27.06	6172	< 0.0001
IQ. M (SD)	95.70 (16.01)	110.08 (8.55)	30.47	3198	< 0.0001
Children *n* (< 12.0y)	2333	2110	t	df	p
Age (years). M (SD)	8.76 (2.57)	9.03 (2.45)	3.58	4341	0.0003
Male (%). M (SD)	86.12 (8.56)	75.25 (15.77)	26.68	3802	< 0.0001
IQ. M (SD)	91.80 (16.66)	108.83 (9.37)	28.63	2187	< 0.0001
Adolescent *n* (12.0–<18.0y)	737	633	t	df	p
Age (years). M (SD)	13.95 (1.57)	14.33 (1.61)	4.30	1298	< 0.0001
Male (%). M (SD)	83.39 (5.09)	70.15 (16.92)	18.72	1176	< 0.0001
IQ. M (SD)	96.45 (8.52)	112.24 (4.23)	13.64	223	< 0.0001
Adults *n* (≥ 18.0y)	708	741	t	df	p
Age (years). M (SD)	25.31 (5.02)	25.91 (5.03)	2.22	1385	0.03
Male (%). M (SD)	78.70 (19.29)	74.84 (20.47)	3.34	1190	0.0009
IQ. M (SD)	106.41 (10.52)	113.15 (5.22)	11.07	784	< 0.0001

### Meta-analyses on amplitudes

No significant group differences emerged for the amplitude analyses. The overall mean estimated SMD from the multilevel models, along with the corresponding heterogeneity estimates, are shown in Table 3.

**Table 3:** Overall mean estimated true effect-sizes from random-effects models/multilevel linear models on amplitudes

ERP/ERF component	k^a^	SMD	95% CI	Q_W_^b^ (df, *p*)
P/M50	39	−0.09	[−0.41,0.23]	169.66 (38, < 0.0001)
P/M100	232	−0.15	[−0.31,0.01]	643.67 (231, < 0.0001)
N100	93	−0.07	[−0.19,0.05]	162.94 (92, < 0.0001)
N170	154	0.05	[−0.15,0.24]	461.55 (153, < 0.0001)
P/M200	64	−0.05	[−0.20,0.11]	142.13 (63, < 0.0001)
N200	67	0.20	[−0.01,0.41]	240.45 (66, < 0.0001)
MMN/MMF	211	0.04	[−0.09,0.16]	472.93 (210, < 0.0001)

^*a*^ k = number of included effect sizes. ^*b*^*Qw =* Cochran’s Q statistic for heterogeneity test. *** *p* < 0.001, ** *p* < 0.01, * *p* < 0.05

### Meta-analyses on latencies

A significant positive mean estimated SMD in the latencies of four ERP/ERF components: P/M50 (SMD = 0.44 [0.03, 0.86], *p* = 0.04), P/M100 (SMD = 0.18 [0.01, 0.36], *p* = 0.03), N170 (SMD = 0.33 [0.10, 0.56], *p* = 0.006), and P/M200 (SMD = 0.30 [0.12, 0.48], *p* = 0.001) emerged, indicating longer latencies in autistic compared to non-autistic individuals. Group differences in latencies for other ERP/ERF components remained insignificant (all p values > 0.05) or lacked sufficient studies for comparison (k ≤ 2). The overall mean estimated SMD from the multilevel models, along with the corresponding heterogeneity estimates, are shown in Table 4.

**Table 4:** Overall mean estimated true effect-sizes from random-effects models/multilevel linear models on latencies

ERP/ERF component	k^a^	SMD	95% CI	Q_W_^b^ (df, *p*)
P/M50	47	0.44*	[0.03,0.86]	195.92 (46, < 0.0001)
P/M100	187	0.18*	[0.01,0.35]	526.15 (186, < 0.0001)
N100	67	0.16	[−0.09,0.41]	202.03 (66, < 0.0001)
N170	119	0.33**	[0.10,0.56]	391.11 (118, < 0.0001)
P/M200	41	0.30**	[0.12,0.48]	56.95 (40, 0.04)
N200	62	0.18	[−0.19,0.54]	240.12 (61, < 0.0001)
MMN/MMF	156	0.27	[−0.19,0.72]	687.64 (155, < 0.0001)

^*a*^ k = number of included effect sizes. ^*b*^*Qw =* Cochran’s Q statistic for heterogeneity test. *** *p* < 0.001, ** *p* < 0.01, * *p* < 0.05

**Fig. 2:**
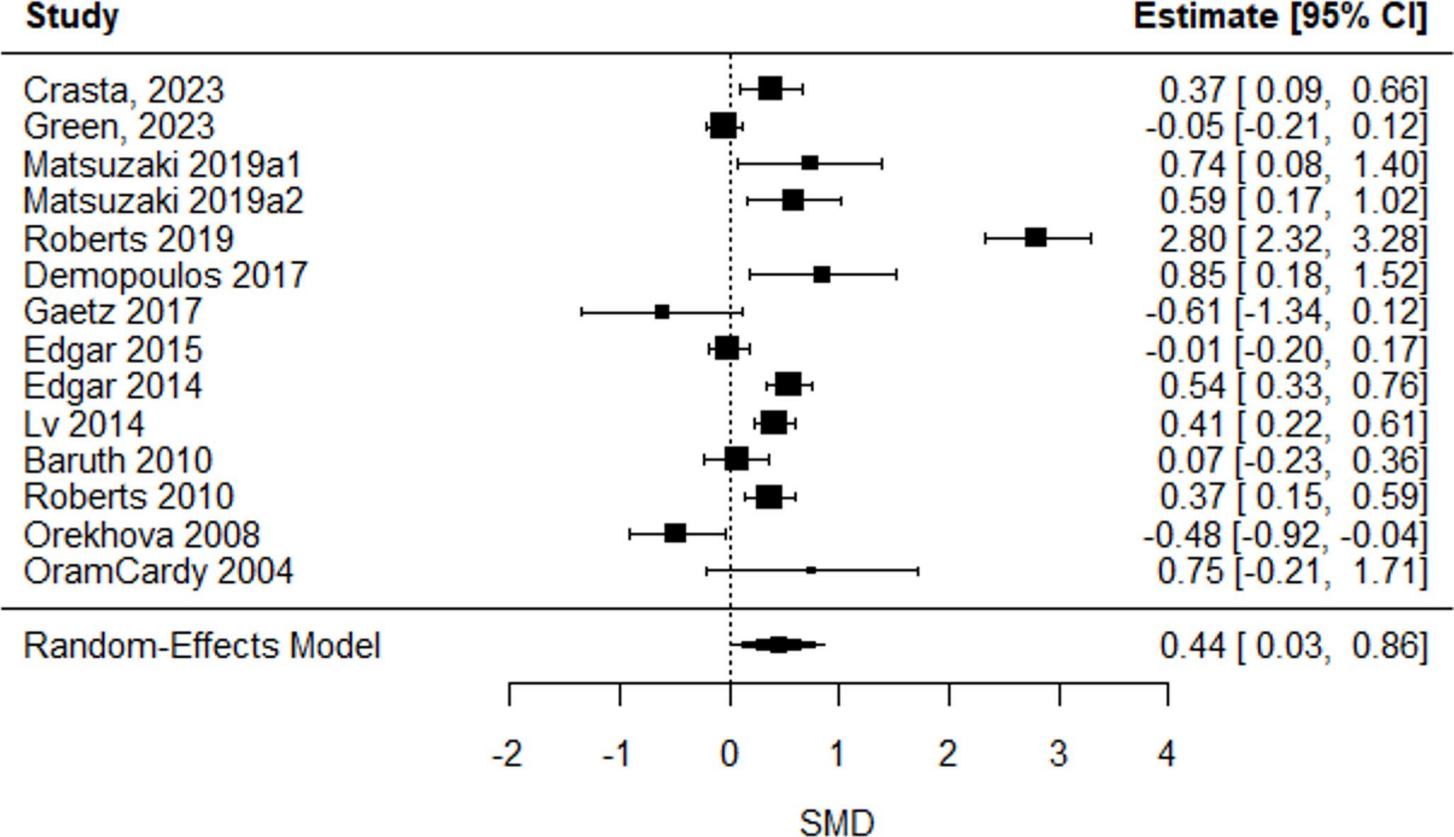
P/M50-latency forest plot. Positive SMD reflected longer latencies in the autistic group compared to the non-autistic group. One effect size per study, obtained by averaging multiple outcomes within each study (see forest plot from multilevel analysis in sFig. 2 in the online resource)

**Fig. 3:**
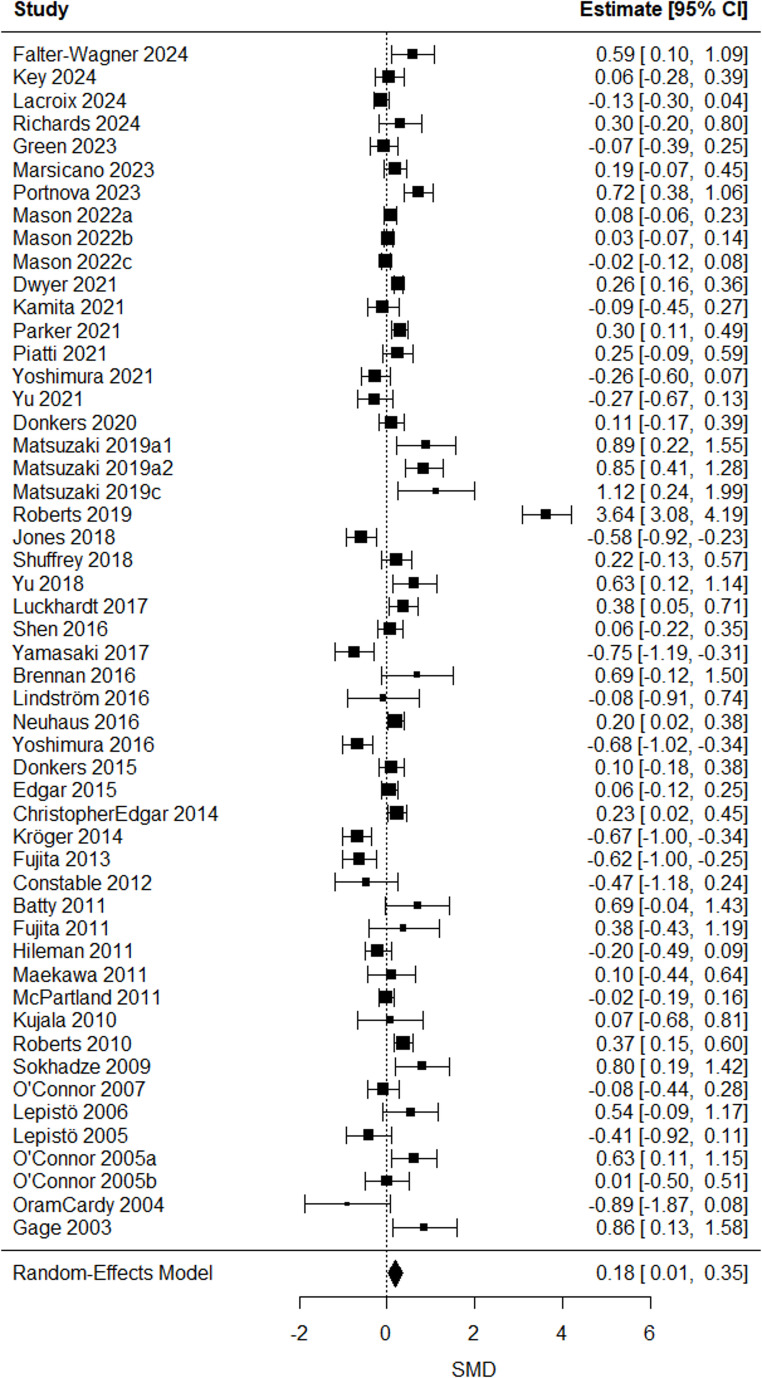
P/M100-latency forest plot. Positive SMD reflected longer latencies in the autistic group compared to the non-autistic group. One effect size per study, obtained by averaging multiple outcomes within each study (see forest plot from multilevel analysis in sFig. 4 in the online resource)

**Fig. 4:**
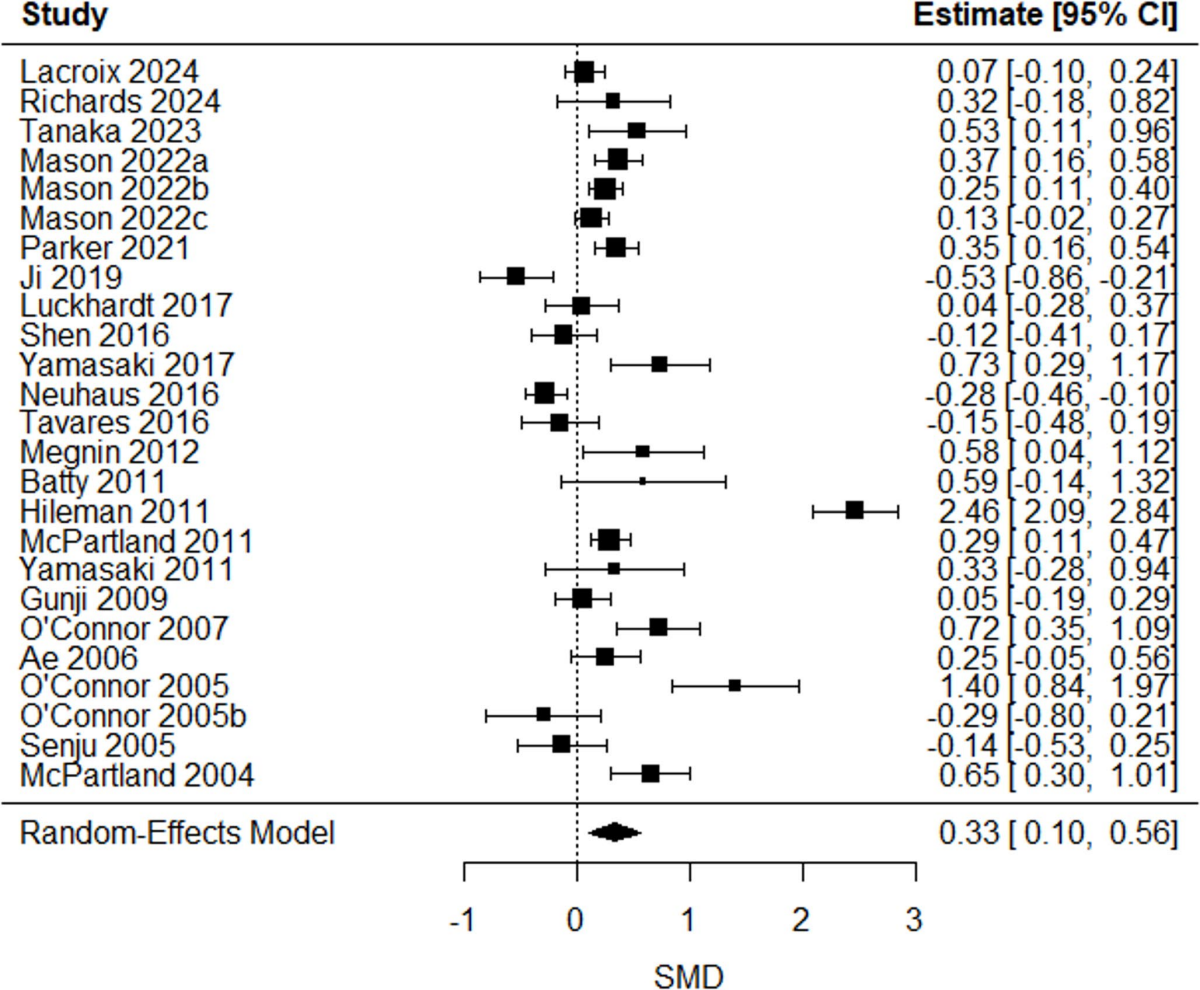
N170-latency forest plot. Positive SMD reflected longer latencies in the autistic group compared to the non-autistic group. One effect size per study, obtained by averaging multiple outcomes within each study (see forest plot from multilevel analysis in sFig. 10 in the online resource)

**Fig. 5:**
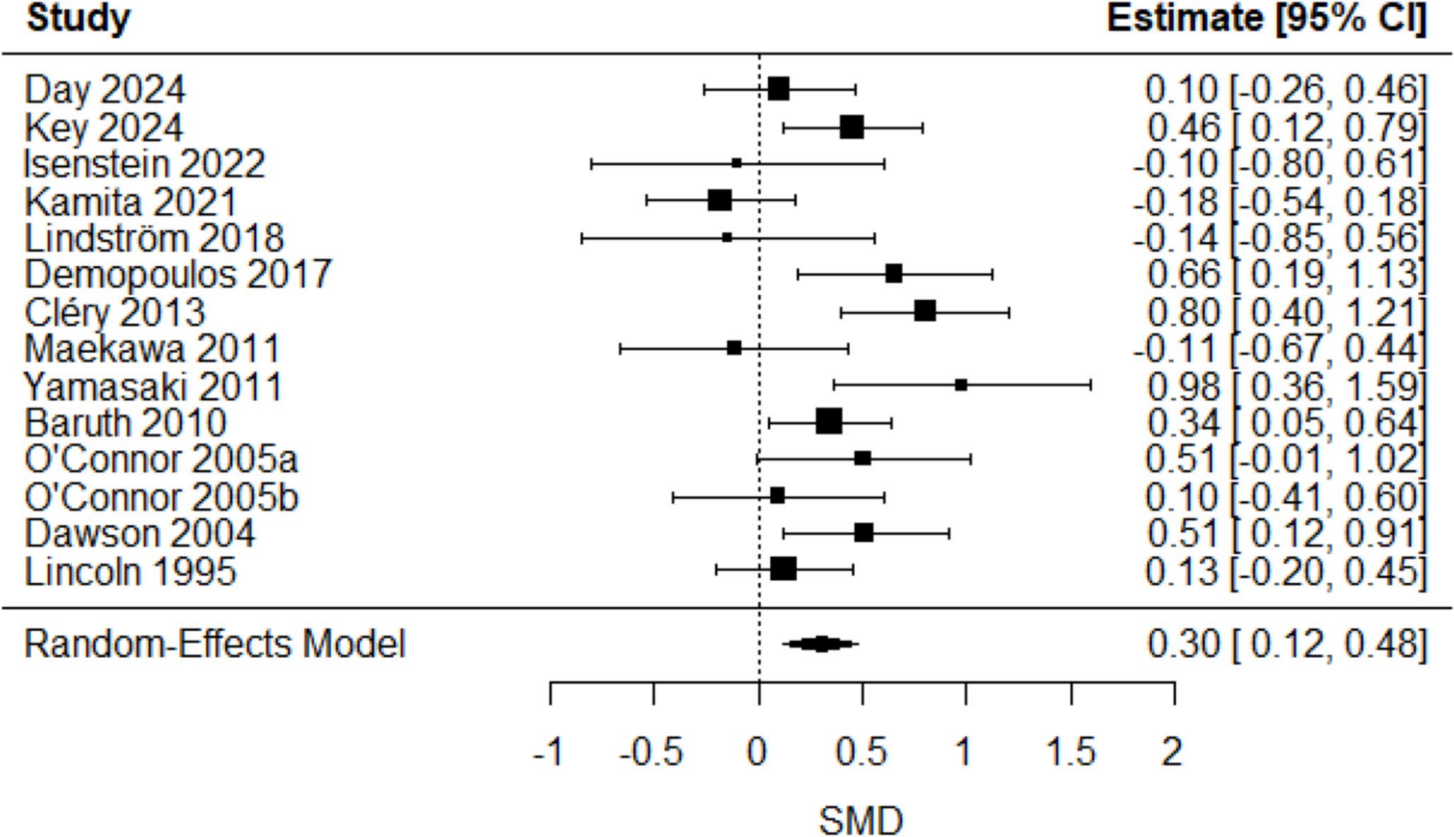
P/M200-latency forest plot. Positive SMD reflected longer latencies in the autistic group compared to the non-autistic group. One effect size per study, obtained by averaging multiple outcomes within each study (see forest plot from multilevel analysis in sFig. 6 in the online resource)

Simplified forest plots for P/M50, P/M100, N170 and P/M200 latencies are shown in Figs. 2, 3, 4, and 5. Forest plots for amplitudes and all other latencies derived from the multilevel model are provided in the online resource sFigs. 1, 2, 3, 4, 5, 6, 7, 8, 9, 10, 11, 12, 13 and 14.

### Moderator effects

The moderator effects of the variables described in the *Data Synthesis* section are reported as Q_B_ ​ statistics, which indicate whether differences between groups are moderated by the levels of these variables. The effect sizes for the differences between autistic and non-autistic groups, obtained from subsequent subgroup analyses, are presented in Tables 4 and 5.

**Table 5:** Summary of significant meta-analytic findings for amplitude moderator analyses (mixed-effects models fitted) – P/M50, P/M100, N100, N170, P/M200, N200, MMN/MMFfium

Amplitude	k^a^	Q_B_ (df, *p*)^b^	Q_W_ (df, *p*)^c^	SMD (*p* value)
*P*/M50				
Language impairment	39	29.09 (2, < 0.0001)	144.08 (37, < 0.0001)	
Yes	2			2.45 (< 0.0001)
No	37			−0.29 (0.20)
Medication	16	27.40 (3, < 0.0001)	164.32 (36, < 0.0001)	
Yes	5			−1.47 (0.07)
No	11			1.85 (0.03)
*N100*				
Modality	76	8.54 (3, 0.04)	144.42 (90, 0.0002)	
Visual	35			−0.27 (0.004)
Auditory	41			0.05 (0.57)
Combined	17			0.06 (0.74)
Age	93	9.08 (3, 0.03)	142.19 (90, 0.0004)	
Children	34			0.09 (0.33)
Adolescents	24			−0.27 (0.004)
Adults	35			−0.02 (0.84)
*N170*				
Task	15	11.94 (5, 0.04)	316.25 (145, < 0.0001)	
Target detection	99			−0.13 (0.24)
Oddball paradigm	6			−0.13 (0.73)
Passive task	22			0.19 (0.21)
Discrimination task	9			0.68 (0.01)
Face recognition task	14			0.32 (0.18)
*P/M200*				
Medication	19	16.07 (3, 0.001)	103.59 (61, 0.0005)	
Yes	7			0.02 (0.90)
No	12			−0.49 (< 0.0001)
N200				
Age	67	8.41 (3, 0.04)	215.82 (64, < 0.0001)	
Children	40			0.05 (0.71)
Adolescents	19			0.47 (0.02)
Adults	8			0.60 (0.08)
Year of publication	67	16.25 (6, 0.01)	198.55 (61, < 0.0001)	
2000–2012.DSM-IV.TR	46			0.47 (0.02)
2013–2024.DSM-V	21			0.11 (0.39)
Classification	67	16.25 (6, 0.01)	198.55 (61, < 0.0001)	
only DSM IV + DSM IV-TR (without ICD)	34			0.47 (0.002)
only DSM V (without ICD)	1			−0.45 (0.40)
only ICD 10 (without DSM)	8			−0.34 (0.19)
only ADOS/+ADIR (since 2013)	16			−0.01 (0.97)
DSM IV + DSM IV-TR + ICD 10	4			0.57 (0.09)
More than one DSM criteria/not mentioned which DSM criteria	4			0.38 (0.22)
Gender	67	6.59 (1, 0.01)	211.10 (65, < 0.0001)	0.02 (0.004)
*MMN/MMF*				
Quality rating	211	11.35 (1, 0.0008)	433.23 (209, < 0.0001)	0.08 (0.001)

Only significant results are presented here. For a complete list of results, including non-significant and unavailable data, please refer to the sTable 16 in the online resource. ^a^ k = number of included effect sizes. ^*b*^*Q*_*B*_ *=* Cochran’s Q statistic for moderator impact test. ^c^*Q*_*W*_ *=* Cochran’s Q statistic for heterogeneity test

#### Type of measurement (EEG/MEG)

A significant moderator-effect was found for P/M100-latency (Q_B_(2) = 10.41, *p* = 0.01) and MMN/MMF-latency (Q_B_(2) = 37.37, *p* < 0.0001), indicating significant differences for MEG-studies with longer latencies in autism.

#### Modality of sensory processing (auditory/visual/tactile/multisensory)

A significant moderator-effect was observed for N100-amplitude (Q_B_(3) = 8.54, *p* = 0.04) and P/M200-latency (Q_B_(3) = 20.54, *p* = 0.0001). Larger and significant mean effect sizes were found in visual tasks for these components. Additionally, a significant moderator effect was found for P/M100-latency (Q_B_(2) = 7.00,*p* = 0.03), with subgroup analyses indicating longer latencies in auditory tasks among autistic individuals.

#### Age groups (children/adolescents/adults)

Moderator analyses revealed significant differences across age groups for N100-amplitude (Q_B_(3) = 9.08, *p* = 0.03), N200-amplitude (Q_B_(3) = 8.41, *p* = 0.04), N170-latency (Q_B_(3) = 12.28, *p* = 0.01) and P/M200-latency (Q_B_(3) = 14.14, *p* = 0.003). Subgroup analyses showed a larger mean effect size for N100-amplitude in autistic adolescents and a smaller mean effect size for N200-amplitude in this group. Regarding N170 component, longer latencies were observed in autistic adolescents and adults and longer P/M200-latencies in autistic adults.

#### Language impairment (yes/no/unknown)

A significant moderator-effect was revealed for P/M50-amplitude (Q_B_(2) = 29.09, *p* < 0.0001), P/M50-latency (Q_B_(2) = 7.69, *p* = 0.02) and MMN/MMF-latency (Q_B_(2) = 8.20, *p* = 0.02) with larger mean effect sizes in autistic participants with language impairment. Moderator analyses also revealed significant differences between groups with and without language impairment for N170-latency (Q_B_(2) = 7.60, *p* = 0.02) and P/M200-latency (Q_B_(2) = 18.11, *p* = 0.0001). Subgroup analyses indicated longer N170-latencies and P/M200-latencies in autistic participants without language impairment only.

#### Task type (target-detection/oddball-paradigm/passive-task/discrimination-task/coherent motion-task/face-recognition-task/other(unknown))

Task type moderated effects for N170-amplitude (Q_B_(6) = 20.50, *p* = 0.002), N170-latency (Q_B_(6) = 59.63, *p* < 0.0001), and P/M200-latency (Q_B_(5) = 31.72, *p* < 0.0001). Subgroup analyses revealed smaller effect sizes for N170-amplitude in discrimination tasks, and longer latencies for N170 and P/M200 in target-detection and passive tasks.

#### Year of publication (1980–1986/1987–1993/1994–1999/2000–2012/2013–2024)

Significant moderator effects were found for N170-latency (Q_B_(2) = 12.47, *p* = 0.002) and P/M200-latency (Q_B_(3) = 9.83, *p* = 0.02), with larger mean effect sizes among autistic individuals in studies published between 2000 and 2012.

#### Co-occurring conditions in autism group (yes/no/unknown)

A significant moderator effect was observed for P/M100-latency (Q_B_(3) = 13.36, *p* = 0.004) with longer latencies reported in studies involving autistic participants without co-occurring conditions.

#### Medication in autism group (yes/no/unknown)

Medication status moderated effects for P/M50-amplitude (Q_B_(3) = 27.40, *p* < 0.001); autistic individuals taking medication showed a smaller effect size compared to non-medicated autistic individuals. The medication status also moderated effects for P/M200-amplitude (Q_B_(3) = 16.07, *p* = 0.001) observing a smaller effect size in non-medicated autistic individuals.

#### Classification system used for diagnosis (for grouping, see sMethods)

Significant moderator effects were found for N200-amplitude (Q_B_(6) = 16.25, *p* = 0.01) and P/M200-latency (Q_B_(4) = 14.43, *p* = 0.01). Smaller N200-amplitude and longer P/M200-latency differences between groups were observed in studies using DSM-IV/DSM-IV-TR criteria for autism diagnosis.

####  IQ

No significant moderator effects were observed.

#### Sex

Sex significantly moderated N200-amplitude results (Q_B_(1) = 6.59, *p* = 0.01), with smaller group differences between autistic and non-autistic individuals found in males.

#### Condition type (faces/objects)

Longer N170-latency (Q_B_(2) = 10.52, *p* = 0.01) in autistic individuals was observed for faces only.

Tables 5 and 6 present all significant moderator effects, structured by ERP/ERF components.

**Table 6:** Summary of significant meta-analytic findings for latency moderator analyses (mixed-effects models fitted) – P/M50, P/M100, N100, N170, P/M200, N200, MMN/MMFm

Latency	k	Q_B_ (df, *p*)	Q_W_ (df, *p*)	SMD (*p* value)
*P*/M50				
Language impairment	47	**7.69 (2**,** 0.02)**	181.66 (45, < 0.0001)	
Yes	12			0.72 (0.01)
No	35			0.38 (0.07)
*P/M100*				
EEG/MEG	195	10.41 (2, 0.01)	513.85 (185, < 0.0001)	
EEG	151			0.07 (0.45)
MEG	36			0.51 (0.002)
Modality	195	7.00 (2, 0.03)	498.79 (185, < 0.0001)	
Visual	115			0.06 (0.63)
Auditory	72			0.32 (0.01)
Co-occurring condition	111	13.36 (3, 0.004)	498.62 (184, < 0.0001)	
Yes	12			0.02 (0.95)
No	99			0.40 (0.002)
*N170*				
Age	119	12.28 (3, 0.01)	370.61 (116, < 0.0001)	
Children	62			0.08 (0.61)
Adolescents	18			0.63 (0.01)
Adults	39			0.47 (0.01)
Language impairment	119	7.60 (2, 0.02)	388.10 (117, < 0.0001)	
Yes	9			0.19 (0.68)
No	110			0.34 (0.01)
Task	115	15.51 (5, < 0.008)	213.27 (110, < 0.0001)	
Target detection	63			0.23 (0.04)
Oddball paradigm	6			0.07 (0.84)
Passive task	22			0.32 (0.02)
Discrimination task	10			−0.34 (0.18)
Face recognition task	4			0.42 (0.06)
Year of publication	119	12.47 (2, 0.002)	358.49 (117, < 0.0001)	
2000–2012.DSM-IV.TR	57			0.57 (0.001)
2013–2023.DSM-V	62			0.13 (0.41)
Condition type	118	10.52 (2, 0.01)	384.23 (116, < 0.0001)	
Objects	26			0.21 (0.15)
Faces	92			0.36 (0.004)
*P/M200*				
Modality	39	15.94 (2, 0.0003)	46.57 (37, 0.13)	
Visual	21			0.45 (< 0.0001)
Auditory	18			0.09 (0.45)
Age	41	14.14 (3, 0.003)	50.66 (38, 0.08)	
Children	19			0.19 (0.16)
Adolescents	11			0.25 (0.11)
Adults	11			0.55 (0.002)
Language impairment	41	18.11 (2, 0.0001)	48.97 (39, 0.13)	
Yes	12			−0.02 (0.92)
No	29			0.37 (< 0.0001)
Task	41	**31.72 (5**,** < 0.0001)**	40.66 (36, 0.27)	
Target detection	10			0.53 (0.0005)
Oddball paradigm	12			−0.09 (0.54)
Passive task	11			0.46 (0.0003)
Discrimination task	4			0.29 (0.06)
Face recognition task	4			0.30 (0.14)
Year of publication	41	9.83 (3, 0.02)	55.14 (38, 0.04)	
1994–1999.DSM-IV	8			0.13 (0.70)
2000–2012.DSM-IV.TR	17			0.38 (0.01)
2013–2023.DSM-V	16			0.27 (0.06)
Classification	41	14.43 (4, 0.01)	50.74 (37, 0.07)	
only DSM III + DSM III-TR (without ICD)	11			0.03 (0.89)
only DSM IV + DSM IV-TR (without ICD)	24			0.44 (0.0003)
only ADOS/+ADIR (since 2013)	5			0.21 (0.30)
More than one DSM criteria/not mentioned	1			−0.14 (0.75)
*MMN/MMF*				
EEG/MEG	156	37.37 (2, < 0.0001)	545.70 (154, < 0.0001)	
EEG	143			−0.13 (0.43)
MEG	13			2.54 (< 0.0001)
Language impairment	150	8.20 (2, 0.02)	673.53 (148, < 0.0001)	
Yes	36			0.67 (0.02)
No	114			0.13 (0.61)

Only significant results are presented here. For a complete list of results, including non-significant and unavailable data, please refer to the sTable 17 in online resource. ^a^ k = number of included effect sizes. ^*b*^*Q*_*B*_ *=* Cochran’s Q statistic for moderator impact test. ^c^*Q*_*W*_ *=* Cochran’s Q statistic for heterogeneity test

### Quality assessment analyses

The results of the quality assessment for each study are reported in sTable 1 of the online resource. To explore its potential impact on the findings, the quality rating was analysed as a continuous variable, derived from the sum of the two applied scales, and tested as a moderator. This analysis revealed a significant moderator effect of methodological quality on MMN-amplitude (Q_B_(1) = 11.35,*p* = 0.0008). Specifically, the analysis indicated that higher quality ratings were associated with larger MMN-amplitudes differences (see Table 5).

### Sensitivity analyses

Sensitivity analyses, excluding outliers, revealed additional significant results for N200-amplitude (SMD = 0.24), while P/M100-latency results became non-significant (SMD = 0.10). Complete results are available in the sTables 18–19 in the online resource.

### Publication bias analyses

Funnel plot asymmetry tests revealed potential publication bias for: P/M100-amplitude, N170-amplitude, P/M50-latency, N200-latency, and MMN/MMF-latency. Trim-and-fill analysis identified 18 missing studies for N200-amplitude and 15 for MMN/MMF-amplitude, but these adjustments did not affect their non-significant results. For P/M100-amplitude, the effect-size became significant (−0.15 to −0.07), while N170-amplitude shifted significantly in the opposite direction (−0.13 to 0.57). All funnel plots are provided in sFigs. 15, 16, 17, 18, 19, 20, and 21.

## Discussion

### Early ERPs and ERFs as potential brain-based biomarkers for autism

Sensory processing alterations are a key aspect of autism with growing recognition [55], affecting functioning and daily-life quality of autistic people [56]. This meta-analysis provides insights into the neurophysiological mechanisms and timings associated with sensory processing in autism by quantitatively synthesizing EEG and MEG studies comparing sensory processing in autistic and non-autistic children, adolescents, and adults and by exploring demographic and methodological moderators influencing between-group differences. Significant group-level differences between autistic and non-autistic individuals were found for the latencies of four ERP/ERF components: P/M50, P/M100, N170, and P/M200. As anticipated, substantial heterogeneity was observed and addressed by moderator-analysis.

With a moderate effect-size the earliest measured ERP/ERF component, the P/M50 yielded the most substantial alteration, indicating longer latencies in autistic individuals. The P/M50 is linked to filtering irrelevant stimuli and early sensory gating [36] and has been prominently studied as a potential biomarker for schizophrenia, where larger gating ratios and smaller amplitude differences reflect impaired sensory filtering of irrelevant stimuli [57–59]. In autism, this meta-analysis highlights delayed P/M50-latencies, not amplitudes, suggesting slower sensory processing rather than reduced filtering capacity. It has been hypothesized that, in autistic individuals, such delays may hinder real-time comprehension and contribute to sensory overwhelm, as slower arousal and delayed processing reduce the capacity to rapidly orient to and integrate novel input, thereby increasing the difficulty of coping with unexpected changes and promoting active avoidance as a means of maintaining predictability [60]. Moderator analysis showed even greater delays in individuals with co-occurring language impairment, leading to a notably large effect-size. This positions the P/M50 as a valuable biomarker-candidate for autistic individuals, especially with language impairment. Since language impairment is highly prevalent among autistic people [61], and affects academic, social, and adaptive skills [62], these delays may reflect underlying mechanisms to these alterations. Most of the reviewed studies primarily focused on children (of the 191 participants from studies including individuals with language impairment, 173 were children, 18 adolescents and none were adults), whose language and sensory gating abilities are still maturing [63]. These findings could help identify early predictors of potential differences, facilitating the provision of language support before autistic traits become apparent. This makes it an especially valuable approach, as early language support can enhance communication skills and support individual developmental pathways [64]. With eleven of the fourteen studies including auditory tasks and the strong association between language impairment and auditory processing [17, 65], auditory support in autistic individuals may provide significant benefits. Sensitivity analysis revealed slight publication bias in P/M50-latency, suggesting a lower true effect-size.

Prolonged P/M100-latencies in autism may reflect delayed attention to sensory inputs, including spatial information, indicating altered arousal regulation [38, 39]. These alterations were particularly evident in auditory tasks, although as a trend, with longer latencies in autistic individuals with language impairments. As with P/M50, the link between language impairment and auditory processing suggests sensory delays may contribute to communication alterations in autism. Since P/M100, like P/M50, is associated with auditory sensory gating [38], this result further supports the value of auditory support for autistic individuals with language impairment and sensory delays. Interestingly, autistic individuals without co-occurring conditions showed longer P/M100-latencies. ADHD, among the most common co-occurring condition in autism, is associated with shorter P100-latencies [66], which could partly explain this finding. The shift in P/M100-latency-effects after outlier removal highlights the need for cautious interpretation.

The N170-component is linked to face processing [41]. This meta-analysis confirms previous findings of prolonged N170-latency in autistic individuals [32, 67], showing greater delays for faces than objects. These delays may contribute to altered social behaviour, as face processing is closely tied to social interaction [68]. These findings highlight the potential to target face processing and social cognition in autism, which may improve social engagement and interactions. Moderator analysis revealed significant delays in autistic adolescents and adults but not children, suggesting slower face processing becomes more pronounced with age. A possible explanation is that autistic individuals from an early age spend less time engaged in face-to-face interactions or eye contact [69–71] impacting the development of brain regions otherwise specialised for face processing [72]. Supporting this, a computational model has demonstrated that reduced exposure to facial stimuli during critical developmental periods can impair the acquisition of face recognition abilities, suggesting that early altered experiences may have lasting consequences [73]. While there is not clear support for a strict critical period in face processing, there is some evidence of a sensitive period, with the face processing system showing early sensitivity to experience in the first year, remaining flexible until approximately 10–12 years of age, and becoming less sensitive to experience thereafter [74]. This highlights the importance of examining developmental changes in autism, as social cognitive alterations may be less apparent in early childhood but more measurable and diagnostically relevant with age. Thus, the N170 may be a valuable biomarker for social cognitive processing in autistic adolescents and adults. Alterations were found in both target-detection and passive tasks, though limited comparative studies constrain conclusions. Prolonged N170-latency was also noted in autistic individuals without language impairments, but again the limited number of studies constrains generalisability. The effect-size for N170 is smaller than that for P/M50, suggesting early sensory processing alterations in P/M50 may influence later face processing reflected in N170.

Furthermore, this meta-analysis showed prolonged P/M200-latencies in autistic individuals. Among others, P/M200 is linked to the processing of facial configuration [75] and decoding of verbal emotional expressions [42, 43]. Moderator analysis revealed these delays were significant in visual tasks, reflecting slower processing of social and emotional cues, which may contribute to challenges in social interaction and emotional understanding. Significant delays were observed in autistic adults specifically. Similar to the N170-component, this may be due to maturational processes and differing social experiences over time. Delays were noted in both, passive and target-detection tasks, and for autistic individuals without language impairment. However, cautious interpretation is warranted due to limited evidence.

Although some studies reported amplitude alterations [76, 77], this meta-analysis unexpectedly found none. Following Dinstein et al. 2015, one potential explanation may be that greater amplitude variability in autistic individuals might obscure group-level alterations [78]. This would be supported by quality assessment, sensitivity, and publication bias analyses conducted in our study, which revealed that several previously non-significant amplitude results became significant once these methodological factors were accounted for. However, this meta-analysis rather implies that while neural response magnitude may be similar across groups, response timing, reflected in latency alterations, may be a more robust marker for distinguishing autistic individuals.

Whenever differences between EEG and MEG were found in the moderator analysis, significant effect-sizes were observed only for MEG, suggesting it more reliably detects sensory alterations in early ERP/ERF components. Significant results in autism were observed only in auditory and visual tasks, suggesting core alterations in these domains. No alterations were found in tactile or multisensory tasks. However, the limited number of studies (2 for tactile and 5 for multisensory) allows only tentative conclusions. No eligible studies examined olfactory or gustatory functions, highlighting the need for future research in these areas. Significant differences between autistic and non-autistic individuals appeared in discrimination, target-detection, and passive tasks. However, k-values for other tasks were significantly smaller and often too small for reliable comparisons. Beyond P/M100-latency, co-occurring conditions had no effect on other components. Medication, sex and IQ had no moderator effect on overall significant components. However, medication and sex had moderator effects on not overall significant components (P/M50-, P/M200- and N200-amplitude, respectively, for details see online resource). Significant results in the quality assessment analysis of MMN/MMF-amplitudes suggest potential limitations in data reliability for this component, highlighting the need for cautious interpretation. The emergence of significant N200-amplitude differences after outlier removal suggests that extreme data points may have masked group differences in this component. Publication bias analysis revealed significant impacts on detecting effects for P/M100- and N170-amplitudes. For P/M100, the shift to significance suggests earlier analyses slightly underestimated its effect. For N170, a large shift from non-significance to significance indicates that pronounced bias obscured meaningful effects. Further exploration of these amplitudes is warranted.

### Clinical relevance and implications

ERPs/ERFs provide a non-invasive, cost-effective method to detect sensory processing alterations complementing self-reports or caregiver accounts and are generally easy to measure in clinical settings. P/M50-latencies in individuals with language impairment and N170-latencies in adolescents and adults could serve as potential biomarker-candidates. These neural markers could be used as diagnostic aid for early detection of sensory processing alterations. Furthermore, this evidence suggests that subgroups of autistic individuals may be identifiable based on alterations in P/M50 and N170, with P/M50 linked to language impairments and N170 observed in adolescents and adults addressing more social cognition alterations. This may not only enhance diagnostic precision but also guide more personalised support, better aligned with the sensory characteristics of these subgroups.

However, while objectively measuring sensory issues, ERPs/ERFs can only reveal sensory processing alterations on a neurophysiological level, which may not always align with sensory experiences described by autistic individuals or reported by caregivers. Additionally, the observed low effect-sizes and substantial heterogeneity limit the practical application as reliable biomarkers in clinical settings and routine diagnostics. Furthermore, studies are needed assessing the specificity of those markers and further exploring their value in clinical practice, enhancing and expanding traditional assessments.

### Limitations & future directions

Availability of studies varied substantially between ERP/ERF components and was too scarce for analysis in some cases. Similarly, the number of studies within each subgroup for moderator analysis varied considerably. Further, varying instruments assessing the degree of autism expression and sensory features in autistic individuals prohibited the exploration of these factors as moderators. Insufficient reporting of co-occurring conditions and medication, along with the exclusion of individuals with intellectual disabilities further limited analyses and generalizability. Finally, due to the imbalance in the sex of the participants and the fact that most studies in this field are conducted in Western countries, the results of this study may not be fully generalizable to female and other underrepresented participants or to populations outside Western contexts. Future studies should aim for a more balanced gender distribution and ensure inclusion of participants from diverse cultural and ethnic backgrounds, report co-occurring conditions and medications, include individuals with intellectual disabilities, and use standardized tools for degree of autism expression and questionnaires for sensory perception, to offer a more comprehensive understanding of sensory variations within the autistic community. They should further explore how sensory processing alterations affect emotional regulation, adaptive behaviour, and distress, offering insights into targeted support strategies in autism.

This meta-analysis included only peer-reviewed studies and preprints were excluded to maintain data quality. While this ensures that all findings underwent independent review, it may also introduce publication bias, as studies with significant results are more likely to be published [79, 80]. To evaluate this risk, a publication bias analysis was conducted, and authors were contacted to obtain unpublished findings, clarify graphical data, or resolve uncertainties in result interpretation. In total, 139 authors were approached, of whom around 40 (29%) responded, with 18 (13%) ultimately providing data that were incorporated into the analysis.

### Conclusions

This meta-analysis found prolonged P/M50-, P/M100-, N170-, and P/M200-latencies as potential biomarker-candidates for sensory processing alterations in autism. Prolonged P/M50-latency, reflecting sensory filtering challenges, could guide future studies on early autism detection, especially in individuals with language impairments. N170-latency findings suggest social cognition difficulties may not be evident in children but become more detectable in adolescents and adults, with small effect-sizes. However, low effect-sizes, heterogeneity, and limited number of studies constrain practical applications, warranting further research.

### Language

We adhered to the language guidelines proposed by AIMS-2-Trials, ensuring consistent and respectful terminology when describing autistic and non-autistic individuals [81].

## Supplementary Information

Below is the link to the electronic supplementary material.

Supplementary Material 1 (DOCX. 5.24 MB)

## Data Availability

No datasets were generated or analysed during the current study.
