# Supplementary material for: Neurophysiological alterations during sensory processing in autism - a meta-analysis
Source: Eur Child Adolesc Psychiatry. 2025 Nov 22;35(3):767–84. doi: 10.1007/s00787-025-02917-0 (PMC13212416; doi:10.1007/s00787-025-02917-0)
Supplement: Supplementary file 1 — Supplementary Material 1 (DOCX. 5.24 MB) [file 787_2025_2917_MOESM1_ESM.docx]

**Supplementary Material**

**Neurophysiological Alterations during Sensory Processing in Autism - A Meta-Analysis**

**European Child & Adolescent Psychiatry**

**Anjuli Ghosh** (0009-0006-9138-0177) **^1^ *, Natalia Nasarre-Nacenta ^1^ *, Sarah Baumeister** (0000-0001-9005-0084) **^1, 2^, ^3^ Nathalie E. Holz** (0000-0002-6225-9267) **^1, 2^, Tobias Banaschewski** (0000-0003-4595-1144) **^1, 2^, Daniel Brandeis ^1, 2, 4^, Pascal-M. Aggensteiner** (0000-0002-1048-9044) **^1, 2 ⴕ^, Anna Kaiser** (0009-0001-6373-8278) **^1, 2, 5 ⴕ^**

^1^ Department of Child and Adolescent Psychiatry and Psychotherapy, Central Institute of Mental Health, Medical Faculty Mannheim, Heidelberg University, Mannheim, Germany

^2^ German Center for Mental Health (DZPG), partner site Mannheim-Heidelberg-Ulm

^3^ School of Health and Social Sciences, AKAD University, Stuttgart, Germany

^4^ Department of Child and Adolescent Psychiatry and Psychotherapy, University Hospital of Psychiatry, University of Zurich, Zurich, Switzerland

^5^ Department of Child and Adolescent Psychiatry/Psychotherapy, University Hospital of Ulm, University of Ulm, Ulm, Germany

*share first authorship

^ⴕ^ share last authorship

**Corresponding author:**

Anna Kaiser

Department of Child and Adolescent Psychiatry and Psychotherapy

Central Institute of Mental Health

J 5 · 68159 Mannheim, Germany

Phone: +49 6211703 4939

anna.kaiser@zi-mannheim.de

Contents

[**sMethods** 4](#_Toc209443299)

[**Search strategy** 4](#_Toc209443300)

[**Data coding and data extraction** 5](#_Toc209443301)

[**Quality assessment** 9](#_Toc209443302)

[**sResults** 12](#_Toc209443303)

[**sTable 1. Characteristics of included studies** 12](#_Toc209443304)

[**sTable 2. Study Distribution and demographic characteristics displayed for P/M50 amplitude** 34](#_Toc209443305)

[**sTable 3. Study Distribution and demographic characteristics displayed for P/M50 latency** 34](#_Toc209443306)

[**sTable 4. Study Distribution and demographic characteristics displayed for P/M100 amplitude** 35](#_Toc209443307)

[**sTable 5. Study Distribution and demographic characteristics displayed for P/M100 latency** 36](#_Toc209443308)

[**sTable 6. Study Distribution and demographic characteristics displayed for P/M200 amplitude** 37](#_Toc209443309)

[**sTable 7. Study Distribution and demographic characteristics displayed for P/M200 latency** 38](#_Toc209443310)

[**sTable 8. Study Distribution and demographic characteristics displayed for N100 amplitude** 39](#_Toc209443311)

[**sTable 9. Study Distribution and demographic characteristics displayed for N100 latency** 39](#_Toc209443312)

[**sTable 10. Study Distribution and demographic characteristics displayed for N170 amplitude** 40](#_Toc209443313)

[**sTable 11. Study Distribution and demographic characteristics displayed for N170 latency** 41](#_Toc209443314)

[**sTable 12. Study Distribution and demographic characteristics displayed for N200 amplitude** 42](#_Toc209443315)

[**sTable 13. Study Distribution and demographic characteristics displayed for N200 latency** 43](#_Toc209443316)

[**sTable 14. Study Distribution and demographic characteristics displayed for MMN/MMF amplitude** 44](#_Toc209443317)

[**sTable 15. Study Distribution and demographic characteristics displayed for MMN/MMF latency** 44](#_Toc209443318)

[**Forest plots** 46](#_Toc209443319)

[**sFigure 1. Forest Plot from amplitude differences in P/M50 component between groups.** 46](#_Toc209443320)

[**sFigure 2. Forest Plot from latency differences in P/M50 component between groups.** 47](#_Toc209443321)

[**sFigure 3. Forest Plot from amplitude differences in P/M100 component between groups.** 48](#_Toc209443322)

[**sFigure 4. Forest Plot from latency differences in P/M100 component between groups.** 49](#_Toc209443323)

[**sFigure 5. Forest Plot from amplitude differences in P/M200 component between groups.** 50](#_Toc209443324)

[**sFigure 6. Forest Plot from latency differences in P/M200 component between groups.** 51](#_Toc209443325)

[**sFigure 7. Forest Plot from amplitude differences in N100 component between groups.** 52](#_Toc209443326)

[**sFigure 8. Forest Plot from latency differences in N100 component between groups.** 53](#_Toc209443327)

[**sFigure 9. Forest Plot from amplitude differences in N170 component between groups.** 54](#_Toc209443328)

[**sFigure 10. Forest Plot from latency differences in N170 component between groups.** 55](#_Toc209443329)

[55](#_Toc209443330)

[**sFigure 11. Forest Plot from amplitude differences in N200 component between groups.** 56](#_Toc209443331)

[**sFigure 12. Forest Plot from latency differences in N200 component between groups.** 57](#_Toc209443332)

[**sFigure 13. Forest Plot from amplitude differences in MMN/MMF component between groups.** 58](#_Toc209443333)

[**sFigure 14. Forest Plot from latency differences in MMN/MMF component between groups.** 59](#_Toc209443334)

[**Moderator analyses** 60](#_Toc209443335)

[**sTable 16. Summary of significant and non-significant meta-analytic findings for amplitude moderator analyses (mixed-effects models fitted).** 60](#_Toc209443336)

[**sTable 17. Summary of significant and non-significant meta-analytic findings for latency moderator analyses (mixed-effects models fitted).** 64](#_Toc209443337)

[**Sensitivity analyses** 68](#_Toc209443338)

[**sTable 18.Sensitivity analyses in amplitudes for each component** 68](#_Toc209443339)

[**sTable 19.Sensitivity analyses in latencies for each component** 69](#_Toc209443340)

[**Funnel Plots** 70](#_Toc209443341)

[**sFigure 15. Funnel Plot displaying meta-analytical results obtained from fitting multilevel models to P/M50 component amplitudes and latencies.** 70](#_Toc209443342)

[70](#_Toc209443343)

[**sFigure 16. Funnel Plot displaying meta-analytical results obtained from fitting multilevel models to P/M100 component amplitudes and latencies.** 70](#_Toc209443344)

[**sFigure 17. Funnel Plot displaying meta-analytical results obtained from fitting multilevel models to P/M200 component amplitudes and latencies.** 71](#_Toc209443345)

[**sFigure 18. Funnel Plot displaying meta-analytical results obtained from fitting multilevel models to N100 component amplitudes and latencies** 72](#_Toc209443346)

[**sFigure 19. Funnel Plot displaying meta-analytical results obtained from fitting multilevel models to N170 component amplitudes and latencies.** 72](#_Toc209443347)

[**sFigure sFigure 20. Funnel Plot displaying meta-analytical results obtained from fitting multilevel models to N200 component amplitudes and latencies.** 72](#_Toc209443348)

[**sFigure 21. Funnel Plot displaying meta-analytical results obtained from fitting multilevel models to MMN/MMF component amplitudes and latencies.** 73](#_Toc209443349)

[**References** 74](#_Toc209443350)

# **sMethods**

## **Search strategy**

PubMed

("autism spectrum disorder"[MeSH Terms] OR "autism s"[All Fields] OR "autisms"[All Fields] OR "autistic disorder"[MeSH Terms] OR ("autistic"[All Fields] AND "disorder"[All Fields]) OR "autistic disorder"[All Fields] OR "autism"[All Fields]) AND ("electroencephalography"[MeSH Terms] OR "electroencephalography"[All Fields] OR "eeg"[All Fields] OR "evoked potentials"[MeSH Terms] OR "magnetoencephalography"[MeSH Terms] OR "magnetoencephalography"[All Fields] OR "meg"[All Fields]) AND ("perception"[MeSH Terms] OR "perception"[All Fields] OR ("sensory"[All Fields] AND "processing"[All Fields]) OR "sensory"[All Fields])

PsycINFO

(MAINSUBJECT.EXACT("Autism Spectrum Disorders") OR "autism s" OR autisms OR "autistic disorder" OR autism) AND (MAINSUBJECT.EXACT("Electroencephalography") OR electroencephalography OR eeg OR MAINSUBJECT.EXACT("Evoked Potentials") OR MAINSUBJECT.EXACT("Magnetoencephalography") OR magnetoencephalography OR meg) AND (MAINSUBJECT.EXACT("Perception") OR perception OR (sensory AND processing) OR sensory)

Web Of Science

(ALL=("autism spectrum disorder") OR ALL=("autism s") OR ALL=(autisms) OR ALL=("autistic disorder") OR ALL=(autism)) AND (ALL=(electroencephalography) OR ALL=(eeg) OR ALL=("evoked potentials") OR ALL=(magnetoencephalography) OR ALL=(meg)) AND (ALL=(PERCEPTION) OR ALL=(SENSORY))

Clinical Trials Register

("autism s"[All Fields] OR "autisms"[All Fields] OR "autistic disorder"[MeSH Terms] OR ("autistic"[All Fields] AND "disorder"[All Fields]) OR "autistic disorder"[All Fields] OR "autism"[All Fields]) AND (("electroencephalography"[MeSH Terms] OR "electroencephalography"[All Fields] OR "eeg"[All Fields]) OR "meg"[All Fields]) AND ("perception"[MeSH Terms] OR "perception"[All Fields] OR ("sensory"[All Fields] AND "processing"[All Fields]) OR "sensory processing"[All Fields])

## **Data coding and data extraction**

The coding and part of the data extraction were performed using the Covidence [1] software.

Study type/reference

- First author
- Year of publication
- Peer-reviewed journal
- Study quality rating (QR)^[[1]](#footnote-1)^

Autistic group

- Sample size. n
- Mean age. years

1 child (< 12 years)

2 adolescent (12 – 17.99 years)

3 adult (≥ 18 years)

- Age range. years
- Handerness

1 right

2 left

3 ambidextrous

- Male. %
- Inteligence Quotient (IQ)
- Verbal IQ
- Non-verbal IQ
- Degree of autism expression (symptom severity questionnaire)
- Degree of autism expression (symptom severity score)
- Education. years
- Socioeconomical level
- Co-occuring condition

1 yes

2 no

3 no information

- Type of co-occuring condition
- Number of participants with co-occuring conditions
- Medication status

1 yes

2 no

3 no information

- Type of medication
- Number of participants medicated. %
- Socioeconomic status
- Classification system used for diagnosis

1 only DSM III+DSM III-TR (without ICD)

2 only DSM IV+DSM IV-TR (without ICD)

3 only DSM V (without ICD)

4 only ICD 10 (without DSM)

5 only ADOS/+ADIR (until year 2012)

6 only ADOS/+ADIR (since 2013)

7 DSM III+DSM III-TR+ICD 10

8 DSM IV+DSM IV-TR+ICD 10

9 DSM V+ICD 10

10 More than one DSM criteria/not mentioned which DSM criteria

- Pervasive developmental disorder (PDD)

1 yes

2 no

3 no information

- Language impairment

1 yes

2 no

3 only some participants with language impairment

- Sensory difficulties

1 mentioned

2 not mentioned

- Sensory profile
- 1 Low registration

2 Sensory seeking

3 Sensory sensitivity

4 Sensory avoidance

5 Total

6 Hyporesponsive

7 Hyperresponsive

8 Auditory item score

9 Touch score

Non-autistic group

- Sample size. n
- Mean age. years

1 child (< 12 years)

2 adolescent (12 – 17.99 years)

3 adult (≥ 18 years)

- Age range. years
- Handerness

1 right

2 left

3 ambidextrous

- Male. %
- IQ
- Verbal IQ
- Non-verbal IQ
- Education. years
- Socioeconomical level
- Co-occuring condition

1 yes

2 no

3 no information

- Type of co-occuring condition
- Number of participants with co-occuring conditions
- Medication status

1 yes

2 no

3 no information

- Type of medication
- Number of participants medicated. %
- Socioeconomic status
- Sensory difficulties

1 mentioned

2 not mentioned

- Sensory profile
- 1 Low registration

2 Sensory seeking

3 Sensory sensitivity

4 Sensory avoidance

5 Total

6 Hyporesponsive

7 Hyperresponsive

8 Auditory item score

9 Touch score

Moderators

- Type of measurement

1 EEG

2 MEG

- EEG/MEG system (type)
- Commercial EEG/MEG system (type)

1 yes

2 no

- Type of stimuli
- Task type category

1 Target detection

2 Oddball paradigm

3 Passive task

4 Discrimination task

5 Coherent motion task

6 Other tasks/not mentioned

7 Face recognition task

- Modality

1 visual

2 auditory

3 tactile

4 visual-auditory (multimodal)

5 visual-tactile (multimodal)

6 auditory-tactile (multimodal)

- Latency range
- Interstimulus intervals. ms
- Electrodes (type)

1 Ag/Ag-Cl electrodes

2 No information

3 Other

- Sensors (types)

1 Magnetometers

2 Gradiometers

3 No information

4 Other

- Number of total electrodes (chanels)/sensors
- Name of analyzed electrodes

1 Fz

2 Fcz

3 Cz

4 Cpz

5 Oz

6 Pz

7 Poz

8 Other

- Central midline electrodes assessed (only for EGG)

1 yes

2 no

- Region of Interest (only for MEG)

1 left anterior

2 right anterior

3 left posterior

4 right posterior

5 left occipital

6 right occipital

7 left hemisphere

8 right hemisphere

9 Other

- Region of Interest specific (only for MEG)
- Number of trials to compute average/ERPs
- Reference channel
- Impedance (only for EEG). Kohms
- Electrooculogram recorded

1 yes

2 no

- Eye movement correction

1 yes

2 no

- Artifact rejection criterion. uV
- Interpolation of noisy channels

1 yes

2 no

- Baseline correction

1 yes

2 no

- Filter range offline. Hz
- Montage/system (only for EEG)
- Reference (only for EEG)

1 Appropiate (Cz. FCz. vertex. mastoids....)

2 Other/inappropriate

- Number of coils (only MEG)
- Measure of SNR (only MEG)

ERP/F components

Coding of means (M) and standard deviations (SD) for all ERP/F components: P/M50. P/M100. P/M120. P/M130. P/M200. P/M220. P/M290. P/M300. P/M330. N80. N100. N130. N140. N160. N170. N200. N250. N270. N300. N350. Nc. MMN/MMF. ERP/ ERF. ERN. – amplitudes and latencies. Only those reported in more than 1 study where analyzed.

Performance data

Coding of means (M) and standard deviations (SD) for (behavioral) performance data.

## **Quality assessment**

Modified version of the Newcastle-Ottawa Scale (NOS) [2]

| **Item and responses** | **Points** |
| --- | --- |
| SELECTION | |
| **1. Is the case definition adequate?** | |
| 1. Yes. With independent validation (DSM, ICD, ADOS, ADI-R) | **1** |
| 1. Yes. E.g. record linkage or based on self reports | **0** |
| 1. No description | **0** |
| **2. Representativeness of the cases** | |
| 1. Consecutive or obviously representative series of cases | **1** |
| 1. Potential for selection biases not stated | **0** |
| **3. Selection of controls** | |
| 1. Community controls | **1** |
| 1. Hospital controls | **0** |
| 1. No description | **0** |
| **4. Definition of controls** | |
| 1. No current (psychiatric) diagnosis | **1** |
| 1. No description of source | **0** |
| COMPARABILITY | |
| 1. **Comparability (if matched)** | |
| 1. Study controls for age (most importante factor) | **1** |
| 1. Study controls for sex and IQ (any additional factors) | **1** |
| EXPOSURE | |
| 1. **Ascertainment of exposure** | |
| 1. Secure record | **1** |
| 1. Structured interview where blind to case/control status | **1** |
| 1. Interview not blinded to case/control status | **1** |
| 1. Written self report or medical record only | **0** |
| 1. No description | **0** |
| 1. **Same method of ascertainment for cases and controls** | |
| 1. Yes | **1** |
| 1. No | **0** |

Self-constructed scale for EEG and MEG signal quality

| **Item and responses** | **Points** |
| --- | --- |
| **SIGNAL QUALITY** | |
| **1. Impedance (only EEG)** | |
| 1. Below 20 kohms | **1** |
| 1. Below 50 kohms | **0** |
| 1. Above 50 Kohms | **0** |
| **2. Electrodes/sensors (type) (EEG/MEG)** | |
| 1. Ag/Ag-Cl electrodes | **1** |
| 1. Magnetometers/Gradiometers | **1** |
| 1. Other | **0** |
| **3. Central midline electrodes assessed (only EEG)** | **1** |
| **4. Montage/system (only EEG)** | |
| 1. 10-20 Internationl classification | **1** |
| 1. Other | **0** |
| **5. Number of trials to compute average/ERPs (EEG/MEG)** | |
| 1. minimum of 20 sweeps/trials* | **1** |
| 1. < 20 sweeps/trials or no information | **1** |
| **6. Electrooculogram recorded (EEG/MEG)** | **1** |
| **7. Eye movement correction performed (EEG/MEG)** | **1** |
| **8. Artifact rejection criterion (EEG)** | |
| <100 uV | **0** |
| 100 uV | **1** |
| 150 uV | **1** |
| 200 uV | **0** |
| > 200 uV | **0** |
| None | **0** |
| **Other quality control and data preprocessing strategies** | |
| **10a. Interpolation of noisy channels (EEG/MEG)** | **1** |
| **10b. Baseline correction (EEG/MEG)** | **1** |
| **10c. Reference (only for EEG)** | |
| 1. Appropriate* (Cz. FCz. vertex. mastoids. average...) | **1** |
| 1. Other/inappropriate | **0** |
| **10d. Is a filter range defined? (EEG/MEG)** | **1** |
| **ONLY MEG** | |
| **11. ROI defined** | **1** |
| **12. Number of COILS mentioned** | **1** |
| **9. Artifact rejection criterion mentioned (MEG)** | **1** |
| **Other added items** | |
| **1. Have they defined the component?** | **1** |
| **2. Have they non-significant data published or they send it tous?** | **1** |

*Note: The Newcastle-Ottawa Scale was modified by two experts in EEG/MEG research through joint discussion, to incorporate methodological quality criteria specific to EEG/MEG studies that are not addressed by the original NOS.*

# **sResults**

## **sTable 1. Characteristics of included studies**

| **Study ID** | **Age group** | **n**  **_TOTAL_** | **n_autistic_** | **Age. mean (years)** | **Male. mean (%)** | **IQ. mean** | **Co-occuring condition** | **Medication** | **Language impairment** | **n_non-autistic_** | **Age. mean (years)** | **Male. mean (%)** | **IQ mean** | **Co-occuring condition** | **Medication** | **Type** | **Modality** | **Task type/neuropsychological paradigm** | **TotalQR** |
| --- | --- | --- | --- | --- | --- | --- | --- | --- | --- | --- | --- | --- | --- | --- | --- | --- | --- | --- | --- |
| Abdeltawwab 2015 [3] | children | 61 | 31 | 11.37 | 77.42 | NA | NA | NA | yes | 30 | 11.20 | 60.00 | NA | NA | NA | EEG | auditory | Oddball paradigm | 11 |
| Ahlfors 2023 [4] | adolescences | 53 | 22 | 13.60 | 81.80 | NA | yes | NA | no | 31 | 13.10 | 87.10 | NA | no | NA | MEG | auditory | Passive task | 14 |
| Arnett 2018 [5] | adolescences | 103 | 76 | 12.12 | 80.26 | NA | NA | NA | yes | 27 | 13 | 62.96 | NA | no | NA | EEG | auditory | Passive task | 12 |
| Baruth 2010 [6] | adolescences | 30 | 15 | 13.90 | 86.67 | 92.50 | no | NA | no | 15 | 15.50 | 73.33 | NA | no | NA | EEG | visual | Target detection; Oddball paradigm | 15 |
| Batty 2011 [7] | children | 30 | 15 | 10.55 | 86.67 | NA | NA | NA | yes | 15 | 10.51 | 60.00 | NA | NA | NA | EEG | visual | Target detection | 11 |
| Borgolte 2021 [8] | adults | 29 | 14 | 40.30 | 50.00 | NA | Yes | NA | no | 15 | 42.40 | 60.00 | NA | no | NA | EEG | visual; auditory | Other: perception task | 18 |
| Brennan 2016 [9] | children | 25 | 12 | 9.30 | 91.67 | 98.40 | no | NA | no | 13 | 9.70 | 92.31 | 116.00 | no | NA | MEG | auditory | Passive task | 16 |
| **Study ID** | **Age group** | **n**  **_TOTAL_** | **n_autistic_** | **Age. mean (years)** | **Male. mean (%)** | **IQ. mean** | **Co-occuring condition** | **Medication** | **Language impairment** | **n_non-autistic_** | **Age. mean (years)** | **Male. mean (%)** | **IQ mean** | **Co-occuring condition** | **Medication** | **Type** | **Modality** | **Task type/neuropsychological paradigm** | **TotalQR** |
| Bruneau 1999 [10] | children | 32 | 16 | NA | 75.00 | NA | yes | NA | yes | 16 | NA | 75.00 | NA | no | NA | EEG | auditory | Passive task | 13 |
| Bruneau 2003 [11] | children | 42 | 26 | 5.92 | 84.62 | NA | no | no | yes | 16 | NA | 75.00 | NA | no | no | EEG | auditory | Passive task | 14 |
| Oram Cardy 2005 [12] | children | 16 | 7 | 11.90 | 100.00 | NA | NA | NA | no | 9 | 11.90 | 55.56 | NA | NA | NA | MEG | auditory | Oddball paradigm | 10 |
| Cary 2024 [13] | adolescences | 26 | 13 | 12.81 | 84.62 | 103.69 | no | NA | no | 13 | 12.53 | 46.15 | 115.08 | no | NA | EEG | auditory | Oddball paradigm; Passive task | 15 |
| Čeponiene 2003 [14] | children | 19 | 9 | 8.90 | 88.89 | NA | NA | no | yes | 10 | 8.40 | 90.00 | NA | NA | NA | EEG | auditory | Oddball paradigm | 12 |
| Charpentier 2018 [15] | children | 30 | 15 | 10.00 | 86.67 | NA | no | yes | no | 15 | 9.80 | 80.00 | NA | no | NA | EEG | auditory | Oddball paradigm; Passive task | 13 |
| Chen 2021 [16] | children | 48 | 24 | 7.60 | NA | NA | NA | no | no | 24 | 7.46 | NA | NA | NA | no | EEG | auditory | Passive task | 17 |
| Chien 2018 [17] | adults | 72 | 37 | 21.00 | 94.59 | 99.30 | NA | no | no | 35 | 20.50 | 91.43 | 110.60 | no | no | EEG | auditory | Oddball paradigm | 18 |
| Chien 2019 [18] | adults | 68 | 34 | 20.60 | 94.12 | 100.80 | NA | no | no | 34 | 20.40 | 94.12 | 110.50 | NA | no | EEG | auditory | Other: paired-click paradigm | 18 |
| Edgar 2014 [19] | children | 104 | 68 | 10.26 | NA | NA | NA | NA | no | 36 | 10.87 | NA | NA | NA | NA | MEG | auditory | Passive task | 14 |
| **Study ID** | **Age group** | **n**  **_TOTAL_** | **n_autistic_** | **Age. mean (years)** | **Male. mean (%)** | **IQ. mean** | **Co-occuring condition** | **Medication** | **Language impairment** | **n_non-autistic_** | **Age. mean (years)** | **Male. mean (%)** | **IQ mean** | **Co-occuring condition** | **Medication** | **Type** | **Modality** | **Task type/neuropsychological paradigm** | **TotalQR** |
| Churches 2012a [20] | adults | 22 | 11 | 31.82 | 100.00 | 120.18 | no | NA | no | 11 | 30.10 | 100.00 | 116.10 | no | NA | EEG | visual | Discrimination task | 13 |
| Churches 2012b [21] | adults | 23 | 10 | 30.61 | 100.00 | 115.20 | no | NA | no | 13 | 29.54 | 100.00 | 118.17 | no | NA | EEG | visual | Target detection | 14 |
| Cléry 2013a [22] | children | 24 | 12 | 11.58 | 83.33 | 86.00 | no | no | no | 12 | 11.25 | 83.33 | NA | no | no | EEG | visual | Oddball paradigm; Passive task | 16 |
| Cléry 2013b [23] | adults | 26 | 13 | 26.17 | 84.62 | 89.00 | NA | NA | no | 13 | 24.25 | 61.54 | NA | no | no | EEG | visual | Oddball paradigm; Passive task; Other: distractive task | 15 |
| Constable 2012 [24] | adults | 16 | 9 | 36.60 | 100.00 | 111.00 | NA | NA | no | 7 | 48.90 | 100.00 | 104 | NA | NA | EEG | visual | Passive task; Discrimination task | 11 |
| Cotter 2023 [25] | children | 216 | 84 | 11.40 | 85.70 | 101.50 | NA | no | no | 132 | 11.50 | 46.97 | 109.50 | no | no | EEG | auditory | Simple reaction time task. target detection | 17 |
| Crasta 2021 [26] | children | 36 | 18 | 8.41 | 72.22 | 98.72 | NA | NA | no | 18 | 8.03 | 72.22 | 111.39 | no | NA | EEG | auditory | Passive task; Other: Sensory Gating paradigm | 16 |
| Crasta 2023 [27] | adults | 48 | 24 | 23.31 | 70.80 | NA | NA | NA | no | 24 | 23.70 | 50.00 | NA | no | NA | EEG | auditory | modified paired-click sensory gating EEG paradigm | 15 |
| **Study ID** | **Age group** | **n**  **_TOTAL_** | **n_autistic_** | **Age. mean (years)** | **Male. mean (%)** | **IQ. mean** | **Co-occuring condition** | **Medication** | **Language impairment** | **n_non-autistic_** | **Age. mean (years)** | **Male. mean (%)** | **IQ mean** | **Co-occuring condition** | **Medication** | **Type** | **Modality** | **Task type/neuropsychological paradigm** | **TotalQR** |
| Dawson 2004 [28] | children | 51 | 29 | 3.73 | 89.66 | NA | no | NA | no | 22 | 3.64 | 86.36 | NA | no | NA | EEG | visual | Passive task; Other: Emotion recognition | 18 |
| Day 2024 [29] | adolescences | 126 | 49 | 14.50 | 85.70 | NA | NA | NA | no | 77 | 13.80 | 59.74 | NA | NA | NA | EEG | auditory | discrimination task | 15 |
| Demopoulos 2015 [30] | children | 37 | 25 | 11.47 | 72.00 | 84.16 | no | yes | yes | 12 | 13.78 | 58.33 | 111.08 | no | no | MEG | auditory | Passive task | 13 |
| Demopoulos 2017 [31] | children | 37 | 18 | 9.82 | 100.00 | NA | no | yes | no | 19 | 9.79 | 100.00 | NA | no | yes | MEG | auditory; tactile | Passive task | 17 |
| Donkers 2015 [32] | children | 67 | 28 | 7.62 | 78.57 | NA | no | yes | no | 39 | 7.03 | 79.49 | NA | no | no | EEG | auditory | Oddball paradigm; Passive task | 17 |
| Donkers 2020 [33] | children | 67 | 28 | 7.60 | 78.57 | NA | no | no | no | 39 | 7.00 | 76.92 | NA | no | no | EEG | auditory | Oddball paradigm; Passive task | 17 |
| Dunham-Carr 2023 [34] | children | 50 | 25 | 8.90 | NA | NA | NA | NA | no | 25 | 9.10 | NA | NA | NA | NA | EEG | auditory; visual | passive with attention task between trials | 14 |
| Dunn 2008 [35] | children | 68 | 34 | 9.29 | 73.53 | 93.71 | no | NA | yes | 34 | 9.50 | 58.82 | 115.41 | no | NA | EEG | auditory | Oddball paradigm | 17 |
| **Study ID** | **Age group** | **n**  **_TOTAL_** | **n_autistic_** | **Age. mean (years)** | **Male. mean (%)** | **IQ. mean** | **Co-occuring condition** | **Medication** | **Language impairment** | **n_non-autistic_** | **Age. mean (years)** | **Male. mean (%)** | **IQ mean** | **Co-occuring condition** | **Medication** | **Type** | **Modality** | **Task type/neuropsychological paradigm** | **TotalQR** |
| Dwyer 2021 [36] | children | 211 | 130 | 3.21 | 84.60 | 65.25 | NA | yes | no | 81 | 3.09 | 64.20 | 106.37 | NA | yes | EEG | auditory | passive task | 15 |
| Edgar 2015 [37] | children | 115 | 52 | 10.10 | 88.46 | 107.00 | no | yes | no | 63 | 9.80 | 92.06 | 112.60 | no | no | MEG | auditory | Passive task | 14 |
| Falter 2013 [38] | adults | 33 | 16 | 24.08 | 93.75 | 115.00 | no | no | no | 17 | 26.25 | 76.47 | 117 | no | no | MEG | visual | Discrimination task | 14 |
| Falter-Wagner 2024 [39] | adults | 33 | 15 | 26.83 | 93.30 | 113.00 | no | no | no | 18 | 26.50 | 83.33 | 116.00 | no | NA | MEG | visual | Discrimination task | 17 |
| Fan 2014 [40] | adults | 40 | 20 | 21.50 | 95.00 | 105.00 | no | NA | no | 20 | 22.00 | 95.00 | 107 | no | NA | EEG | auditory | Oddball paradigm; Passive task | 19 |
| Fanghella 2022 [41] | adults | 38 | 19 | 40.47 | 94.74 | NA | NA | NA | no | 19 | 40.84 | 94.74 | NA | NA | NA | EEG | visual; tactile | Passive task; Discrimination task; Other: emotion and gender tasks | 15 |
| Ferri 2003 [42] | adolescences | 20 | 10 | 12.30 | 100.00 | NA | yes | NA | no | 10 | 12.20 | 100.00 | NA | NA | NA | EEG | auditory | Oddball paradigm | 12 |
| Frey 2013 [43] | children | 51 | 22 | 11.30 | 95.45 | NA | NA | NA | no | 29 | 12.30 | 68.97 | NA | no | no | EEG | visual | Target detection; Other: control task | 13 |
| **Study ID** | **Age group** | **n**  **_TOTAL_** | **n_autistic_** | **Age. mean (years)** | **Male. mean (%)** | **IQ. mean** | **Co-occuring condition** | **Medication** | **Language impairment** | **n_non-autistic_** | **Age. mean (years)** | **Male. mean (%)** | **IQ mean** | **Co-occuring condition** | **Medication** | **Type** | **Modality** | **Task type/neuropsychological paradigm** | **TotalQR** |
| Fujita 2011 [44] | adults | 24 | 12 | 28.10 | 66.67 | 112.00 | NA | NA | no | 12 | 26.30 | 58.33 | NA | NA | NA | EEG | visual | Passive task; Other: memorizing task | 15 |
| Fujita 2013 [45] | adults | 19 | 9 | 30.90 | 77.78 | 105.70 | NA | Yes | no | 10 | 26.80 | 80.00 | NA | no | no | EEG | visual | Target detection | 13 |
| Gaetz 2017 [46] | children | 30 | 15 | 9.95 | 80.00 | NA | no | yes | no | 15 | 10.21 | 86.67 | NA | no | yes | MEG | tactile | Passive task | 15 |
| Gage 2003 [47] | children | 32 | 15 | 11.40 | 100.00 | NA | NA | NA | yes | 17 | 13.50 | 70.59 | NA | no | NA | MEG | auditory | Passive task | 9 |
| Gomot 2002 [48] | children | 30 | 15 | 6.83 | 80.00 | 57.00 | no | no | NA | 15 | 6.75 | 80.00 | NA | no | no | EEG | auditory | Oddball paradigm | 18 |
| Gomot 2011 [49] | children | 54 | 27 | 8.33 | 77.78 | 51.00 | no | no | no | 27 | 8.33 | 77.78 | NA | no | no | EEG | auditory | Oddball paradigm | 16 |
| Green 2020 [50] | children | 17 | 7 | 7.29 | 71.43 | NA | NA | NA | yes | 10 | 8.50 | 70.00 | NA | NA | NA | EEG | auditory | Oddball paradigm | 11 |
| Green 2023 [51] | children | 127 | 66 | 7.70 | 86.40 | NA | NA | no | no | 61 | 7.50 | 81.97 | NA | NA | no | MEG | auditory | Passive task | 14 |
| Grisoni 2019 [52] | adults | 42 | 20 | 38.00 | 55.00 | NA | NA | NA | no | 22 | 31.90 | 36.36 | NA | no | NA | EEG | auditory | Oddball paradigm; Passive task; Other: distraction-oddball paradigm | 14 |
| **Study ID** | **Age group** | **n**  **_TOTAL_** | **n_autistic_** | **Age. mean (years)** | **Male. mean (%)** | **IQ. mean** | **Co-occuring condition** | **Medication** | **Language impairment** | **n_non-autistic_** | **Age. mean (years)** | **Male. mean (%)** | **IQ mean** | **Co-occuring condition** | **Medication** | **Type** | **Modality** | **Task type/neuropsychological paradigm** | **TotalQR** |
| Gunji 2009 [53] | children | 17 | 8 | 10.80 | 87.50 | 97.00 | NA | NA | no | 9 | 11.30 | 44.44 | NA | no | NA | EEG | visual | Target detection | 13 |
| Gunji 2013 [54] | children | 18 | 9 | 7.50 | 100.00 | 83.00 | no | NA | no | 9 | 8.00 | 100.00 | NA | no | NA | EEG | visual | Target detection | 11 |
| Haigh 2022 [55] | adults | 52 | 24 | 28.50 | 79.17 | 111.71 | NA | NA | no | 28 | 28.70 | 67.86 | NA | no | no | EEG | auditory | Discrimination task; Other: Behavioral Pitch Discrimination Task | 17 |
| Hileman 2011 [56] | adolescences | 49 | 27 | 13.30 | 85.19 | >70 | no | NA | no | 22 | 14.39 | 81.82 | >70 | NA | NA | EEG | visual | Other: face processing task | 16 |
| Høyland 2019 [57] | adolescences | 98 | 49 | 15.60 | 73.47 | 91.90 | yes | yes | yes (1 participant) | 49 | 15.60 | 63.27 | NA | no | NA | EEG | visual | Other: Go-NoGo task | 18 |
| Huang 2018 [58] | children | 35 | 18 | 9.80 | 88.89 | NA | Yes | NA | yes | 17 | 9.40 | 88.24 | NA | no | NA | EEG | auditory | Oddball paradigm | 18 |
| Hudac 2018 [59] | adolescences | 133 | 102 | 12.29 | 80.39 | NA | NA | NA | no | 31 | 13.27 | 67.74 | NA | NA | NA | EEG | auditory | Oddball paradigm; Passive task | 12 |
| **Study ID** | **Age group** | **n**  **_TOTAL_** | **n_autistic_** | **Age. mean (years)** | **Male. mean (%)** | **IQ. mean** | **Co-occuring condition** | **Medication** | **Language impairment** | **n_non-autistic_** | **Age. mean (years)** | **Male. mean (%)** | **IQ mean** | **Co-occuring condition** | **Medication** | **Type** | **Modality** | **Task type/neuropsychological paradigm** | **TotalQR** |
| Isenstein 2022 [60] | adolescences | 31 | 15 | 15.08 | 73.33 | 97.02 | NA | NA | no | 16 | 13.66 | 31.25 | NA | no | NA | EEG | auditory | Passive task; Other: auditory habituation task | 12 |
| Ji 2019 [61] | children | 38 | 20 | 11.05 | 90.00 | NA | no | NA | no | 18 | 9.39 | 77.78 | NA | no | NA | EEG | visual | Discrimination task; Other: The tachistoscopic visual half-field paradigm | 15 |
| Jones 2018 [62] | children | 34 | 18 | 1.98 | NA | NA | NA | NA | no | 16 | 1.94 | NA | NA | NA | NA | EEG | visual | Passive task; Other: mother/stranger ERP paradigm | 12 |
| Kadlaskar 2021 [63] | children | 28 | 14 | 10.13 | 78.57 | NA | no | NA | no | 14 | 9.95 | 78.57 | NA | NA | NA | EEG | auditory; tactile | Oddball paradigm; Passive task | 17 |
| Kamita 2021 [64] | children | 30 | 15 | 9.07 | 86.67 | NA | no | NA | yes | 15 | NA | NA | NA | no | NA | EEG | auditory | Oddball paradigm | 10 |
| Kasai 2005 [65] | adults | 28 | 9 | 27.20 | 66.67 | 57.20 | no | yes | no | 19 | 27.30 | 68.42 | NA | no | NA | MEG | auditory | Oddball paradigm; Passive task | 16 |
| Kemner 2002 [66] | children | 23 | 12 | 10.40 | 83.33 | 96.20 | NA | no | no | 11 | 10.30 | 100.00 | 98.50 | NA | no | EEG | auditory | Passive task | 12 |
| Key 2014 [67] | children | 24 | 13 | 10.76 | 84.62 | 103.17 | no | no | no | 11 | 10.43 | 81.82 | 123.50 | no | no | EEG | visual | Target detection | 18 |
| **Study ID** | **Age group** | **n**  **_TOTAL_** | **n_autistic_** | **Age. mean (years)** | **Male. mean (%)** | **IQ. mean** | **Co-occuring condition** | **Medication** | **Language impairment** | **n_non-autistic_** | **Age. mean (years)** | **Male. mean (%)** | **IQ mean** | **Co-occuring condition** | **Medication** | **Type** | **Modality** | **Task type/neuropsychological paradigm** | **TotalQR** |
| Key 2024 [68] | adolescences | 47 | 25 | 14.53 | 80.00 | NA | NA | NA | no | 22 | 13.84 | 81.81 | NA | NA | NA | EEG | auditory | Discrimination task | 15 |
| Knight 2020 [69] | adolescences | 40 | 21 | NA | 90.48 | NA | no | yes | no | 19 | NA | 36.84 | NA | no | yes | EEG | auditory | Oddball paradigm | 15 |
| Korpilahti 2007 [70] | children | 27 | 14 | 11.20 | 100.00 | 110.00 | no | NA | no | 13 | 10.80 | 100.00 | NA | no | NA | EEG | auditory | Oddball paradigm; Passive task | 19 |
| Kovarski 2016 [71] | adults | 42 | 20 | 18.20 | 80.00 | NA | NA | NA | no | 22 | 18.40 | 77.27 | NA | NA | NA | EEG | visual | Passive task | 14 |
| Kovarski 2019 [72] | children | 36 | 18 | NA | 94.44 | NA | NA | NA | no | 18 | 7.50 | 83.33 | NA | NA | NA | EEG | visual | Passive task | 14 |
| Kröger 2014 [73] | children | 38 | 17 | 11.90 | 100.00 | 77.60 | no | NA | no | 21 | 11.63 | 100.00 | 59.10 | no | NA | EEG | visual | Discrimination task; Other: biological motion recognition | 17 |
| Kujala 2005 [74] | adults | 16 | 8 | 33.00 | 50.00 | 114.00 | no | no | no | 8 | 32 | 50.00 | NA | no | no | EEG | auditory | Target detection; Oddball paradigm; Passive task; Discrimination task; Other: stimulus-identification task | 12 |
| **Study ID** | **Age group** | **n**  **_TOTAL_** | **n_autistic_** | **Age. mean (years)** | **Male. mean (%)** | **IQ. mean** | **Co-occuring condition** | **Medication** | **Language impairment** | **n_non-autistic_** | **Age. mean (years)** | **Male. mean (%)** | **IQ mean** | **Co-occuring condition** | **Medication** | **Type** | **Modality** | **Task type/neuropsychological paradigm** | **TotalQR** |
| Kujala 2007 [75] | adults | 18 | 8 | 27.00 | 75.00 | 106.00 | no | no | no | 10 | 30 | 80.00 | 112 | no | no | EEG | auditory | Oddball paradigm; Other: multi-feature paradigm | 12 |
| Kujala 2010 [76] | children | 28 | 15 | 10.75 | 73.33 | 108.00 | no | no | no | 13 | 10.48 | 84.62 | 111 | no | NA | EEG | auditory | Other: multi-feature paradigm | 16 |
| Lacroix 2024 [77] | adults | 89 | 41 | 29.36 | 51.20 | 117.87 | yes | yes | no | 48 | 30.30 | 50.00 | 118.45 | yes | yes | EEG | visual | oddball paradigm. target detetion | 16 |
| Lambrechts 2018 [78] | adults | 36 | 18 | 25.25 | 94.44 | 112.00 | no | no | no | 18 | 26.33 | 72.22 | 116 | no | no | MEG | auditory | Discrimination task; Other: Duration and Pitch tasks | 16 |
| Lepistö 2005 [79] | children | 30 | 15 | 9.40 | 86.67 | NA | yes | no | yes (3 participants) | 15 | 9.40 | 86.67 | NA | no | NA | EEG | auditory | Oddball paradigm; Passive task | 17 |
| **Study ID** | **Age group** | **n**  **_TOTAL_** | **n_autistic_** | **Age. mean (years)** | **Male. mean (%)** | **IQ. mean** | **Co-occuring condition** | **Medication** | **Language impairment** | **n_non-autistic_** | **Age. mean (years)** | **Male. mean (%)** | **IQ mean** | **Co-occuring condition** | **Medication** | **Type** | **Modality** | **Task type/neuropsychological paradigm** | **TotalQR** |
| Lepistö 2006 [80] | children | 20 | 10 | 8.11 | 80.00 | NA | yes | no | no | 10 | 8.10 | 80.00 | NA | no | NA | EEG | auditory | Oddball paradigm; Discrimination task; Other: sound-identification task | 16 |
| Lepistö 2007 [81] | adults | 18 | 9 | 27.00 | 77.78 | NA | no | no | no | 9 | 30 | 88.89 | NA | NA | NA | EEG | auditory | Oddball paradigm | 14 |
| Lepistö 2008 [82] | children | 26 | 10 | 9.10 | 90.00 | NA | no | no | yes | 16 | 9 | 93.75 | NA | no | no | EEG | auditory | Oddball paradigm | 15 |
| Lepistö 2009 [83] | children | 30 | 16 | 8.10 | 81.25 | NA | no | no | no | 14 | 8.10 | 85.71 | NA | no | NA | EEG | auditory | Oddball paradigm; Other: Segregated and Integrated | 13 |
| Lincoln 1993 [84] | adolescences | 18 | 8 | 12.40 | NA | 71.10 | no | no | yes | 10 | 10.80 | NA | 108.60 | no | no | EEG | auditory | Oddball paradigm | 14 |
| Lincoln 1995 [85] | children | 19 | 9 | NA |  | >70 | no | no | yes | 10 | NA | NA | >70 | no | no | EEG | auditory | Passive task | 14 |
| Lindström 2016 [86] | children | 23 | 10 | 10.50 | 90.00 | NA | NA | no | yes | 13 | 10.00 | 92.31 | NA | no | NA | EEG | auditory | Oddball paradigm | 19 |
| Lindström 2018 [87] | children | 31 | 15 | 10.40 | 100.00 | NA | no | yes | no | 16 | 10.10 | 100.00 | NA | no | no | EEG | auditory | Oddball paradigm | 19 |
| **Study ID** | **Age group** | **n**  **_TOTAL_** | **n_autistic_** | **Age. mean (years)** | **Male. mean (%)** | **IQ. mean** | **Co-occuring condition** | **Medication** | **Language impairment** | **n_non-autistic_** | **Age. mean (years)** | **Male. mean (%)** | **IQ mean** | **Co-occuring condition** | **Medication** | **Type** | **Modality** | **Task type/neuropsychological paradigm** | **TotalQR** |
| Luckhardt 2017 [88] | adolescences | 37 | 21 | 12.50 | 95.24 | 103.40 | no | no | no | 16 | 13.00 | 81.25 | 106.90 | no | NA | EEG | visual | Target detection; Other: facial emotion recognition task | 15 |
| Ludlow 2014 [89] | adolescences | 22 | 11 | 13.00 | 100.00 | NA | no | NA | no | 11 | 13.70 | 100.00 | NA | no | NA | EEG | auditory | Oddball paradigm | 20 |
| Lv 2014 [90] | children | 70 | 39 | 5.79 | 94.87 | NA | no | NA | no | 31 | 6.06 | 74.19 | NA | NA | NA | EEG | auditory | Other: not mentioned | 13 |
| Maekawa 2011 [91] | adults | 18 | 9 | 28.00 | 77.78 | 107.00 | NA | NA | no | 9 | 28.90 | 44.44 | NA | NA | NA | EEG | visual | Oddball paradigm | 12 |
| Magnée 2008 [92] | adults | 25 | 12 | 21.50 | 100.00 | 122.40 | no | no | no | 13 | 23.00 | 100.00 | 127 | no | NA | EEG | visual; auditory | Discrimination task; Other: concurrent gender recognition task | 14 |
| Marco 2012 [93] | children | 14 | 7 | 9.40 | 100.00 | 84.40 | NA | no | no | 7 | 8.90 | 100.00 | 114.70 | NA | no | MEG | tactile | Oddball paradigm; Other: slow-rate stimuli paradigm and fast-rate stimuli paradigm | 13 |
| **Study ID** | **Age group** | **n**  **_TOTAL_** | **n_autistic_** | **Age. mean (years)** | **Male. mean (%)** | **IQ. mean** | **Co-occuring condition** | **Medication** | **Language impairment** | **n_non-autistic_** | **Age. mean (years)** | **Male. mean (%)** | **IQ mean** | **Co-occuring condition** | **Medication** | **Type** | **Modality** | **Task type/neuropsychological paradigm** | **TotalQR** |
| Marsicano 2023 [94] | children | 39 | 19 | 11.21 | 94.70 | 102.11 | no | no | no | 20 | 11.25 | 80.00 | NA | no | NA | EEG | visual | visual attentional task (target task) | 17 |
| Mason 2022 [95] | children | 88 | 50 | 9.75 | NA | NA | NA | NA | no | 38 | 10.04 | NA | NA | no | NA | EEG | visual | Passive task | 16 |
| Matsuzaki 2012 [96] | children | 21 | 9 | 9.64 | 100.00 | 102.17 | NA | NA | no | 12 | 10.08 | 100.00 | NA | no | NA | MEG | auditory | Other: NA | 12 |
| Matsuzaki 2019a [97] | adults | 38 | 19 | 23.80 | 100.00 | 108.36 | no | NA | no | 19 | 26.97 | 100.00 | 113.75 | no | NA | MEG | auditory | Passive task | 15 |
| Matsuzaki 2019b [98] | children | 48 | 21 | 10.67 | 85.71 | 83.88 | no | yes | yes | 27 | 10.14 | 92.59 | 112.96 | no | NA | MEG | auditory | Oddball paradigm | 14 |
| Matsuzaki 2019c [99] | adults | 25 | 9 | 22.22 | 100.00 | 106.22 | no | yes | no | 16 | 27.25 | 100.00 | 114.10 | no | no | MEG | auditory | Oddball paradigm | 16 |
| McPartland 2004 [100] | adults | 23 | 9 | 21.20 | 88.89 | NA | no | NA | no | 14 | 24.60 | 92.86 | NA | no | no | EEG | visual | Target detection | 14 |
| McPartland 2011 [101] | children | 54 | 36 | 11.20 | 88.89 | 105.20 | no | no | no | 18 | 12.60 | 83.33 | 112.90 | no | no | EEG | visual | Other: face recognition | 16 |
| Megnin 2012 [102] | adolescences | 28 | 14 | 16.90 | 100.00 | NA | no | NA | no | 14 | 16.90 | 100.00 | NA | no | NA | EEG | visual; auditory | Target detection | 19 |
| **Study ID** | **Age group** | **n**  **_TOTAL_** | **n_autistic_** | **Age. mean (years)** | **Male. mean (%)** | **IQ. mean** | **Co-occuring condition** | **Medication** | **Language impairment** | **n_non-autistic_** | **Age. mean (years)** | **Male. mean (%)** | **IQ mean** | **Co-occuring condition** | **Medication** | **Type** | **Modality** | **Task type/neuropsychological paradigm** | **TotalQR** |
| Molholm 2020 [103] | children | 99 | 45 | 9.40 | 91.11 | 102.30 | NA | NA | no | 54 | 9.30 | 59.26 | 110.80 | no | NA | EEG | visual; auditory | Target detection | 13 |
| Neuhaus 2016 [104] | children | 118 | 52 | 11.30 | NA | 97.40 | no | no | no | 66 | 10.10 | NA | 116.50 | no | no | EEG | visual | Target detection | 15 |
| O'Connor 2005 [105] | adults | 30 | 15 | 24.60 | NA | NA | no | yes | no | 15 | 24.80 | NA | NA | no | NA | EEG | visual | Other: Explicit face recognition task | 17 |
| O'Connor 2007 [106] | adults | 30 | 15 | 23.50 | 100.00 | NA | no | yes | no | 15 | 23.80 | 100.00 | NA | no | NA | EEG | visual | Target detection; Discrimination task | 17 |
| OramCardy 2004 [107] | children | 18 | 10 | 11.80 | 100.00 | NA | NA | NA | yes | 8 | 12.90 | 37.50 | NA | NA | NA | MEG | auditory | Passive task | 9 |
| OramCardy 2008 [108] | adolescences | 30 | 14 | NA | 85.71 | NA | NA | NA | no | 16 | NA | 50.00 | NA | NA | NA | MEG | auditory | Passive task | 12 |
| Orekhova 2008 [109] | children | 42 | 21 | 5.92 | 80.95 | NA | no | yes | no | 21 | 5.92 | 85.71 | NA | NA | NA | EEG | auditory | Other: paired clicks sensory gating paradigm | 16 |
| Parker 2021 [110] | children | 56 | 30 | 10.50 | 80.00 | 99.79 | no | no | no | 26 | 11.21 | 57.69 | 99.63 | no | no | EEG | visual | Target detection | 15 |
| **Study ID** | **Age group** | **n**  **_TOTAL_** | **n_autistic_** | **Age. mean (years)** | **Male. mean (%)** | **IQ. mean** | **Co-occuring condition** | **Medication** | **Language impairment** | **n_non-autistic_** | **Age. mean (years)** | **Male. mean (%)** | **IQ mean** | **Co-occuring condition** | **Medication** | **Type** | **Modality** | **Task type/neuropsychological paradigm** | **TotalQR** |
| Peristeri 2023[111] | adults | 24 | 10 | 21.80 | 20.00 | 98.40 | no | NA | no | 14 | 25.40 | 28.57 | 102.70 | no | NA | EEG | visual | picture naming task | 11 |
| Piatti 2021 [112] | children | 29 | 12 | 3.31 | 66.67 | 88.29 | NA | NA | no | 17 | 3.18 | 70.59 | NA | NA | NA | EEG | auditory | Oddball paradigm; Passive task | 16 |
| Portnova 2023 [113] | children | 50 | 25 | 4.84 | 56.00 | 87.20 | no | no | no | 25 | 5.25 | 52.00 | 90.10 | no | no | EEG | auditory | Passive task | 18 |
| Richards 2024 [114] | children | 63 | 35 | 5.45 | 97.10 | 66.00 | no | NA | no | 28 | 5.20 | 82.14 | 104.00 | NA | NA | EEG | visual | Passive task | 11 |
| Roberts 2010 [115] | children | 42 | 25 | 10.20 | NA | NA | no | yes | yes | 17 | 10.77 | NA | NA | no | NA | MEG | auditory | Passive task | 16 |
| Roberts 2011 [116] | children | 45 | 18 | 8.47 | 94.44 | 89.55 | no | NA | yes | 27 | 10065.00 | 44.44 | 109.25 | no | NA | MEG | auditory | Oddball paradigm; Passive task | 17 |
| Roberts 2019 [117] | children | 50 | 16 | 9.85 | 81.25 | NA | no | no | yes | 34 | 10.18 | 85.29 | 113.56 | no | NA | MEG | auditory | Passive task | 16 |
| Randeniya 2022 [118] | adults | 46 | 23 | 24.35 | 43.48 | NA | yes | yes | no | 23 | 24.04 | 47.83 | NA | no | no | EEG | visual; auditory | Oddball paradigm; Other: Stochastic frequency paradigm and a simultaneous.  visual 2-back tas | 19 |
| **Study ID** | **Age group** | **n**  **_TOTAL_** | **n_autistic_** | **Age. mean (years)** | **Male. mean (%)** | **IQ. mean** | **Co-occuring condition** | **Medication** | **Language impairment** | **n_non-autistic_** | **Age. mean (years)** | **Male. mean (%)** | **IQ mean** | **Co-occuring condition** | **Medication** | **Type** | **Modality** | **Task type/neuropsychological paradigm** | **TotalQR** |
| Ruiz-Martínez 2020 [119] | children | 31 | 16 | 8.96 | 93.75 | NA | NA | NA | yes | 15 | 8.86 | 93.33 | NA | no | NA | EEG | auditory | Oddball paradigm | 16 |
| Sakihara 2023 [120] | children | 17 | 12 | 11.00 | 83.30 | NA | no | no | no | 5 | 10.00 | 40.00 | NA | no | no | EEG | visual | Discrimination coherent motion | 14 |
| Schwartz 2023 [121] | adolescences | 78 | 40 | 15.94 | 72.50 | NA | NA | NA | yes | 38 | 14.75 | 55.26 | NA | no | NA | EEG | auditory | Passive task | 12 |
| Senju 2005 [122] | adolescences | 26 | 11 | 12.08 | 100.00 | NA | NA | NA | no | 15 | 12.08 | 86.67 | NA | NA | NA | EEG | visual | Target detection; Oddball paradigm | 12 |
| Shen 2016 [123] | children | 24 | 12 | 6.93 | 100.00 | NA | no | NA | yes | 12 | 7.05 | 100.00 | NA | no | NA | EEG | visual | Target detection | 15 |
| Shuffrey 2018 [124] | children | 32 | 16 | 8.04 | 68.75 | 105.00 | no | no | no | 16 | 9.44 | 56.25 | 110.56 | no | no | EEG | visual | Passive task | 15 |
| Sokhadze 2009 [125] | adolescences | 22 | 11 | 16.80 | 90.91 | 95.40 | no | NA | no | 11 | 19.40 | 81.82 | NA | no | NA | EEG | visual | Target detection; Oddball paradigm | 15 |
| Sokhadze 2012 [126] | adolescences | 32 | 16 | 12.60 | 87.50 | 95.35 | yes | yes | no | 16 | 14.60 | 81.25 | NA | no | NA | EEG | visual | Target detection; Oddball paradigm | 15 |
| **Study ID** | **Age group** | **n**  **_TOTAL_** | **n_autistic_** | **Age. mean (years)** | **Male. mean (%)** | **IQ. mean** | **Co-occuring condition** | **Medication** | **Language impairment** | **n_non-autistic_** | **Age. mean (years)** | **Male. mean (%)** | **IQ mean** | **Co-occuring condition** | **Medication** | **Type** | **Modality** | **Task type/neuropsychological paradigm** | **TotalQR** |
| Sokhadze 2016a [127] | adolescences | 60 | 30 | 15.63 | 73.33 | >80 | no | no | no | 30 | 15.76 | 76.67 | NA | no | no | EEG | visual | Target detection; Coherent motion; Other: Cued Posner spatial attention task | 13 |
| Sokhadze 2016b [128] | children | 32 | 18 | 11.06 | 83.33 | NA | no | NA | no | 14 | 12.60 | 85.71 | NA | no | NA | EEG | auditory | Oddball paradigm; Passive task | 9 |
| Tanaka 2023 [129] | adults | 15 | 7 | 36.00 | NA | 101.10 | NA | NA | no | 8 | 35.90 | NA | 112.60 | NA | NA | EEG | visual | Target detection | 15 |
| Tavares 2016 [130] | adults | 25 | 9 | 23.10 | 100.00 | 95.20 | NA | yes | no | 16 | 23.40 | 100.00 | 103.10 | NA | NA | EEG | visual | Discrimination task; Other: Decision task | 16 |
| Toffoli 2021 [131] | children | 86 | 29 | 11.04 | 75.86 | 110.90 | NA | NA | no | 57 | 10.50 | 56.14 | 113.53 | NA | NA | EEG | visual | Coherent motion | 14 |
| vanLaarhoven 2019 [132] | adults | 60 | 30 | 18.55 | 73.33 | 103.00 | no | no | no | 30 | 18.83 | 80.00 | 111.97 | no | no | EEG | auditory | Other: motor-auditory task | 20 |
| Vlaskamp 2017 [133] | children | 73 | 35 | 11.10 | 80.00 | 98.50 | no | yes | no | 38 | 10.90 | 71.05 | 107.60 | no | NA | EEG | auditory | Oddball paradigm; Passive task | 15 |
| **Study ID** | **Age group** | **n**  **_TOTAL_** | **n_autistic_** | **Age. mean (years)** | **Male. mean (%)** | **IQ. mean** | **Co-occuring condition** | **Medication** | **Language impairment** | **n_non-autistic_** | **Age. mean (years)** | **Male. mean (%)** | **IQ mean** | **Co-occuring condition** | **Medication** | **Type** | **Modality** | **Task type/neuropsychological paradigm** | **TotalQR** |
| Wagner 2013 [134] | adolescences | 38 | 18 | 17.00 | 100.00 | 111.20 | yes | yes | no | 20 | 17.90 | 100.00 | 116.50 | no | no | EEG | visual | Target detection | 16 |
| Wang 2017 [135] | children | 31 | 16 | 10.40 | NA | NA | NA | NA | no | 15 | 10.30 | NA | NA | NA | NA | EEG | auditory | Oddball paradigm; Passive task | 17 |
| Weismüller 2015 [136] | children | 33 | 18 | 9.40 | 100.00 | 99.30 | no | yes | no | 15 | 10.60 | 100.00 | 118.30 | no | NA | EEG | auditory | Oddball paradigm; Passive task | 16 |
| Webb 2006 [137] | children | 45 | 27 | 3.80 | NA | NA | NA | NA | no | 18 | 3.70 | NA | NA | NA | NA | EEG | visual | Passive task | 11 |
| Whitehouse 2008 [138] | children | 30 | 15 | 10.40 | 100.00 | NA | no | NA | no | 15 | 10.60 | 73.33 | NA | no | NA | EEG | auditory | Oddball paradigm; Passive task; Discrimination task | 15 |
| Yamasaki 2011 [139] | adults | 24 | 12 | NA | 75.00 | 100.30 | NA | NA | no | 12 | NA | 75.00 | NA | NA | NA | EEG | visual | Passive task; Coherent motion | 14 |
| Yamasaki 2017 [140] | adults | 28 | 14 | 29.90 | 64.29 | 104.90 | NA | NA | no | 14 | 28.60 | 64.29 | 113.10 | no | NA | EEG | visual | Passive task | 15 |
| Yoshimura 2016 [141] | children | 70 | 35 | 6.29 | 77.14 | 95.60 | NA | NA | no | 35 | 6.23 | 77.14 | 99.40 | NA | NA | MEG | auditory | Oddball paradigm; Passive task | 13 |
| Yoshimura 2021 [142] | children | 75 | 29 | 6.23 | 72.41 | 91.40 | NA | NA | no | 46 | 5.86 | 89.13 | 104.70 | NA | NA | MEG | auditory | Oddball paradigm; Passive task | 13 |
| Yu 2015a1 [143] | children | 34 | 18 | 9.30 | 88.89 | NA | no | NA | yes | 16 | 9.50 | 81.25 | NA | no | NA | EEG | auditory | Oddball paradigm; Passive task | 17 |
| Yu 2015a2 [143] | children | 34 | 16 | 9.60 | 93.75 | NA | no | NA | yes | 18 | 9.30 | 66.67 | NA | no | NA | EEG | auditory | Oddball paradigm; Passive task | 17 |
| Yu 2018 [144] | children | 31 | 15 | 9.60 | 93.33 | NA | no | no | yes | 16 | 9.80 | 81.25 | NA | no | no | EEG | auditory | Oddball paradigm; Passive task | 16 |
| Yu 2021 [145] | adolescences | 49 | 22 | 12.10 | 90.91 | NA | NA | NA | yes | 27 | 11.70 | 77.78 | NA | NA | NA | EEG | auditory | Passive task | 13 |

Note. QR = Quality rating

## **sTable 2. Study Distribution and demographic characteristics displayed for P/M50 amplitude**

| **P/M50 amplitude** | | | | | |
| --- | --- | --- | --- | --- | --- |
|  | **k** | **Autistic n** | **Non-autistic n** | **Total n** | |
| Total | 14 | 321 | 268 | 589 | |
| EEG | 8 | 182 | 173 | 355 | |
| MEG | 6 | 130 | 95 | 225 | |
| Auditory | 9 | 238 | 159 | 397 | |
| Visual | 1 | 15 | 15 | 30 | |
| Tactile | 2 | 22 | 22 | 44 | |
| Combined modalities | 2 | 37 | 38 | 75 | |
| Children | 10 | 220 | 176 | 396 | |
| Adolescents | 1 | 15 | 15 | 30 | |
| Adults | 3 | 77 | 77 | 154 | |
|  | **Autistic** | **Non-autistic** | **t** | **df** | **p** |
| **Total** | | | | | |
| Age (years). M (SD) | 13,36 (9,17) | 14,23 (10,08) | -1.09 | 546.08 | 0.28 |
| Male (%). M (SD) | 87,23 (9,35) | 82,46 (16,17) | 4.27 | 410.41 | **<0.001** |
| IQ. M (SD) | 97,63 (17,17) | 110,67 (13,30) | -6.91 | 253.51 | **<0.001** |
| **Children** | | | | | |
| Age (years). M (SD) | 8,95 (2,83) | 8,76 (2,70) | 0.70 | 385.00 | 0.48 |
| Male (%). M (SD) | 87,34 (9,10) | 84,09 (14,21) | 2.64 | 281.17 | **0.01** |
| IQ. M (SD) | 97,35 (17,52) | 110,75 (14,43) | -5.58 | 166.44 | **<0.0001** |
| **Adolescents** | | | | | |
| Age (years). M (SD) | 13,9 (3,45) | 15,5 (4,21) | -1.14 | 26.96 | 0.26 |
| Male (%). M (SD) | 86,67 (0) | 73,33 (0) | NA | NA | NA |
| IQ. M (SD) | 92,5 (15,1) | NA | NA | NA | NA |
| **Adults** | | | | | |
| Age (years). M (SD) | 26,35 (9,89) | 26,47 (10,71) | -0.07 | 151 | 0.94 |
| Male (%). M (SD) | 87,01 (10,89) | 80,52 (20,54) | 2.45 | 116 | **0.02** |
| IQ. M (SD) | 100,80 (16,20) | 110,50 (10,60) | -2.92 | 57 | **0.005** |

*Note:* k = number of studies, n = number of participants

## **sTable 3. Study Distribution and demographic characteristics displayed for P/M50 latency**

| **P/M50 latency** | | | | | |
| --- | --- | --- | --- | --- | --- |
|  | **k** | **Autistic n** | **Non-autistic n** | **Total n** | |
| Total | 14 | 486 | 420 | 906 | |
| EEG | 4 | 99 | 91 | 190 | |
| MEG | 10 | 387 | 329 | 716 | |
| Auditory | 11 | 438 | 371 | 809 | |
| Visual | 1 | 15 | 15 | 30 | |
| Tactile | 1 | 15 | 15 | 30 | |
| Combined modalities | 1 | 18 | 19 | 37 | |
| Children | 11 | 428 | 362 | 790 | |
| Adolescents | 1 | 15 | 15 | 30 | |
| Adults | 2 | 43 | 43 | 86 | |
|  | **Autistic** | **Non-autistic** | **t** | **df** | **p** |
| **Total** | | | | | |
| Age (years). M (SD) | 10.17 (4.54) | 10.47 (5.39) | -0.95 | 915.90 | 0.34 |
| Male (%). M (SD) | 87.24 (6.70) | 82.38 (12.40) | 7.18 | 622.58 | 2.01 |
| IQ. M (SD) | 102.64 (20.54) | 113.05 (14.73) | -5.53 | 347.70 | 6.31 |
| **Children** | | | | | |
| Age (years). M (SD) | 9.06 (2.31) | 8.76 (2.29) | 2.01 | 897.15 | **0.04** |
| Male (%). M (SD) | 87.62 (5.33) | 83.98 (9.32) | 6.58 | 553.08 | 1.12 |
| IQ. M (SD) | 102.90 (20.73) | 112.95 (14.61) | -4.90 | 291.08 | 1.59 |
| **Adolescents** | | | | | |
| Age (years). M (SD) | 13.9 (3.45) | 15.5 (4.21) | -1.14 | 26.96 | 0.26 |
| Male (%). M (SD) | 86.67 (0) | 73.3 (0) | NA | NA | NA |
| IQ. M (SD) | 92.5 (15.1) | NA | NA | NA | NA |
| **Adults** | | | | | |
| Age (years). M (SD) | 23.53 (5.03) | 25.14 (3.20) | -1.78 | 71.22 | 0.08 |
| Male (%). M (SD) | 83.72 (14.48) | 72.09 (24.83) | 2.65 | 67.62 | **0.01** |
| IQ. M (SD) | 108.36 (19.81) | 113.75 (15.75) | -0.93 | 34.05 | 0.36 |

*Note:* k = number of studies, n = number of participants

## **sTable 4. Study Distribution and demographic characteristics displayed for P/M100 amplitude**

| **P/M100 amplitude** | | | | | |
| --- | --- | --- | --- | --- | --- |
|  | **k** | **Autistic n** | **Non-autistic n** | **Total n** | |
| Total | 59 | 1205 | 1118 | 2323 | |
| EEG | 54 | 1059 | 1000 | 2059 | |
| MEG | 5 | 146 | 118 | 264 | |
| Auditory | 26 | 733 | 664 | 1397 | |
| Visual | 30 | 394 | 367 | 761 | |
| Tactile | 0 | 0 | 0 | 0 | |
| Combined modalities | 3 | 78 | 87 | 165 | |
| Children | 35 | 840 | 807 | 1647 | |
| Adolescents | 11 | 224 | 169 | 393 | |
| Adults | 13 | 141 | 142 | 283 | |
|  | **Autistic** | **Non-autistic** | **t** | **df** | **p** |
| **Total** | | | | | |
| Age (years). M (SD) | 12.78 (836.96) | 13.22 (8.21) | -0.02 | 1678.35 | 0.98 |
| Male (%). M (SD) | 85.58 (9.38) | 72.00 (16.77) | 24.22 | 1812.51 | **<0.0001** |
| IQ. M (SD) | 92.87 (24.83) | 108.40 (17.27) | -14.38 | 1474.89 | **<0.000** |
| **Children** | | | | | |
| Age (years). M (SD) | 9.32 (2.49) | 9.57 (2.44) | -2.91 | 1.976.98 | **0.004** |
| Male (%). M (SD) | 86.53 (8.71) | 77.94 (15.80) | 22.04 | 1.312.47 | **<0.0001** |
| IQ. M (SD) | 88.72 (26.62) | 113.06 (14.25) | -14.92 | 1.178.49 | **<0.0001** |
| **Adolescents** | | | | | |
| Age (years). M (SD) | 14.07 (3.04) | 14.52 (3.12) | -1.87 | 614.867 | **0.06** |
| Male (%). M (SD) | 86.61 (7.21) | 78.23 (15.42) | 6.16 | 188.34 | **<0.0001** |
| IQ. M (SD) | 104.29 (15.46) | 112.99 (12.35) | -3.31 | 109.91 | **0.001** |
| **Adults** | | | | | |
| Age (years). M (SD) | 26.35 (9.70) | 26.99 (8.57) | -0.01 | 302.01 | 0.99 |
| Male (%). M (SD) | 85.11 (12.02) | 71.58 (18.53) | 8.04 | 323.63 | **0.0001** |
| IQ. M (SD) | 112.27 (14.39) | 115.92 (11.69) | -1.97 | 196.58 | 0.05 |

*Note:* k = number of studies, n = number of participants

## **sTable 5. Study Distribution and demographic characteristics displayed for P/M100 latency**

| **P/M100 latency** | | | | | |
| --- | --- | --- | --- | --- | --- |
|  | **k** | **Autistic n** | **Non-autistic n** | **Total n** | |
| Total | 52 | 1075 | 975 | 2050 | |
| EEG | 38 | 735 | 652 | 1387 | |
| MEG | 14 | 340 | 323 | 663 | |
| Auditory | 26 | 682 | 604 | 1286 | |
| Visual | 26 | 393 | 371 | 764 | |
| Tactile | 0 | 0 | 0 | 0 | |
| Combined modalities | 0 | 0 | 0 | 0 | |
| Children | 33 | 802 | 691 | 1493 | |
| Adolescents | 6 | 106 | 98 | 204 | |
| Adults | 12 | 167 | 186 | 353 | |
|  | **Autistic** | **Non-autistic** | **t** | **df** | **p** |
| **Total** | | | | | |
| Age (years). M (SD) | 12.35 (7.57) | 12.89 (8.15) | -1.83 | 2.810.06 | 0.07 |
| Male (%). M (SD) | 84.09 (11.56) | 77.74 (14.56) | 10.85 | 1.856.17 | 1.18 |
| IQ. M (SD) | 93.26 (25.45) | 108.19 (18.06) | -13.30 | 1.483.95 | 3.39 |
| **Children** | | | | | |
| Age (years). M (SD) | 8.28 (3.51) | 8.20 (3.34) | 0.55 | 1931.083 | 0.58 |
| Male (%). M (SD) | 84.66 (9.27) | 78.15 (13.29) | 10.81 | 1.207.07 | 4.65 |
| IQ. M (SD) | 88.61 (25.85) | 106.28 (19.03) | -13.46 | 1.160.90 | 1.79 |
| **Adolescents** | | | | | |
| Age (years). M (SD) | 14.34 (2.76) | 14.52 (3.08) | -0.63 | 367.49 | 0.53 |
| Male (%). M (SD) | 87.74 (5.48) | 80.61 (1.76) | 12.69 | 127.95 | 2.12 |
| IQ. M (SD) | 100.65 (12.47) | 106.90 (6.55) | -2.28 | 45.82 | **0.03** |
| **Adults** | | | | | |
| Age (years). M (SD) | 25.66 (7.18) | 26.77 (6.96) | -1.87 | 569.83 | 0.06 |
| Male (%). M (SD) | 79.04 (19.83) | 74.73 (20.96) | 1.98 | 350.03 | 0.05 |
| IQ. M (SD) | 111.60 (14.92) | 115.43 (12.73) | -2.36 | 288.33 | **0.02** |

*Note:* k = number of studies, n = number of participants

## **sTable 6. Study Distribution and demographic characteristics displayed for P/M200 amplitude**

| **P/M200 amplitude** | | | | | |
| --- | --- | --- | --- | --- | --- |
|  | **k** | **Autistic n** | **Non-autistic n** | **Total n** | |
| Total | 22 | 528 | 606 | 1134 | |
| EEG | 20 | 492 | 569 | 1061 | |
| MEG | 2 | 36 | 37 | 73 | |
| Auditory | 10 | 307 | 381 | 688 | |
| Visual | 8 | 119 | 112 | 231 | |
| Tactile | 0 | 0 | 0 | 0 | |
| Combined modalities | 4 | 102 | 113 | 215 | |
| Children | 9 | 258 | 311 | 569 | |
| Adolescents | 6 | 155 | 179 | 334 | |
| Adults | 7 | 115 | 116 | 231 | |
|  | **Autistic** | **Non-autistic** | **t** | **df** | **p** |
| **Total** | | | | | |
| Age (years). M (SD) | 13.98 (7.59) | 13.75 (7.52) | 0.51 | 1107.87 | 0.61 |
| Male (%). M (SD) | 85.18 (10.08) | 85.18 (10.08) | 22.42 | 886.70 | **<0.0001** |
| IQ. M (SD) | 100.60 (18.35) | 100.60 (18.35) | -7.58 | 452.00 | **<0.0001** |
| **Children** | | | | | |
| Age (years). M (SD) | 9.18 (3.17) | 9.61 (3.22) | -1.58 | 550.56 | 0.12 |
| Male (%). M (SD) | 89.29 (7.31) | 89.29 (7.31) | 19.19 | 356.82 | **<0.0001** |
| IQ. M (SD) | 101.4 (17.91) | 101.4 (17.91) | -5.15 | 226.57 | **<0.0001** |
| **Adolescents** | | | | | |
| Age (years). M (SD) | 15.40 (3.06) | 14.48 (3.50) | 1.56 | 331.97 | 0.12 |
| Male (%). M (SD) | 80.65 (6.44) | 61.45 (13.39) | 17.04 | 264.06 | **<0.0001** |
| IQ. M (SD) | 94.93 (20.82) | NA | NA | NA | NA |
| **Adults** | | | | | |
| Age (years). M (SD) | 23.31 (8.76) | 23.71 (9.47) | -0,34 | 227,92 | 0,73 |
| Male (%). M (SD) | 83 (15.12) | 74.26 (16.24) | 3,95 | 198,25 | **0,00** |
| IQ. M (SD) | 101.94 (17.33) | 112.40 (10.12) | -4,48 | 135,88 | **<0,0001** |

*Note:* k = number of studies, n = number of participants

## **sTable 7. Study Distribution and demographic characteristics displayed for P/M200 latency**

| **P/M200 latency** | | | | | |
| --- | --- | --- | --- | --- | --- |
|  | **k** | **Autistic n** | **Non-autistic n** | **Total n** | |
| Total | 14 | 264 | 270 | 534 | |
| EEG | 13 | 246 | 251 | 497 | |
| MEG | 1 | 18 | 19 | 37 | |
| Auditory | 6 | 153 | 165 | 318 | |
| Visual | 7 | 93 | 86 | 179 | |
| Tactile | 0 | 0 | 0 | 0 | |
| Combined modalities | 1 | 18 | 19 | 37 | |
| Children | 6 | 92 | 72 | 164 | |
| Adolescents | 4 | 104 | 130 | 234 | |
| Adults | 4 | 68 | 68 | 136 | |
|  | **Autistic** | **Non-autistic** | **t** | **df** | **p** |
| **Total** | | | | | |
| Age (years). M (SD) | 13.61 (6.46) | 13.08 (5.82) | 1.06 | 577.90 | 0.29 |
| Male (%). M (SD) | 87.88 (7.91) | 74.81 (19.90) | 10.01 | 353.52 | 6.19 |
| IQ. M (SD) | 97.89 (19.12) | NA | NA | NA | NA |
| **Children** | | | | | |
| Age (years). M (SD) | 7.44 (3.06) | 6.14 (2.97) | 3.02 | 195.98 | **0.00** |
| Male (%). M (SD) | 94.57 (5.76) | 95.83 (6.28) | -1.33 | 145.90 | 0.19 |
| IQ. M (SD) | NA | NA | NA | NA | NA |
| **Adolescents** | | | | | |
| Age (years). M (SD) | 14.50 (2.68) | 13.99 (2.88) | 1.42 | 226.71 | 0.16 |
| Male (%). M (SD) | 82.69 (4.58) | 61.54 (14.16) | 16.02 | 161.47 | 3.42 |
| IQ. M (SD) | 94.76 (22.30) | NA | NA | NA | NA |
| **Adults** | | | | | |
| Age (years). M (SD) | 20.02 (5.89) | 19.77 (5.11) | 0.29 | 160.83 | 0.77 |
| Male (%). M (SD) | 86.76 (7.94) | 77.94 (18.23) | 3.66 | 91.53 | **0.00** |
| IQ. M (SD) | 99.28 (17.35) | NA | NA | NA | NA |

*Note:* k = number of studies, n = number of participants

## **sTable 8. Study Distribution and demographic characteristics displayed for N100 amplitude**

| **N100 amplitude** | | | | | |
| --- | --- | --- | --- | --- | --- |
|  | **k** | **Autistic n** | **Non-autistic n** | **Total n** | |
| Total | 26 | 629 | 693 | 1322 | |
| EEG | 26 | 629 | 693 | 1322 | |
| MEG | 0 | 0 | 0 | 0 | |
| Auditory | 13 | 376 | 424 | 800 | |
| Visual | 11 | 194 | 200 | 394 | |
| Tactile | 0 | 0 | 0 | 0 | |
| Combined modalities | 2 | 59 | 69 | 128 | |
| Children | 7 | 213 | 271 | 484 | |
| Adolescents | 10 | 260 | 284 | 544 | |
| Adults | 9 | 156 | 138 | 294 | |
|  | **Autistic** | **Non-autistic** | **t** | **df** | **p** |
| **Total** | | | | | |
| Age (years). M (SD) | 15.37 (6.48) | 14.99 (6.82) | 1.02 | 1.287 | 0.31 |
| Male (%). M (SD) | 80.24 (12.97) | 63.73 (16.58) | 19.58 | 1.216 | **<0.0001** |
| IQ. M (SD) | 100.34 (17.61) | 110.07 (11.16) | -8.93 | 685 | **<0.0001** |
| **Children** | | | | | |
| Age (years). M (SD) | 9.78 (2.75) | 10.00 (2.97) | -0.81 | 469.20 | 0.42 |
| Male (%). M (SD) | 87.77 (6.76) | 57.38 (14.86) | 27.92 | 348.00 | **<0.0001** |
| IQ. M (SD) | 102.15 (17.09) | 110.01 (11.17) | -5.26 | 301.66 | **<0.0001** |
| **Adolescents** | | | | | |
| Age (years). M (SD) | 14.96 (3.22) | 14.73 (3.43) | 0.78 | 541.66 | 0.44 |
| Male (%). M (SD) | 79.62 (7.54) | 65.84 (13.90) | 14.53 | 444.10 | **<0.0001** |
| IQ. M (SD) | 93.59 (19.24) | NA | NA | NA | NA |
| **Adults** | | | | | |
| Age (years). M (SD) | 25.19 (7.89) | 25.32 (8.91) | -0.13 | 266.41 | 0.90 |
| Male (%). M (SD) | 71.21 (19.89) | 70.29 (20.37) | 0.38 | 267.89 | 0.71 |
| IQ. M (SD) | 103.63 (15.19) | 110.19 (11.11) | -3.61 | 207.23 | **0.00** |

*Note:* k = number of studies, n = number of participants

## **sTable 9. Study Distribution and demographic characteristics displayed for N100 latency**

| **N100 latency** | | | | | | |
| --- | --- | --- | --- | --- | --- | --- |
|  | **k** | | **Autistic n** | **Non-autistic n** | **Total n** | |
| Total | 19 | | 369 | 402 | 771 | |
| EEG | 18 | | 347 | 371 | 718 | |
| MEG | 1 | | 22 | 31 | 53 | |
| Auditory | 10 | | 201 | 232 | 433 | |
| Visual | 9 | | 168 | 170 | 338 | |
| Tactile | 0 | | 0 | 0 | 0 | |
| Combined modalities | 0 | | 0 | 0 | 0 | |
| Children | 5 | | 75 | 72 | 147 | |
| Adolescents | 9 | | 226 | 261 | 487 | |
| Adults | 5 | | 68 | 69 | 137 | |
|  | | **Autistic** | **Non-autistic** | **t** | **df** | **p** |
| **Total** | | | | | | |
| Age (years). M (SD) | | 14.68 (4.9) | 13.80 (4.97) | 2.51 | 799.22 | **0.01** |
| Male (%). M (SD) | | 81.43 (9.78) | 70.56 (16.67) | 11.10 | 640.01 | **<0.0000** |
| IQ. M (SD) | | 98.36 (17.59) | 110.96 (15.25) | -4.12 | 46.35 | **0.00** |
| **Children** | | | | | | |
| Age (years). M (SD) | | 6.56 (2.82) | 4.40 (3.87) | 3.85 | 130 | **0.00** |
| Male (%). M (SD) | | 90.90 (6.44) | 87.23 (8.25) | 2.54 | 83 | **0.01** |
| IQ. M (SD) | | 110.00 (0.00) | NA | NA | NA | NA |
| **Adolescents** | | | | | | |
| Age (years). M (SD) | | 13.70 (3.36) | 13.14 (3.55) | 1.82 | 515.88 | 0.07 |
| Male (%). M (SD) | | 81.89 (8.53) | 70.86 (16.27) | 9.86 | 429.81 | **<0.0000** |
| IQ. M (SD) | | 93.11 (18.38) | 109.20 (18.55) | -3.33 | 21.30 | **0.00** |
| **Adults** | | | | | | |
| Age (years). M (SD) | | 27.14 (4.70) | 26.27 (5.29) | 1.01 | 133.61 | 0.31 |
| Male (%). M (SD) | | 70.59 (4.96) | 57.97 (11.15) | 8.58 | 94.17 | **<0.0000** |
| IQ. M (SD) | | 107.43 (13.77) | 113.10 (9.40) | -1.74 | 32.25 | 0.09 |

*Note:* k = number of studies, n = number of participants

## **sTable 10. Study Distribution and demographic characteristics displayed for N170 amplitude**

| **N170 amplitude** | | | | | |
| --- | --- | --- | --- | --- | --- |
|  | **k** | **Autistic n** | **Non-autistic n** | **Total n** | |
| Total | 28 | 732 | 670 | 1402 | |
| EEG | 28 | 732 | 670 | 1402 | |
| MEG | 0 | 0 | 0 | 0 | |
| Auditory | 0 | 0 | 0 | 0 | |
| Visual | 27 | 718 | 656 | 1374 | |
| Tactile | 0 | 0 | 0 | 0 | |
| Combined modalities | 1 | 14 | 14 | 28 | |
| Children | 11 | 299 | 260 | 559 | |
| Adolescents | 6 | 196 | 171 | 367 | |
| Adults | 11 | 237 | 239 | 476 | |
|  | **Autistic** | **Non-autistic** | **t** | **df** | **p** |
| **Total** | | | | | |
| Age (years). M (SD) | 15.50 (8.25) | 16.33 (8.62) | -1.82 | 1375.69 | 0.07 |
| Male (%). M (SD) | 86.48 (15.24) | 79.78 (18.11) | 5.39 | 693.76 | **<0.0001** |
| IQ. M (SD) | 100.22 (21.53) | 112.60 (14.38) | -8.27 | 535.44 | **<0.0001** |
| **Children** | | | | | |
| Age (years). M (SD) | 9.37 (3.81) | 9.33 (3.37) | 0.11 | 556.85 | 0.91 |
| Male (%). M (SD) | 90.53 (6.84) | 78.10 (16) | 8.49 | 176.16 | **<0.0001** |
| IQ. M (SD) | 93.52 (22.89) | 111.29 (15.19) | -8.32 | 303.02 | **<0.0001** |
| **Adolescents** | | | | | |
| Age (years). M (SD) | 14.68 (2.33) | 15.11 (2.29) | -1.78 | 359.80 | 0.08 |
| Male (%). M (SD) | 94.51 (6.34) | 89.66 (8.48) | 4.31 | 159.05 | **<0.0001** |
| IQ. M (SD) | 107.00 (14.46) | 112.23 (12.79) | -1.66 | 72.88 | 0.10 |
| **Adults** | | | | | |
| Age (years). M (SD) | 23.92 (7.20) | 24.80 (7.03) | -1.34 | 473.53 | 0.18 |
| Male (%). M (SD) | 73.63 (20.80) | 75 (21.95) | -0.50 | 236.01 | 0.62 |
| IQ. M (SD) | 109.91 (16.11) | 114.70 (13.39) | -2.24 | 179.32 | **0.03** |

*Note:* k = number of studies, n = number of participants

## **sTable 11. Study Distribution and demographic characteristics displayed for N170 latency**

| **N170 latency** | | | | | |
| --- | --- | --- | --- | --- | --- |
|  | **k** | **Autistic n** | **Non-autistic n** | **Total n** | |
| Total | 25 | 344 | 327 | 671 | |
| EEG | 25 | 344 | 327 | 671 | |
| MEG | 0 | 0 | 0 | 0 | |
| Auditory | 0 | 0 | 0 | 0 | |
| Visual | 24 | 330 | 313 | 643 | |
| Tactile | 0 | 0 | 0 | 0 | |
| Combined modalities | 1 | 14 | 14 | 28 | |
| Children | 11 | 171 | 141 | 312 | |
| Adolescents | 5 | 73 | 67 | 140 | |
| Adults | 9 | 100 | 119 | 219 | |
|  | **Autistic** | **Non-autistic** | **t** | **df** | **p** |
| **Total** | | | | | |
| Age (years). M (SD) | 15.23 (8.18) | 15.97 (8.60) | -1.61 | 1316.813 | 0.11 |
| Male (%). M (SD) | 85.46 (15.29) | 76.76 (18.02) | 6.73 | 640.12 | 3.76 |
| IQ. M (SD) | 98.76 (21.84) | 111.47 (14.18) | -7.83 | 457.44 | 3.52 |
| **Children** | | | | | |
| Age (years). M (SD) | 9.37 (3.85) | 9.35 (3.41) | 0.04 | 562.94 | 0.97 |
| Male (%). M (SD) | 90.64 (6.70) | 75.89 (16.66) | 9.88 | 177.22 | 1.31 |
| IQ. M (SD) | 92.74 (23.30) | 110.32 (15.12) | -7.83 | 277.60 | 1.01 |
| **Adolescents** | | | | | |
| Age (years). M (SD) | 14.54 (2.23) | 14.75 (2.04) | -1.25 | 325.13 | 0.21 |
| Male (%). M (SD) | 93.15 (6.39) | 86.57 (7.20) | 5.70 | 132.42 | 7.34 |
| IQ. M (SD) | 103.40 (8.90) | 106.9 (6.55) | -1.38 | 34.98 | 0.18 |
| **Adults** | | | | | |
| Age (years). M (SD) | 23.63 (7.15) | 24.52 (7.10) | -1.34 | 451.00 | 0.18 |
| Male (%). M (SD) | 70.99 (19.99) | 72.27 (21.41) | -0.46 | 214.58 | 0.65 |
| IQ. M (SD) | 109.27 (16.21) | 114.18 (13.13) | -2.16 | 157.70 | **0.03** |

*Note:* k = number of studies, n = number of participants

## **sTable 12. Study Distribution and demographic characteristics displayed for N200 amplitude**

| **N200 amplitude** | | | | | |
| --- | --- | --- | --- | --- | --- |
|  | **k** | **Autistic n** | **Non-autistic n** | **Total n** | |
| Total | 23 | 422 | 460 | 882 | |
| EEG | 23 | 422 | 460 | 882 | |
| MEG | 0 | 0 | 0 | 0 | |
| Auditory | 14 | 291 | 299 | 590 | |
| Visual | 7 | 105 | 134 | 239 | |
| Tactile | 0 | 0 | 0 | 0 | |
| Combined modalities | 2 | 26 | 27 | 53 | |
| Children | 15 | 287 | 316 | 603 | |
| Adolescents | 6 | 110 | 118 | 228 | |
| Adults | 2 | 25 | 26 | 51 | |
|  | **Autistic** | **Non-autistic** | **t** | **df** | **p** |
| **Total** | | | | | |
| Age (years). M (SD) | 10.92 (5.33) | 10.81 (4.95) | 0.32 | 858.18 | 0.75 |
| Male (%). M (SD) | 81.75 (11.66) | 72.17 (16.23) | 10.13 | 833.17 | **<0.0001** |
| IQ. M (SD) | 91.25 (26.76) | 102.48 (26.83) | -3.86 | 314.28 | **0.00** |
| **Children** | | | | | |
| Age (years). M (SD) | 8.43 (3,17) | 8.60 (2.82) | -0.69 | 574.84 | 0.49 |
| Male (%). M (SD) | 80.14 (11.50) | 73.10 (13.57) | 6.89 | 598.17 | **<0.0001** |
| IQ. M (SD) | 88.07 (27.09) | 100,10 (26.56) | -3.77 | 279.56 | **0.00** |
| **Adolescents** | | | | | |
| Age (years). M (SD) | 14.46 (2.97) | 13.90 (3.74) | 1.26 | 220.45 | 0.21 |
| Male (%). M (SD) | 83.64 (11.36) | 67.80 (20.37) | 7.32 | 185.88 | **<0.0001** |
| IQ. M (SD) | 96.33 (23.39) | NA | NA | NA | NA |
| **Adults** | | | | | |
| Age (years). M (SD) | 23.93 (5.11) | 23.63 (2.57) | 0.27 | 35.08 | 0.79 |
| Male (%). M (SD) | 92 (7.69) | 80.77 (19.23) | 2.76 | 33.05 | **0.01** |
| IQ. M (SD) | 105.03 (22.51) | 127.00 (14.4) | -3.65 | 34.24 | **0.00** |

*Note:* k = number of studies, n = number of participants

## **sTable 13. Study Distribution and demographic characteristics displayed for N200 latency**

| **N200 latency** | | | | | |
| --- | --- | --- | --- | --- | --- |
|  | **k** | **Autistic n** | **Non-autistic n** | **Total n** | |
| Total | 18 | 327 | 366 | 693 | |
| EEG | 18 | 327 | 366 | 693 | |
| MEG | 0 | 0 | 0 | 0 | |
| Auditory | 11 | 222 | 232 | 454 | |
| Visual | 7 | 105 | 134 | 239 | |
| Tactile | 0 | 0 | 0 | 0 | |
| Combined modalities | 0 | 0 | 0 | 0 | |
| Children | 13 | 255 | 284 | 539 | |
| Adolescents | 4 | 59 | 69 | 128 | |
| Adults | 1 | 13 | 13 | 26 | |
|  | **Autistic** | **Non-autistic** | **t** | **df** | **p** |
| **Total** | | | | | |
| Age (years). M (SD) | 9.96 (5.14) | 9.63 (4.71) | 0,92 | 659,19 | 0,36 |
| Male (%). M (SD) | 81.65 (11.04) | 71.13 (16.15) | 9,35 | 622,16 | **<0,0000** |
| IQ. M (SD) | 88.27 (26.89) | 98.34 (27.81) | -3,05 | 241,44 | **0,003** |
| **Children** | | | | | |
| Age (years). M (SD) | 8.26 (3.28) | 8.01 (2.93) | 0,93 | 512,36 | 0,35 |
| Male (%). M (SD) | 80 (11.11) | 72.86 (14.65) | 6,30 | 498,37 | **<0,0000** |
| IQ. M (SD) | 86.61 (27.88) | 98.34 (27.81) | -3,31 | 242,10 | **0,001** |
| **Adolescents** | | | | | |
| Age (years). M (SD) | 13.73 (3.06) | 13.46 (4.23) | 0,41 | 122,77 | 0,68 |
| Male (%). M (SD) | 88.14 (9.29) | 69.57 (21.31) | 6,55 | 96,00 | **<0,0000** |
| IQ. M (SD) | 96.33 (23.39) | NA | NA | NA | NA |
| **Adults** | | | | | |
| Age (years). M (SD) | 26.17 (5) | 24.25 (2) | 1,29 | 15,74 | 0,22 |
| Male (%). M (SD) | 84.62 (0) | 61.54 (0) | NA | NA | NA |
| IQ. M (SD) | 89 (19) | NA | NA | NA | NA |

*Note:* k = number of studies, n = number of participants

## **sTable 14. Study Distribution and demographic characteristics displayed for MMN/MMF amplitude**

| **MMN/MMF amplitude** | | | | | |
| --- | --- | --- | --- | --- | --- |
|  | **k** | **Autistic n** | **Non-autistic n** | **Total n** | |
| Total | 39 | 871 | 744 | 1615 | |
| EEG | 36 | 754 | 674 | 1428 | |
| MEG | 3 | 117 | 70 | 187 | |
| Auditory | 36 | 798 | 664 | 1462 | |
| Visual | 2 | 50 | 57 | 107 | |
| Tactile | 0 | 0 | 0 | 0 | |
| Combined modalities | 1 | 23 | 23 | 46 | |
| Children | 23 | 506 | 432 | 938 | |
| Adolescents | 5 | 157 | 84 | 241 | |
| Adults | 11 | 208 | 228 | 436 | |
|  | **Autistic** | **Non-autistic** | **t** | **df** | **p** |
| **Total** | | | | | |
| Age (years). M (SD) | 13.77 (8.13) | 15.02 (8.49) | -3.01 | 1548.09 | **0.00** |
| Male (%). M (SD) | 82.46 (13.87) | 73.11 (19.68) | 10.74 | 1279.65 | **<0.0001** |
| IQ. M (SD) | 95.74 (25.23) | 113.33 (13.42) | -12.66 | 752.83 | **<0.0001** |
| **Children** | | | | | |
| Age (years). M (SD) | 9.32 (2.49) | 9.57 (2.44) | -1.56 | 918.51 | 0.12 |
| Male (%). M (SD) | 86.53 (8.71) | 77.94 (15.80) | 9.90 | 623.44 | **<0.0001** |
| IQ. M (SD) | 88.72 (26.62) | 113.06 (14.25) | -12.91 | 467.86 | **<0.0001** |
| **Adolescents** | | | | | |
| Age (years). M (SD) | 12.40 (3.48) | 13.03 (2.63) | 1.38 | 174.51 | 0.17 |
| Male (%). M (SD) | 84.71 (6.92) | 65.48 (23.30) | 7.39 | 90.90 | **<0.0001** |
| IQ. M (SD) | 103.69 (14.95) | 115.08 (10.76) | -2.23 | 21.80 | **0.04** |
| **Adults** | | | | | |
| Age (years). M (SD) | 26.87 (8.17) | 27.16 (7.38) | -0.38 | 418.54 | 0.70 |
| Male (%). M (SD) | 71.15 (20.14) | 67.11 (21.95) | 2.01 | 433.98 | **0.05** |
| IQ. M (SD) | 108.76 (16.07) | 113.51 (12.51) | -2.80 | 282.02 | **0.01** |

*Note:* k = number of studies, n = number of participants

## **sTable 15. Study Distribution and demographic characteristics displayed for MMN/MMF latency**

| **MMN/MMF latency** | | | | | |
| --- | --- | --- | --- | --- | --- |
|  | **k** | **Autistic n** | **Non-autistic n** | **Total n** | |
| Total | 31 | 693 | 580 | 1273 | |
| EEG | 26 | 560 | 482 | 1042 | |
| MEG | 5 | 133 | 98 | 231 | |
| Auditory | 29 | 643 | 523 | 1166 | |
| Visual | 2 | 50 | 57 | 107 | |
| Tactile | 0 | 0 | 0 | 0 | |
| Combined modalities | 0 | 0 | 0 | 0 | |
| Children | 21 | 439 | 373 | 812 | |
| Adolescents | 2 | 112 | 41 | 153 | |
| Adults | 8 | 142 | 166 | 308 | |
|  | **Autistic** | **Non-autistic** | **t** | **df** | **p** |
| **Total** | | | | | |
| Age (years). M (SD) | 12.97 (6.97) | 14.53 | -3.70 | 1.161.77 | **0.00** |
| Male (%). M (SD) | 84.05 (11.61) | 76.46 (16.80) | 9.07 | 974.36 | **<0.0000** |
| IQ. M (SD) | 92.98 (27.45) | 111.90 (13.19) | -11.12 | 547.81 | **<0.0000** |
| **Children** | | | | | |
| Age (years). M (SD) | 9.21 (2.53) | 9.38 (2.30) | -0.99 | 806.23 | 0.32 |
| Male (%). M (SD) | 86.76 (8.02) | 77.37 (15.14) | 10.54 | 521.57 | **<0.0000** |
| IQ. M (SD) | 86.18 (28.38) | 109.66 (13.77) | -9.87 | 305.75 | **<0.0000** |
| **Adolescents** | | | | | |
| Age (years). M (SD) | 12.29 (3.71) | 13.01 (2.85) | -1.27 | 92.02 | 0.21 |
| Male (%). M (SD) | 82.14 (5.59) | 75.61 (13.85) | 2.93 | 44.86 | **0.01** |
| IQ. M (SD) | NA | NA | NA | NA | NA |
| **Adults** | | | | | |
| Age (years). M (SD) | 25.12 (6.97) | 26.49 (6.51) | -1.77 | 291.34 | 0.08 |
| Male (%). M (SD) | 77.47 (18.94) | 74.33 (20.33) | 1.23 | 303.76 | 0.22 |
| IQ. M (SD) | 104.43 (20.92) | 113.51 (12.51) | -4.28 | 216.89 | <**0.0000** |

*Note:* k = number of studies, n = number of participants

## **Forest plots**

### **sFigure 1. Forest Plot from amplitude differences in P/M50 component between groups.**


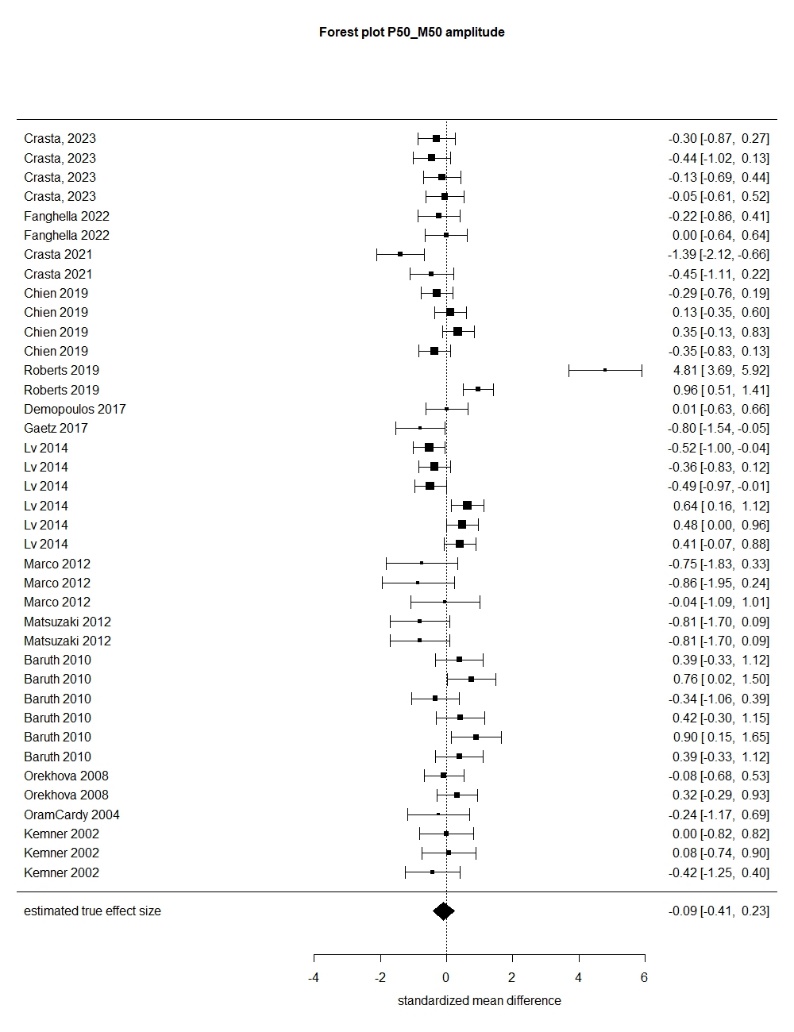


### **sFigure 2. Forest Plot from latency differences in P/M50 component between groups.**


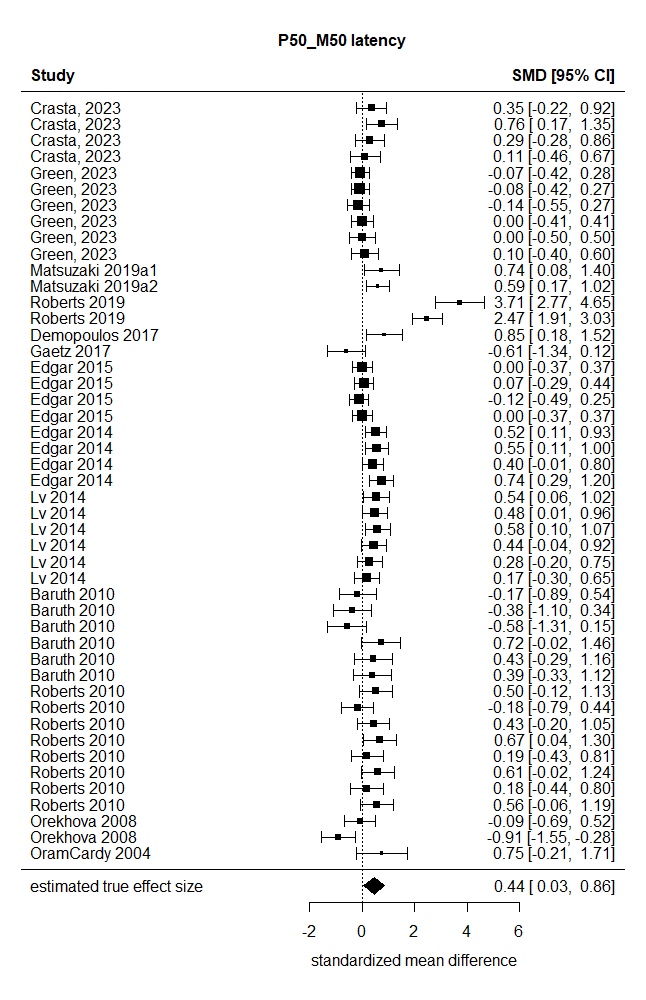


### **sFigure 3. Forest Plot from amplitude differences in P/M100 component between groups.**


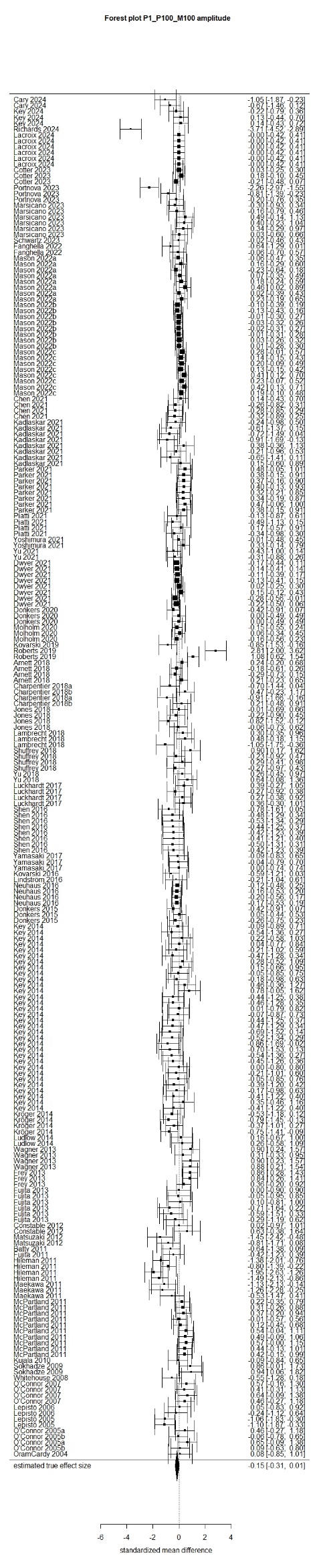

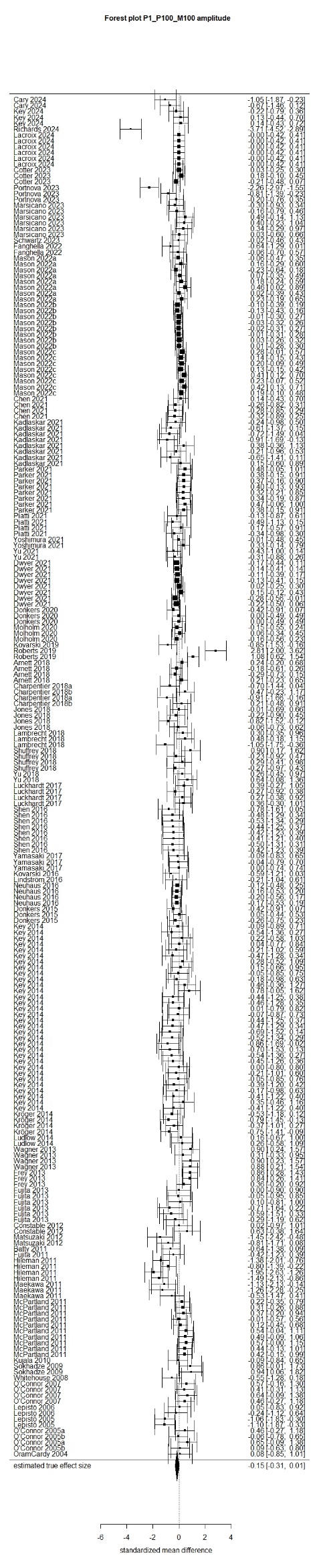


### **sFigure 4. Forest Plot from latency differences in P/M100 component between groups.**

**
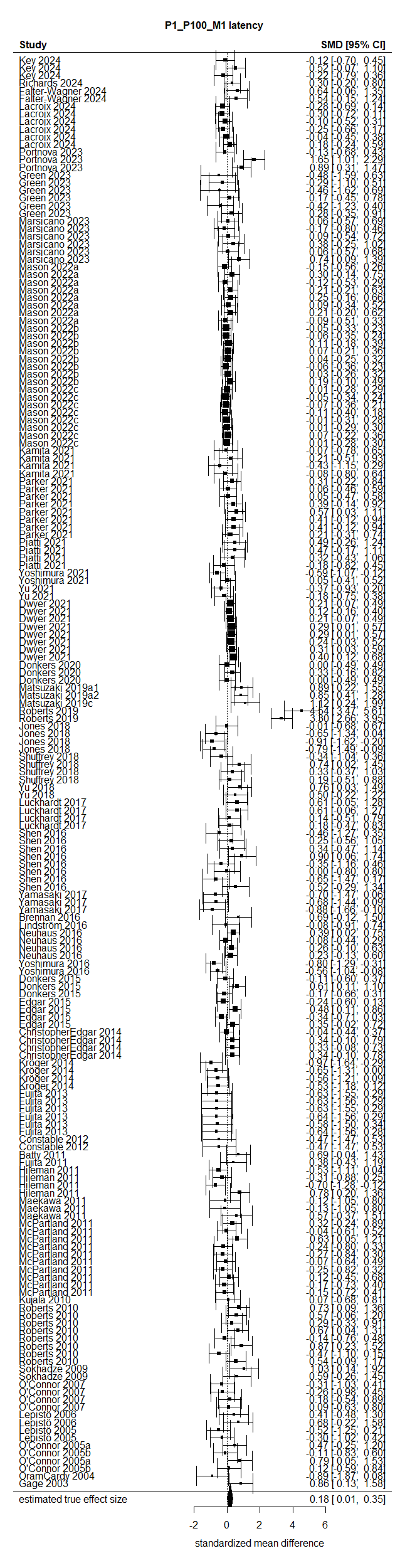

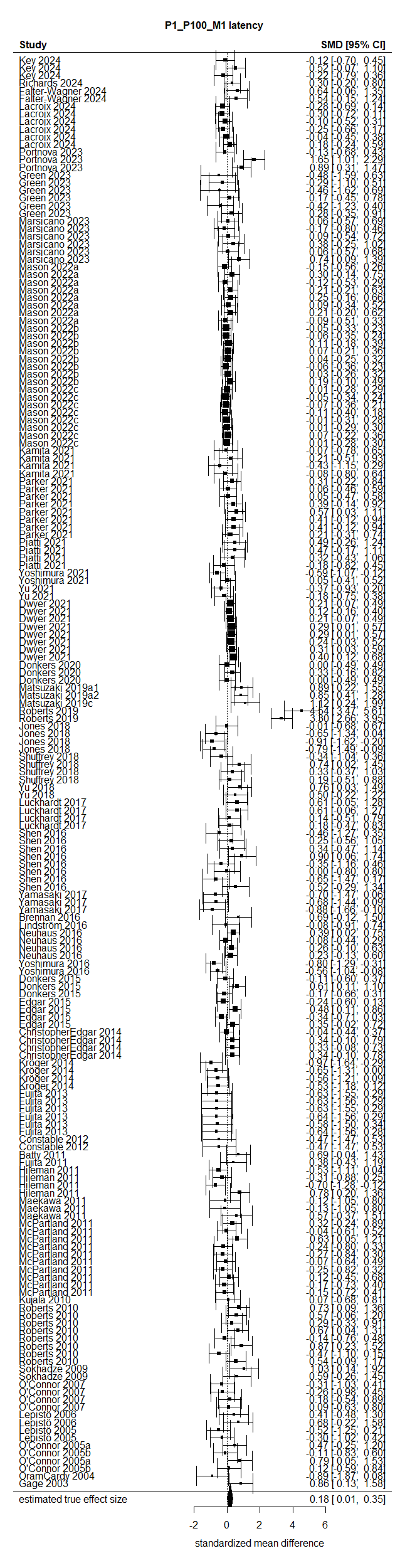
**

### **sFigure 5. Forest Plot from amplitude differences in P/M200 component between groups.**


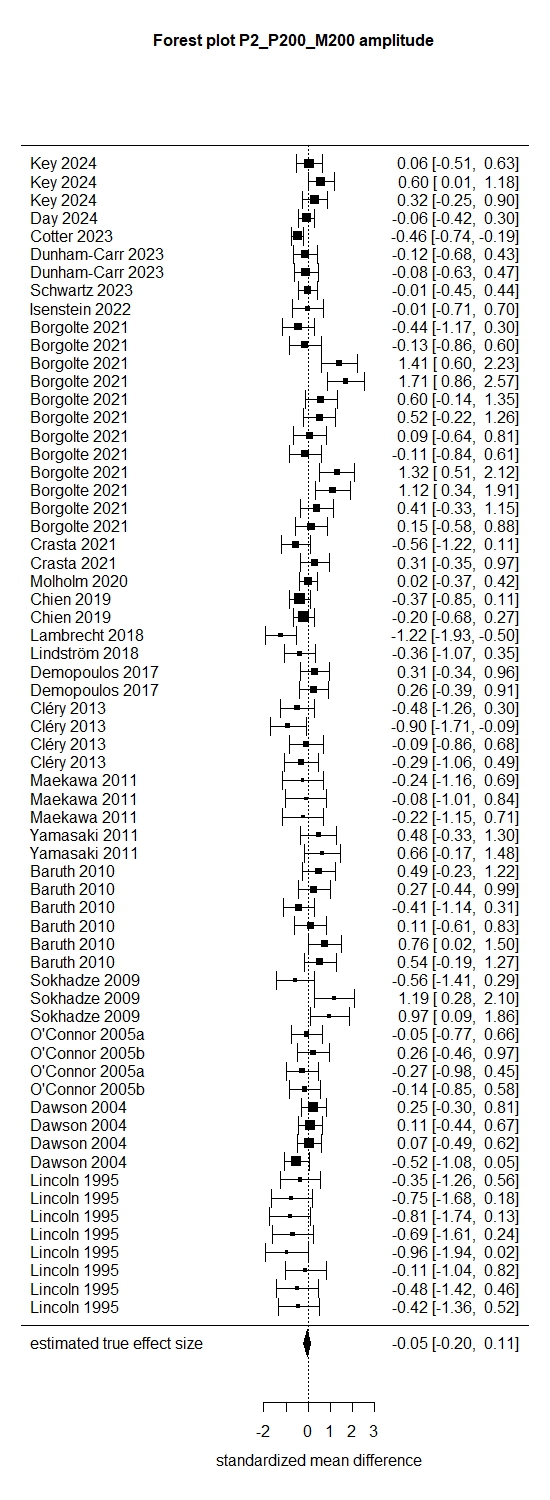


### **sFigure 6. Forest Plot from latency differences in P/M200 component between groups.**


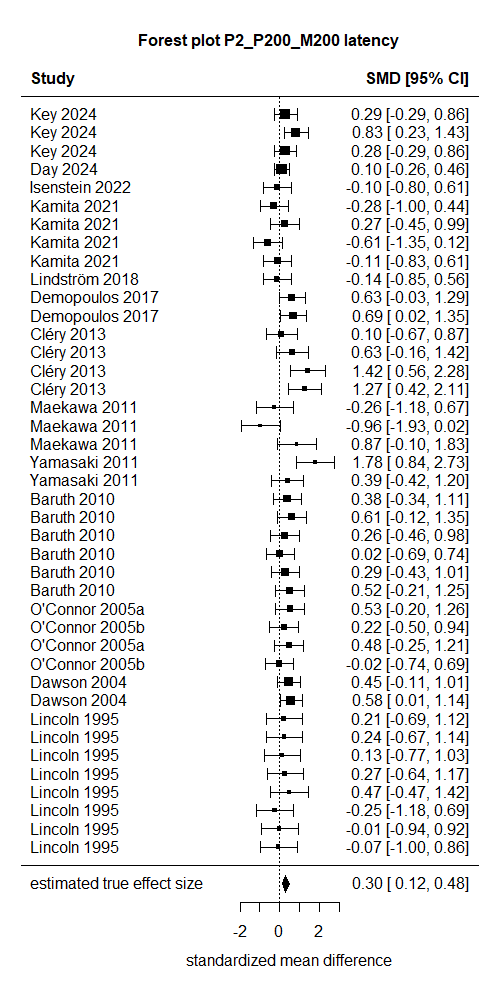


### **sFigure 7. Forest Plot from amplitude differences in N100 component between groups.**


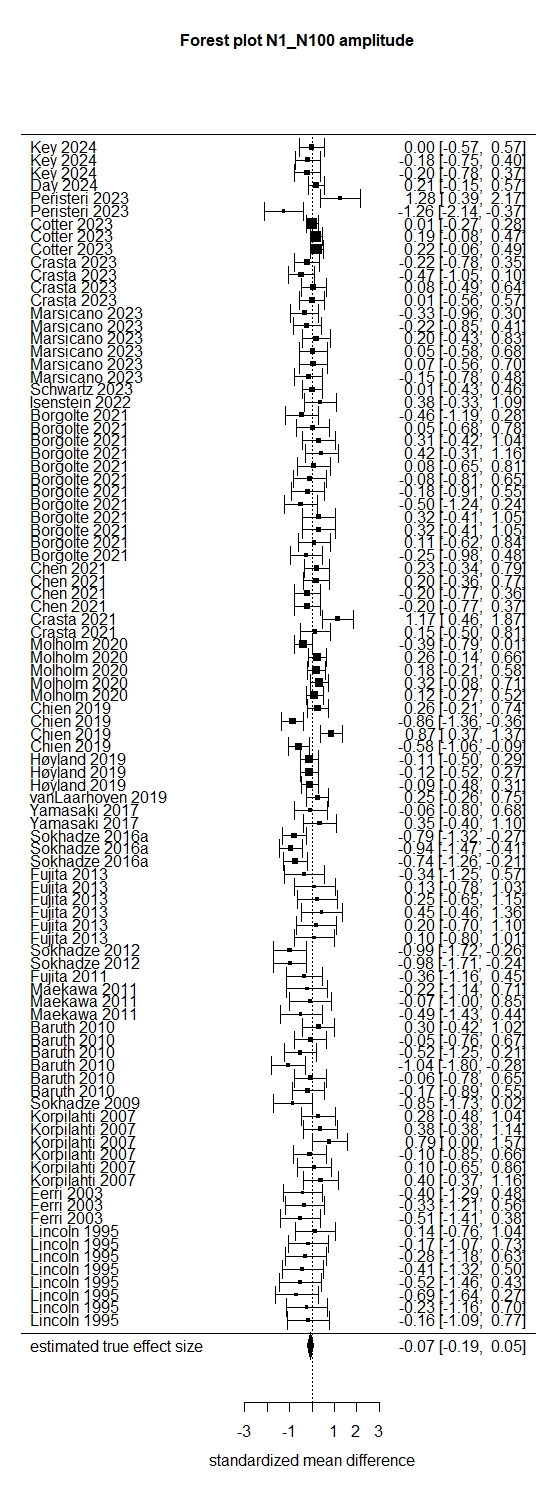


### **sFigure 8. Forest Plot from latency differences in N100 component between groups.**

**
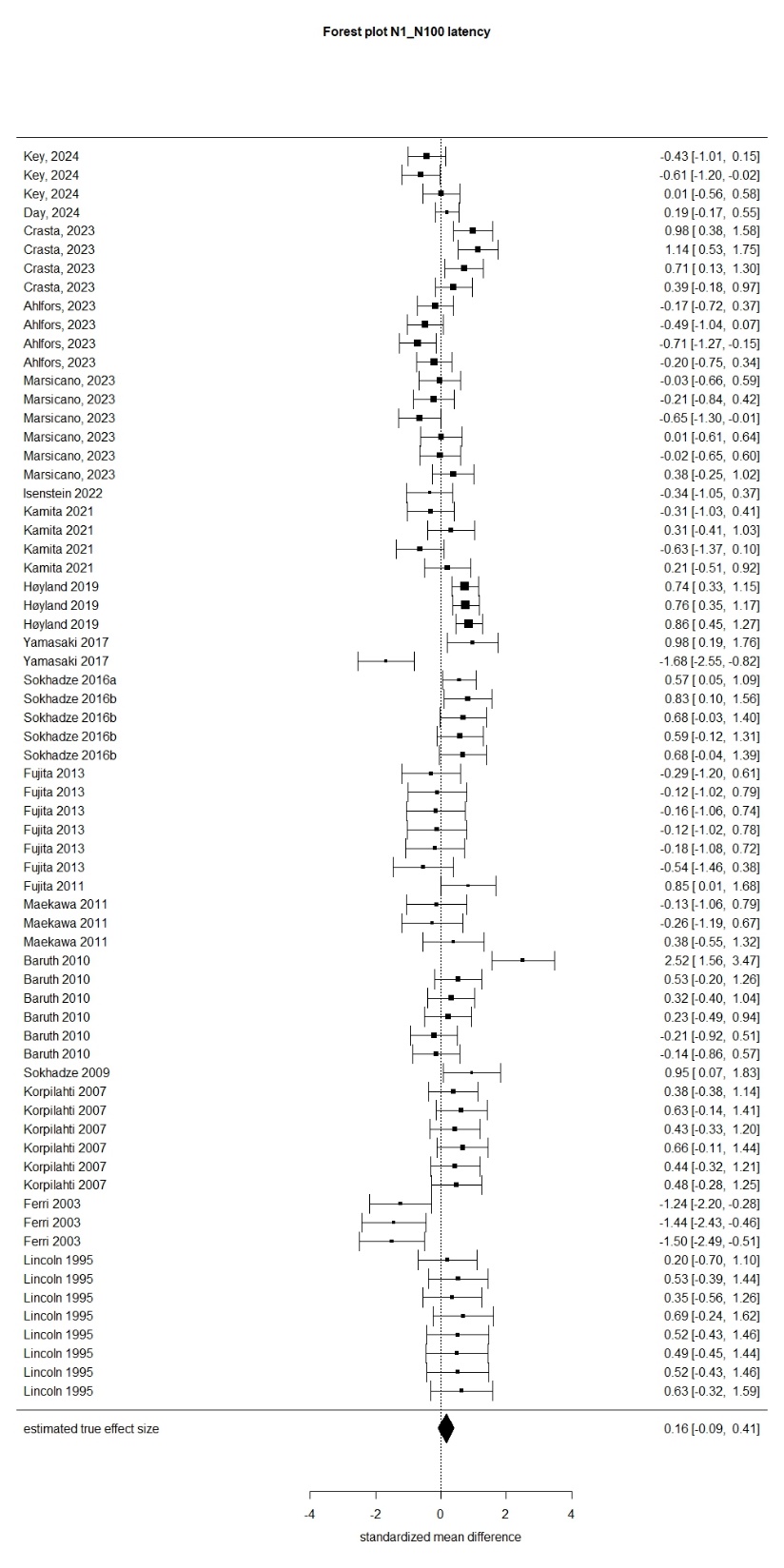
**

### **sFigure 9. Forest Plot from amplitude differences in N170 component between groups.**


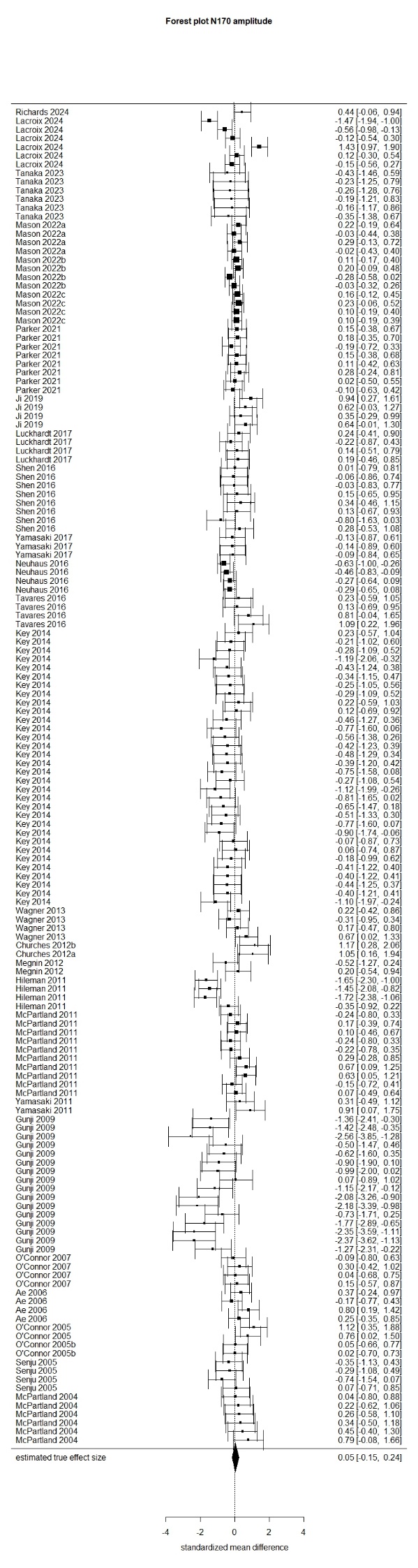


### **sFigure 10. Forest Plot from latency differences in N170 component between groups.**

### **
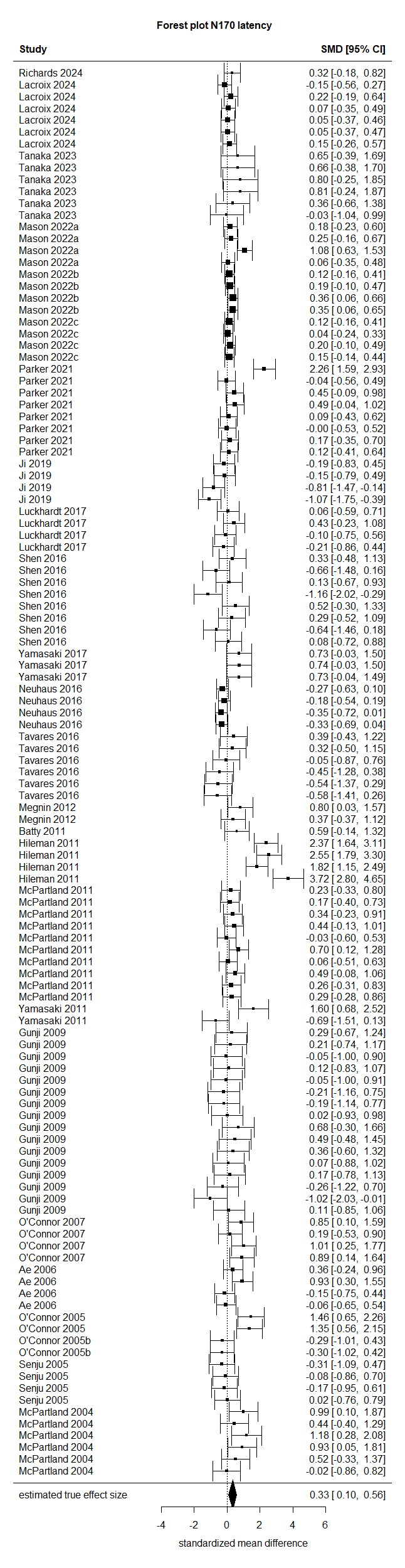
**

### **sFigure 11. Forest Plot from amplitude differences in N200 component between groups.**


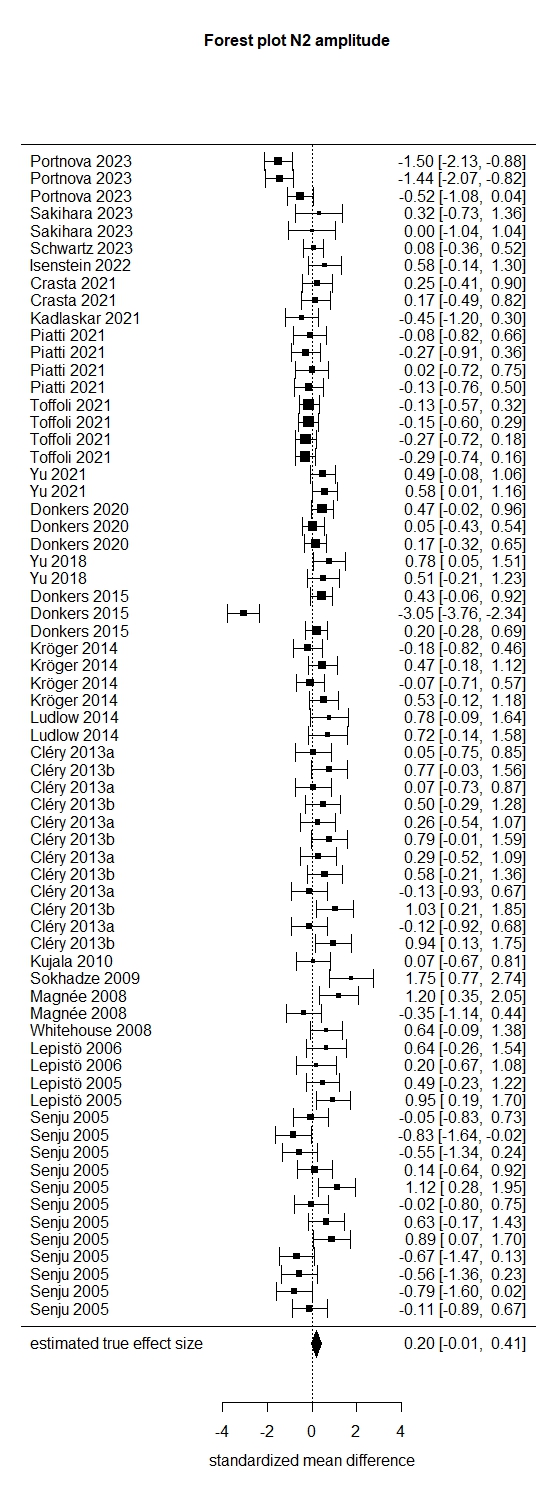


### **sFigure 12. Forest Plot from latency differences in N200 component between groups.**


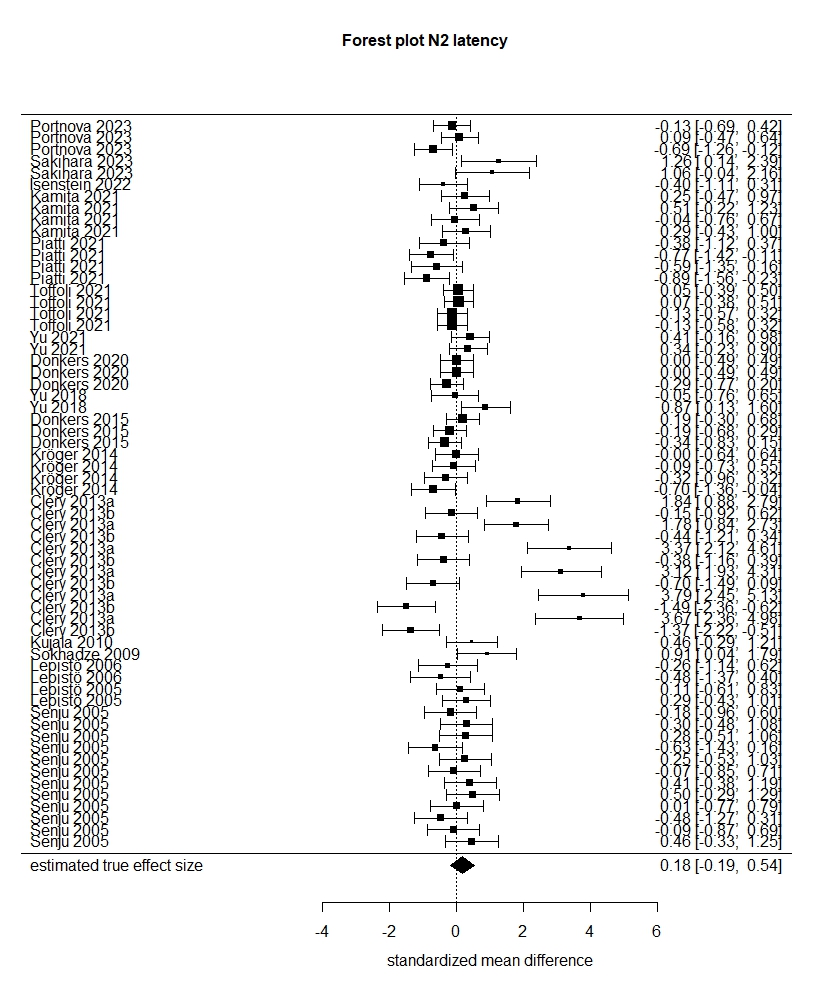


### **
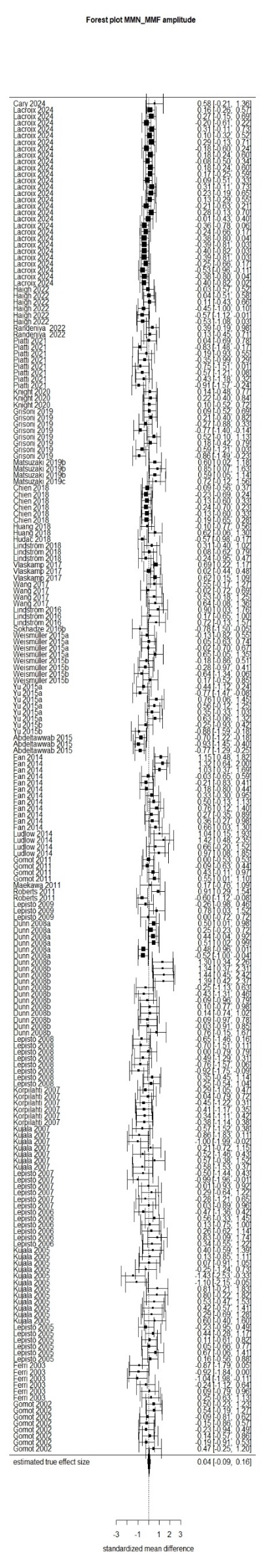

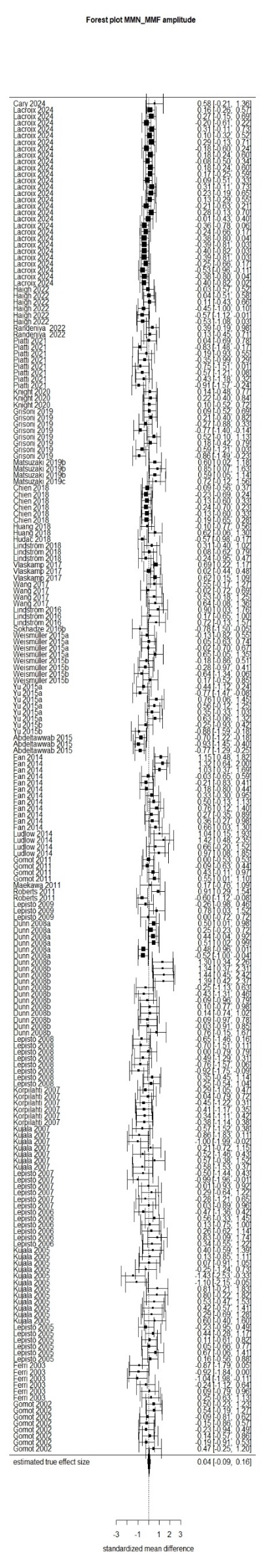
sFigure 13. Forest Plot from amplitude differences in MMN/MMF component between groups.**

### **sFigure 14. Forest Plot from latency differences in MMN/MMF component between groups.**


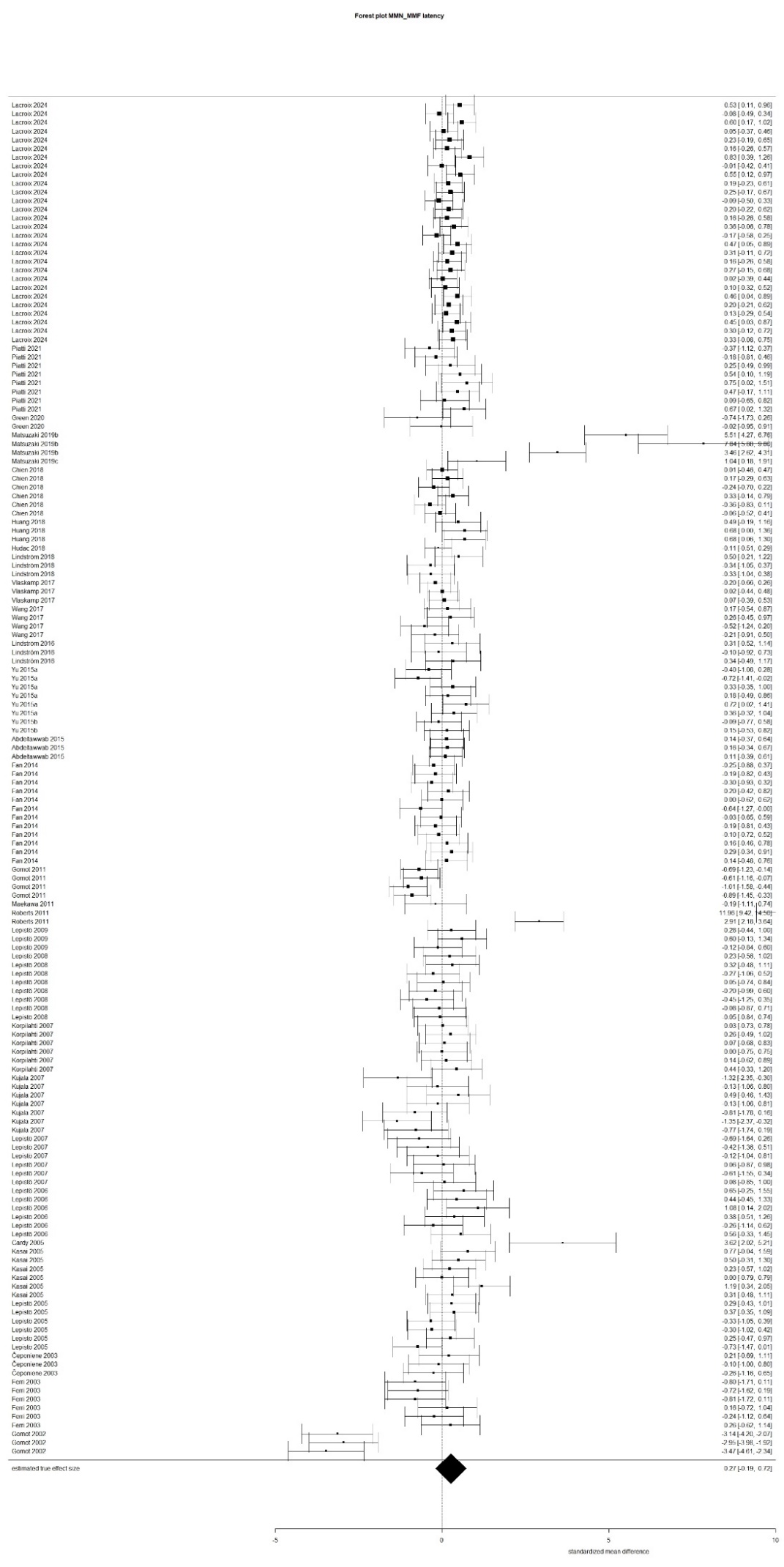


## **Moderator analyses**

### **sTable 16. Summary of significant and non-significant meta-analytic findings for amplitude moderator analyses (mixed-effects models fitted).**

| **Amplitude** | **k** | **Q_B_(df, p)** | **Q_W_(df, p)** | **SMD (p)** |
| --- | --- | --- | --- | --- |
| **P/M50** | | | | |
| EEG/MEG | NA | NA | NA | NA |
| Modality | NS | NS | NS | NA |
| Age | NS | NS | NS | NA |
| Language impairment | 14 | **29.09 (2, <.0001)** | 144.08 (37, <.0001) | NA |
| *Yes* | 2 | NA | NA | **2.45 (<.0001)** |
| *No* | 12 | NA | NA | -0.29 (0.20) |
| Task | NA | NA | NA | NA |
| Year of publication | NS | NS | NS | NA |
| Co-occurring condition | NA | NA | NA | NA |
| Medication | **16** | **33.60 (2, <.0001)** | 101.05 (14, <.0001) | NA |
| *Yes* | 4 | NA | NA | -1.47 (0.07) |
| ***No*** | 4 | NA | NA | **1.85 (0.03)** |
| Classification system | NS | NS | NS | NA |
| IQ | NS | NS | NS | NA |
| Sex | NS | NS | NS | NA |
| Quality rating | NS | NS | NS | NA |
| **P/M100** | | | | |
| EEG/MEG | NS | NS | NS | NA |
| Modality | NS | NS | NS | NA |
| Age | NS | NS | NS | NA |
| Language impairment | NS | NS | NS | NA |
| Task | NS | NS | NS | NA |
| Year of publication | NS | NS | NS | NA |
| Co-occurring condition | NS | NS | NS | NA |
| Medication | NS | NS | NS | NA |
| Classification system | NS | NS | NS | NA |
| IQ | NS | NS | NS | NA |
| Sex | NS | NS | NS | NA |
| Quality rating | NS | NS | NS | NA |
| **N100** | | | | |
| EEG/MEG | NA | NA | NA | NA |
| Modality | 93 | **8.54 (3, 0.04)** | 144.42 (90, 0.0002) | NA |
| *Visual* | 35 | NA | NA | -**0.27 (0.005)** |
| *Auditory* | 41 | NA | NA | 0.05 (0.57) |
| *Combined* | 17 | NA | NA | 0.06 (0.74) |
| Age | 93 | **9.08 (3, 0.03)** | 142.19 (90, 0.0004) | NA |
| *Children* | 34 | NA | NA | 0.09 (0.33) |
| *Adolescences* | 24 | NA | NA | **-0.27 (0.004)** |
| *Adults* | 35 | NA | NA | -0.02 (0.84) |
| Language impairment | NS | NS | NS | NA |
| Task | NS | NS | NS | NA |
| Year of publication | NS | NS | NS | NA |
| Co-occurring condition | NS | NS | NS | NA |
| **Amplitude** | **k** | **Q_B_(df ,p)** | **Q_W_(df ,p)** | **SMD (p)** |
| **N100** | | | | |
| Medication | NS | NS | NS | NA |
| Classification system | NS | NS | NS | NA |
| IQ | NS | NS | NS | NA |
| Sex | NS | NS | NS | NA |
| Quality rating | NS | NS | NS | NA |
| **N170** | | | | |
| EEG/MEG | NA | NA | NA | NA |
| Modality | NS | NS | NS | NA |
| Age | NS | NS | NS | NA |
| Language impairment | NS | NS | NS | NA |
| Task | 150 | 11.94 (5, 0.04) | 316.68 (145, <0.0001) | NA |
| *Target detection* | 99 | NA | NA | -0.13 (0.24) |
| *Oddball paradigm* | 6 | NA | NA | -0.13 (0.73) |
| *Passive task* | 22 | NA | NA | 0.19 (0.21) |
| *Discrimination task* | 9 | NA | NA | **0.68 (0.01)** |
| *Face recognition task* | 14 | NA | NA | 0.32 (0.18) |
| Year of publication | NS | NS | NS | NA |
| Co-occurring condition | NS | NS | NS | NA |
| Medication | NS | NS | NS | NA |
| Classification system | NS | NS | NS | NA |
| IQ | NS | NS | NS | NA |
| Sex | NS | NS | NS | NA |
| Condition type | NS | NS | NS | NA |
| Quality rating | NS | NS | NS | NA |
| **P/M200** | | | | |
| EEG/MEG | NS | NS | NS | NA |
| Modality | NS | NS | NS | NA |
| Age | NS | NS | NS | NA |
| Language impairment | NS | NS | NS | NA |
| Task | NS | NS | NS | NA |
| Year of publication | NS | NS | NS | NA |
| Co-occurring condition | NS | NS | NS | NA |
| Medication | 19 | 30.24 (2, <0.001) | 11.87 (17, 0.80)) | NA |
| *Yes* | 7 | NA | NA | 0.02 (0.90) |
| *No* | 12 | NA | NA | **-0.49 (<.0001)** |
| Classification system | NS | NS | NS | NA |
| IQ | NS | NS | NS | NA |
| Sex | NS | NS | NS | NA |
| Quality rating | NS | NS | NS | NA |
| **N200** | | | | |
| EEG/MEG | NA | NA | NA | NA |
| Modality | NS | NS | NS | NA |
| Age | 67 | 8.41 (3, 0.03) | 215.82 (64, <0.0001) | NA |
| *Children* | 40 | NA | NA | 0.05 (0.71) |
| *Adolescences* | 19 | NA | NA | **0.47 (0.02)** |
| *Adults* | 8 | NA | NA | 0.59. (0.08) |
| Language impairment | NS | NS | NS | NA |
| **Amplitude** | **k** | **Q_B_(df, p)** | **Q_W_(df, p)** | **SMD (p)** |
| **N200** | | | | |
| Task | NS | NS | NS | NA |
| Year of publication | 67 | 16.25 (6, 0.01) | 198.5466 (61, <.0001) | NA |
| *2000-2012.DSM-IV.TR* | 21 | NA | NA | **0.47 (0.02)** |
| *2013-2023.DSM-V* | 46 | NA | NA | 0.10 (0.39) |
| Co-occurring condition | NS | NS | NS | NA |
| Medication | NA | NA | NA | NA |
| Classification system | 67 | 16.25 (6, 0.01) | 198.55 (61, <0.0001) | NA |
| *only DSM IV+DSM IV-TR (without ICD)* | 34 | NA | NA | **0.47 (0.002)** |
| *only DSM V (without ICD)* | 1 | NA | NA | -0.45 (0.40) |
| *only ICD 10 (without DSM)* | 8 | NA | NA | -0.34 (0.19) |
| *only ADOS/+ADIR (since 2013)* | 16 | NA | NA | -0.00 (0.97) |
| *DSM IV+DSM IV-TR+ICD 10* | 4 | NA | NA | 0.57 (0.09) |
| *More than one DSM criteria/not mentioned which DSM criteria* | 4 | NA | NA | 0.38 (0.22) |
| IQ | NS | NS | NS | NA |
| Sex | 67 | 6.59 (1, 0.01) | 211.10 (65, <0.0001) | **-1.30 (0.03)** |
| Quality rating | NS | NS | NS | NA |
| **MMN/MMF** | | | | |
| EEG/MEG | NS | NS | NS | NA |
| modality | NS | NS | NS | NA |
| age | NS | NS | NS | NA |
| language impairment | NS | NS | NS | NA |
| task | NS | NS | NS | NA |
| year of publication | NS | NS | NS | NA |
| co-occurring condition | NS | NS | NS | NA |
| medication | NS | NS | NS | NA |
| Classification system | NS | NS | NS | NA |
| IQ | NS | NS | NS | NA |
| Sex | NS | NS | NS | NA |
| Quality rating | 210 | 11.35 (1, 0.0008) | 433.23 (209, <0.0001) | 0.08 (0.0008) |

*Note.* k, number of included effect sizes; Q_B_ = Cochran’s Q statistic (between groups) for heterogeneity test. Qw = Cochran’s Q statistic (within groups) for heterogeneity test. NA, not available; NS, not significant.

### **sTable 17. Summary of significant and non-significant meta-analytic findings for latency moderator analyses (mixed-effects models fitted).**

| **Latency** | **k** | **Q_B_ (df ,p)** | **Qw (df, p)** | **SMD (p)** |
| --- | --- | --- | --- | --- |
| **P/M50** | | | | |
| EEG/MEG | NS | NS | NS | NA |
| modality | NA | NA | NA | NA |
| age | NS | NS | NS | NA |
| language impairment | 47 | 7.69 (2, 0.02) | 181.66 (45, <0.0001) | NA |
| *Yes* | 12 | NA | NA | **0.72 (0.006)** |
| *No* | 35 | NA | NA | 0.38 (0.07) |
| task | NA | NA | NA | NA |
| year of publication | NS | NS | NS | NA |
| co-occurring condition | NA | NA | NA | NA |
| medication | NS | NS | NS | NA |
| Classification system | NS | NS | NS | NA |
| IQ | NS | NS | NS | NA |
| Sex | NS | NS | NS | NA |
| Quality rating | NS | NS | NS | NA |
| **P/M100** | | | | |
| **EEG/MEG** | 195 | 10.40 (2, 0.01) | 513.85 (185, <0.0001) | NA |
| *EEG* | 151 | NA | NA | 0.07 (0.45) |
| *MEG* | 36 | NA | NA | **0.50 (0.001)** |
| **Modality** | 151 | 7.00 (2, 0.03) | 498.79 (185, <0.0001) | NA |
| *Visual* | 115 | NA | NA | 0.06 (0.63) |
| *Auditory* | 72 | NA | NA | **0.32 (0.007)** |
| age | NS | NS | NS | NA |
| language impairment | NS | NS | NS | NA |
| task | NS | NS | NS | NA |
| year of publication | NS | NS | NS | NA |
| co-occurring condition | 111 | 13.36 (3, 0.004) | 498.62 (184, <0.0001) | NA |
| *Yes* | 12 | NA | NA | 0.02 (0.95) |
| *No* | 99 | NA | NA | **0.40 (0.001)** |
| medication | NS | NS | NS | NA |
| Classification system | NS | NS | NS | NA |
| IQ | NS | NS | NS | NA |
| Sex | NS | NS | NS | NA |
| Quality rating | NS | NS | NS | NA |
| **N100** | | | | |
| EEG/MEG | NS | NS | NS | NA |
| modality | NS | NS | NS | NA |
| age | NS | NS | NS | NA |
| language impairment | NS | NS | NS | NA |
| task | NS | NS | NS | NA |
| year of publication | NS | NS | NS | NA |
| co-occurring condition | NS | NS | NS | NA |
| medication | NS | NS | NS | NA |
| Classification system | NS | NS | NS | NA |
| IQ | NS | NS | NS | NA |
| Sex | NS | NS | NS | NA |
| Quality rating | NS | NS | NS | NA |
| **N170** | | | | |
| EEG/MEG | NA | NA | NA | NA |
| Modality | NA | NA | NA | NA |
| **N170** | | | | |
| Age | 119 | 12.28 (3, 0.007) | 370.61 (116, <0.0001) | NA |
| *Children* | 62 | NA | NA | 0.08 (0.63) |
| *Adolescences* | 18 | NA | NA | **0.63 (0.01)** |
| *Adults* | 39 | NA | NA | **0.47 (0.01)** |
| Language impairment | 119 | 7.60 (2, 0.02) | 388.10 (117, <0.0001) | NA |
| *Yes* | 9 | NA | NA | 0.18 (0.68) |
| *No* | 110 | NA | NA | **0.34 (0.006)** |
| Task | 115 | 15.51 (5, <0.008) | 213.27 (110, <0.0001) | NA |
| *Target detection* | 63 | NA | NA | **0.23 (0.04)** |
| *Oddball paradigm* | 6 | NA | NA | 0.07 (0.84) |
| *Passive task* | 22 | NA | NA | **0.32 (0.02)** |
| *Discrimination task* | 10 | NA | NA | -0.34 (0.18) |
| *Face recognition task* | 4 | NA | NA | 0.42 (0.06) |
| Year of publication | 119 | 12.47 (2; 0.0020) | 358.4879 (117; <.0001) | NA |
| *2000-2012.DSM-IV.TR* | 57 | NA | NA | **0.57 (0.001)** |
| *2013-2023.DSM-V* | 62 | NA | NA | 0.13 (0.41) |
| Co-occurring condition | NA | NA | NA | NA |
| Medication | NS | NS | NS | NA |
| Classification system | NS | NS | NS | NA |
| Condition type | 118 | 10.52 (2, 0.01) | 384.2285 (116, <0.0001) | NA |
| *Objects* | 26 | NA | NA | 0.21 (0.15) |
| *Faces* | 92 | NA | NA | **0.36 (0.004)** |
| IQ | NS | NS | NS | NA |
| Sex | NS | NS | NS | NA |
| Quality rating | NS | NS | NS | NA |
| **P/M200** | | | | |
| EEG/MEG | NA | NA | NA | NA |
| Modality | 39 | 15.94 (2, 0.0003) | 46.57 (37, 0.13) | NA |
| *Visual* | 21 | NA | NA | **0.45 (<.0001)** |
| *Auditory* | 18 | NA | NA | 0.09 (0.45) |
| Age | 41 | 14.14 (3, 0.003) | 50.66 (38, 0.08) |  |
| *Children* | 19 | NA | NA | 0.19 (0.16) |
| *Adolescences* | 11 | NA | NA | 0.25 (0.11) |
| *Adults* | 11 | NA | NA | **0.55** (**0.002**) |
| Language impairment | 41 | 18.11 (2, 0.0001) | 48.97 (39, 0.13) |  |
| *Yes* | 12 | NA | NA | -0.02 (0.92) |
| *No* | 29 | NA | NA | **0.37 (<.0001)** |
| Task | 41 | 31.72 (5, <0.0001) | 40.66 (36, 0.27) |  |
| ***Target detection*** | 10 | NA | NA | **0.53 (0.001)** |
| *Oddball paradigm* | 12 | NA | NA | -0.09 (0.5384) |
| *Passive task* | 11 | NA | NA | **0.46** (**0.0003)** |
| *Discrimination task* | 4 | NA | NA | 0.29 (0.06) |
| *Face recognition task* | 4 | NA | NA | 0.30 (0.14) |
| Year of publication | 41 | 9.83 (3, 0.02) | 55.14 (38, 0.04) | NA |
| *1994-1999.DSM-IV* | 8 | NA | NA | 0.13 (0.70) |
| *2000-2012.DSM-IV.TR* | 17 | NA | NA | **0.38** (**0.01)** |
| *2013-2023.DSM-V* | 16 | NA | NA | 0.27 (0.06) |
| **P/M200** | | | | |
| Co-occurring condition | NA | NA | NA |  |
| Medication | NA | NA | NA |  |
| Classification system | 41 | 14.43 (4, 0.01) | 50.74 (37, 0.07) |  |
| *only DSM III+DSM III-TR (without ICD)* | 11 | NA | NA | 0.03 (0.89) |
| ***only DSM IV+DSM IV-TR (without ICD)*** | 24 | NA | NA | **0.44 (0.0003)** |
| *only ADOS/+ADIR (since 2013)* | 5 | NA | NA | 0.21 (0.29) |
| *More than one DSM criteria/not mentioned* | 1 | NA | NA | -0.14 (0.75) |
| IQ | NS | NS | NS | NA |
| Sex | NS | NS | NS | NA |
| Quality rating | NS | NS | NS | NA |
| **N200** | | | | |
| EEG/MEG | NA | NA | NA | NA |
| Modality | NS | NS | NS | NA |
| Age | NS | NS | NS | NA |
| Language impairment | NS | NS | NS | NA |
| Task | NS | NS | NS | NA |
| Year of publication | NS | NS | NS | NA |
| Co-occurring condition | NS | NS | NS | NA |
| Medication | NS | NS | NS | NA |
| Classification system | NS | NS | NS | NA |
| IQ | NS | NS | NS | NA |
| Sex | NS | NS | NS | NA |
| Quality rating | NS | NS | NS | NA |
| **MMN/MMF** | | | | |
| EEG/MEG | 156 | 37.37 (2, <0.0001) | 545.70 (154, <0.0001) | NA |
| *EEG* | 143 | NA | NA | -0.13 (0.43) |
| *MEG* | 13 | NA | NA | **2.54 (<.0001)** |
| Modality | NS | NS | NS | NA |
| Age | NS | NS | NS | NA |
| Language impairment | 150 | 8.20 (2, 0.02) | 673.53 (148, <0.0001) | NA |
| *Yes* | 36 | NA | NA | **0.67 (0.02)** |
| *No* | 114 | NA | NA | 0.13 (0.61) |
| Task | NA | NA | NA | NA |
| Year of publication | NS | NS | NS | NA |
| Co-occurring condition | NS | NS | NS | NA |
| Medication | NS | NS | NS | NA |
| Classification system | NS | NS | NS | NA |
| IQ | NS | NS | NS | NA |
| Sex | NS | NS | NS | NA |
| Quality rating | NS | NS | NS | NA |

*Note.* k, number of included effect sizes; Q_B_ = Cochran’s Q statistic (between groups) for heterogeneity test. Qw = Cochran’s Q statistic (within groups) for heterogeneity test. NA, not available; NS, not significant.

## **Sensitivity analyses**

### **sTable 18.Sensitivity analyses in amplitudes for each component**

|  | **k** | **Effect size** | **95% CI** | **Qw (df, p)** | **P-Value** |
| --- | --- | --- | --- | --- | --- |
| **P/M50** | | | | | |
| Outlier analysis | 38 | -0.12 | -0.37 0.14 | 97.92(37, <0.00) | 0.37 |
| **P/M100** | | | | | |
| Outlier analysis | 227 | -0.09 | -0.20 0.03 | 428.48 (226, < 0.00) | 0.14 |
| **P/M200** | | | | | |
| No Outliers | | | | | |
| **N100** | | | | | |
| No Outliers | | | | | |
| **N170** | | | | | |
| Outlier analysis | 151 | 0.05 | -0.12 0.23 | 378.39 (150, < 0.00) | 0.55 |
| **N200** | | | | | |
| Outlier analysis | 65 | 0.24 | 0.04. 0.44 | 164.8269 (64, 0.00) | 0.02 |
| **MMN/MMF** | | | | | |
| No Outliers | | | | | |

*Note:* k, number of included effect sizes; Qw = Cochran’s Q statistic (within groups) for heterogeneity test.

### **sTable 19.Sensitivity analyses in latencies for each component**

|  | **k** | **Effect size** | **95% CI** | **Qw (df, p)** | **P-Value** |
| --- | --- | --- | --- | --- | --- |
| **P/M50** | | | | | |
| Outlier analysis | 45 | 0.25 | 0.04 0.45 | 83.05 (44, 0.00) | 0.02 |
| **P/M100** | | | | | |
| Outlier analysis | 192 | 0.10 | -0.01 0.20 | 348.87 (191, 0.00) | 0.06 |
| **P/M200** | | | | | |
| No Outliers | | | | | |
| **N100** | | | | | |
| No Outliers | | | | | |
| **N170** | | | | | |
| Outlier analysis | 115 | 0.27 | 0.09 0.45 | 227.08 (114, 0.00) | 0.00 |
| **N200** | | | | | |
| Outlier analysis | 58 | 0.10 | -0.17 0.37 | 123.64 (57, < 0.00 | 0.48 |
| **MMN/MMF** | | | | | |
| No Outliers | | | | | |

*Note*: k, number of included effect sizes; Qw = Cochran’s Q statistic (within groups) for heterogeneity test.

## **Funnel Plots**

### **sFigure 15. Funnel Plot displaying meta-analytical results obtained from fitting multilevel models to P/M50 component amplitudes and latencies.**


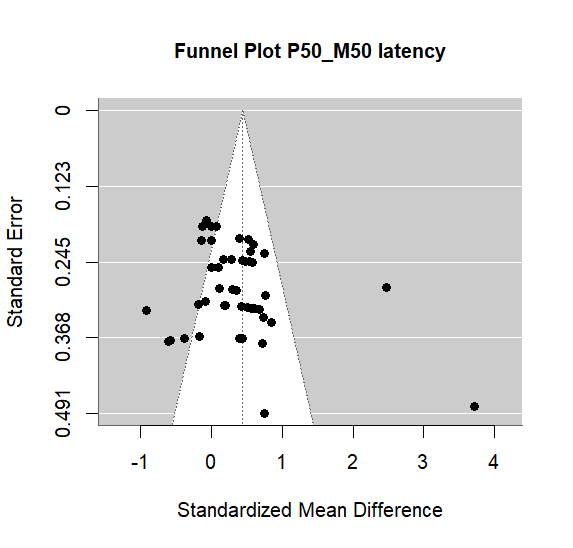

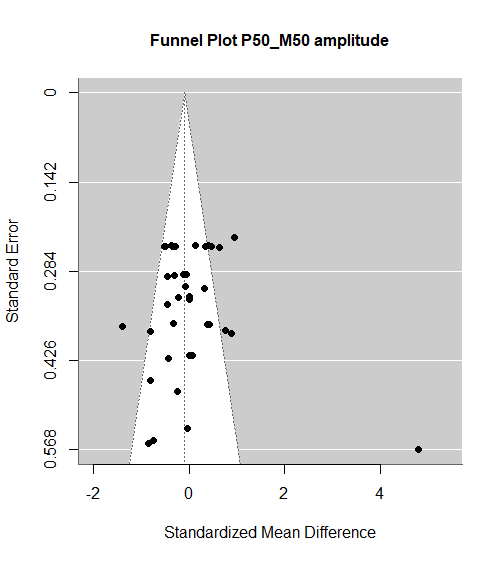


###


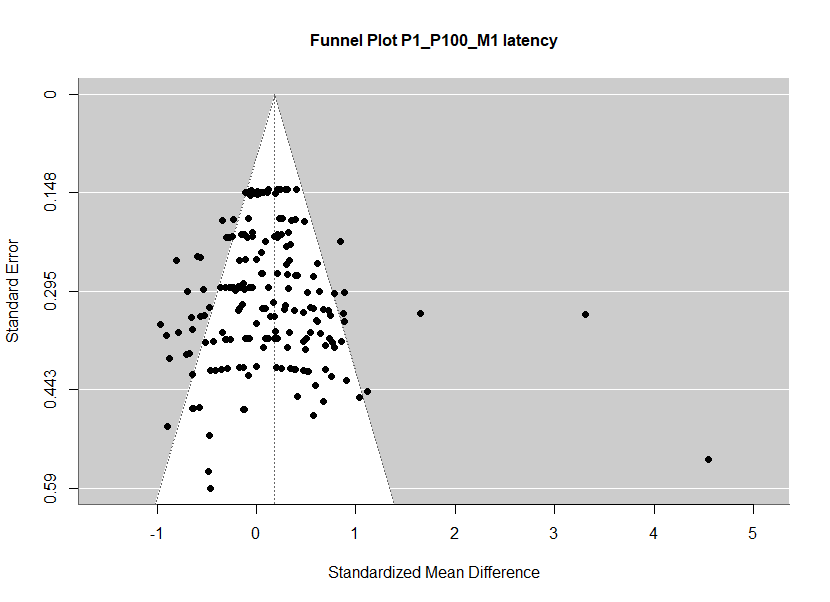

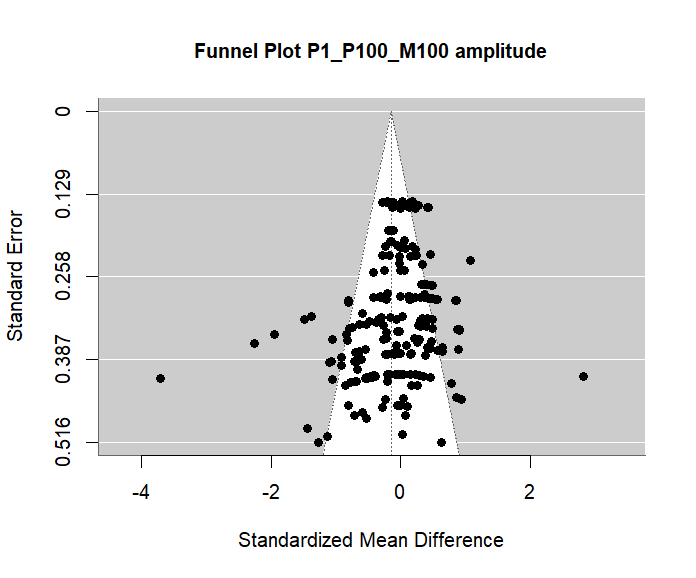


### **sFigure 16. Funnel Plot displaying meta-analytical results obtained from fitting multilevel models to P/M100 component amplitudes and latencies.**

### **sFigure 17. Funnel Plot displaying meta-analytical results obtained from fitting multilevel models to P/M200 component amplitudes and latencies.**


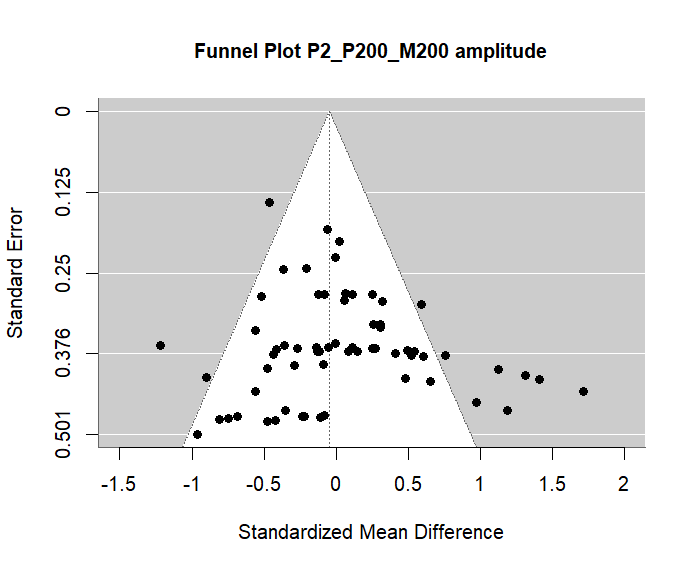

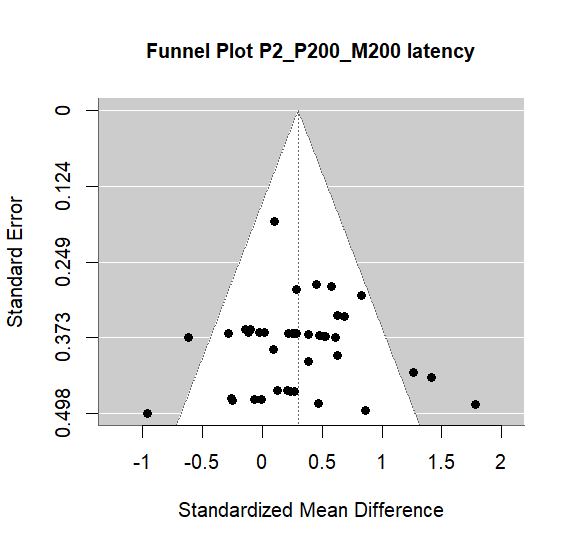


### **sFigure 18. Funnel Plot displaying meta-analytical results obtained from fitting multilevel models to N100 component amplitudes and latencies**


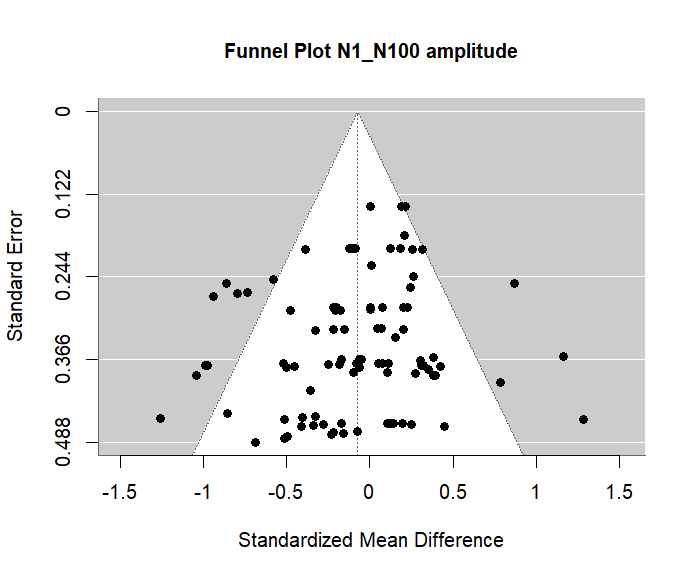

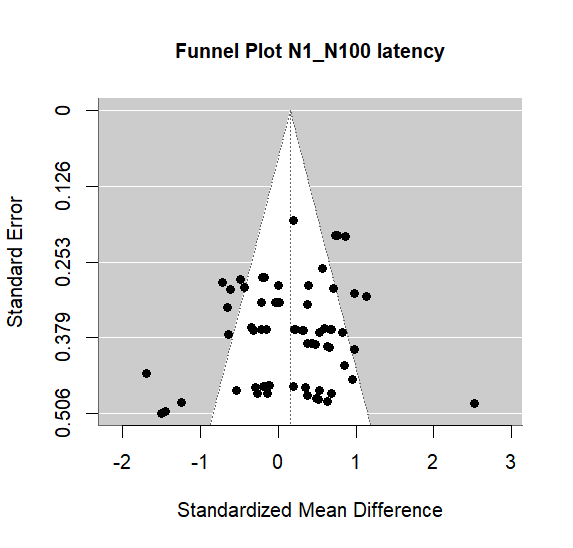


### **
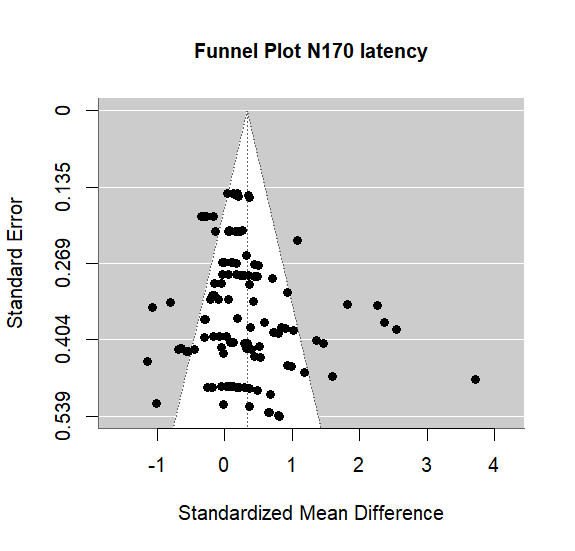
sFigure 19. Funnel Plot displaying meta-analytical results obtained from fitting multilevel models to N170 component amplitudes and latencies.**


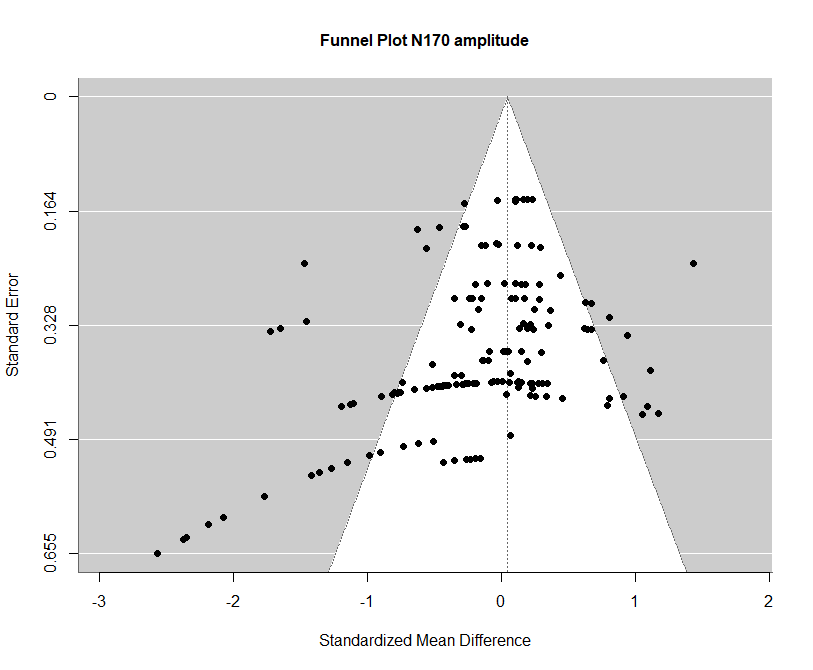


### **sFigure sFigure 20. Funnel Plot displaying meta-analytical results obtained from fitting multilevel models to N200 component amplitudes and latencies.**


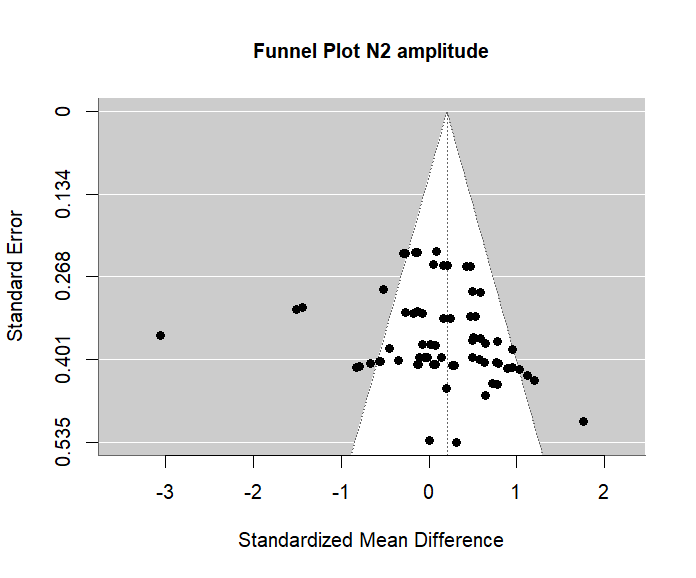

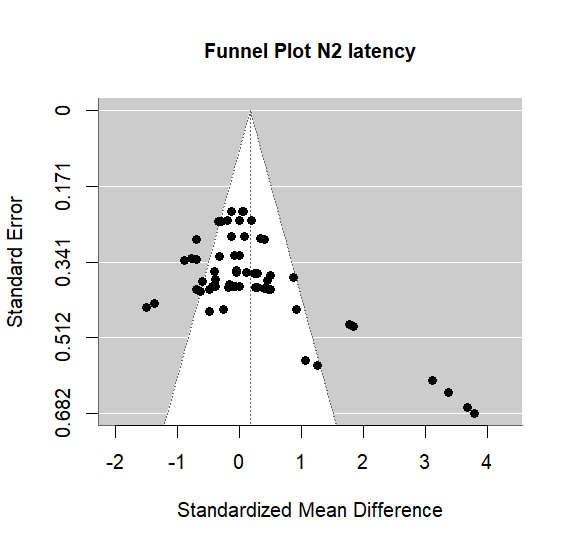


### **sFigure 21. Funnel Plot displaying meta-analytical results obtained from fitting multilevel models to MMN/MMF component amplitudes and latencies.**


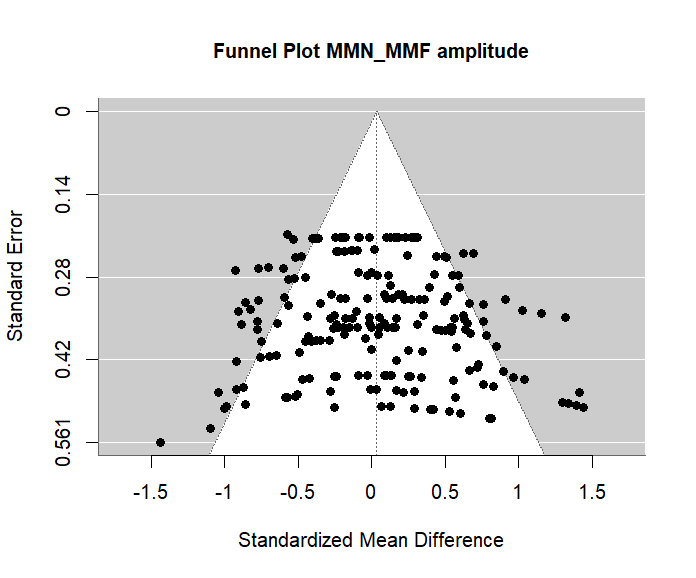

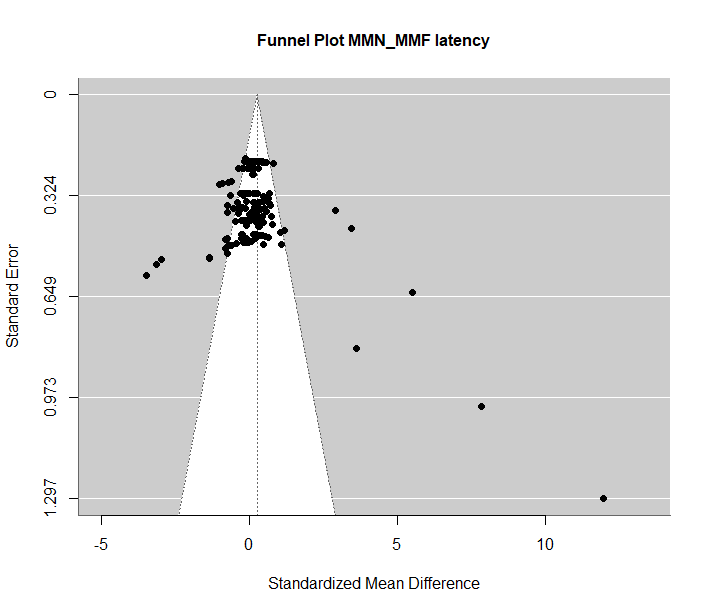


# **References**

1. Covidence systematic review software, Veritas Health Innovation. Melbourne, Australia. Available at www.covidence.org.
2. Wells GA, Shea B, O’Connell D, et al. The Newcastle-Ottawa Scale (NOS) for assessing the quality of nonrandomised studies in meta-analyses. Ottawa: Ottawa Hospital Research Institute; 2000. Available from: http://www.ohri.ca/programs/clinical_epidemiology/oxford.asp
3. Abdeltawwab MM, Baz H. Automatic Pre-Attentive Auditory Responses: MMN to Tone Burst Frequency Changes in Autistic School-Age Children. J Int Adv Otol. 2015;11(1):36-41. https://doi.org/10.5152/iao.2014.438.
4. Ahlfors SP, Graham S, Bharadwaj H, et al. No Differences in Auditory Steady-State Responses in Children with Autism Spectrum Disorder and Typically Developing Children. J Autism Dev Disord. 2024;54(5):1947-1960. https://doi.org/10.1007/s10803-023-05907-w.
5. Arnett AB, Hudac CM, DesChamps TD, et al. Auditory perception is associated with implicit language learning and receptive language ability in autism spectrum disorder. Brain Lang. 2018;187:1-8. https://doi.org/10.1016/j.bandl.2018.09.007.
6. Baruth JM, Casanova MF, Sears L, Sokhadze E. Early-stage visual processing abnormalities in high-functioning autism spectrum disorder (ASD). Transl Neurosci. 2010;1(2):177-187. https://doi.org/10.2478/v10134-010-0024-9.
7. Batty M, Meaux E, Wittemeyer K, Rogé B, Taylor MJ. Early processing of emotional faces in children with autism: An event-related potential study. J Exp Child Psychol. 2011;109(4):430-44. https://doi.org/10.1016/j.jecp.2011.02.001.
8. Borgolte A, Roy M, Sinke C, et al. Enhanced attentional processing during speech perception in adult high-functioning autism spectrum disorder: An ERP-study. Neuropsychologia. 2021;161:108022. https://doi.org/10.1016/j.neuropsychologia.2021.108022.
9. Brennan JR, Wagley N, Kovelman I, Bowyer SM, Richard AE, Lajiness-O'Neill R. Magnetoencephalography shows atypical sensitivity to linguistic sound sequences in autism spectrum disorder. Neuroreport. 2016;27(13):982-6. https://doi.org/10.1097/WNR.0000000000000643.
10. Bruneau N, Roux S, Adrien J,Barthélémy C. Auditory associative cortex dysfunction in children with autism: evidence from late auditory evoked potentials (N1 wave–T complex). Clin Neurophysiol. 1999;110:1927-1934.
11. Bruneau N, Bonnet-Brilhault F, Gomot M, Adrien JL, Barthélémy C. Cortical auditory processing and communication in children with autism: electrophysiological/behavioral relations. Int J Psychophysiol. 2003;51(1):17-25. https://doi.org/10.1016/s0167-8760(03)00149-1.
12. Oram Cardy JE, Flagg EJ, Roberts W, Roberts TP. Delayed mismatch field for speech and non-speech sounds in children with autism. Neuroreport. 2005;16(5):521-5. https://doi.org/10.1097/00001756-200504040-00021.
13. Cary E, Pacheco D, Kaplan-Kahn E, et al. Brain Signatures of Early and Late Neural Measures of Auditory Habituation and Discrimination in Autism and Their Relationship to Autistic Traits and Sensory Overresponsivity. J Autism Dev Disord. 2024;54(4):1344-1360. https://doi.org/10.1007/s10803-022-05866-8.
14. Ceponiene R, Lepistö T, Shestakova A, et al. Speech-sound-selective auditory impairment in children with autism: they can perceive but do not attend. Proc Natl Acad Sci U S A. 2003;100(9):5567-72. https://doi.org/10.1073/pnas.0835631100.
15. Charpentier J, Kovarski K, Houy-Durand E, Malvy J, Saby A, Bonnet-Brilhault F, Latinus M, Gomot M. Emotional prosodic change detection in autism Spectrum disorder: an electrophysiological investigation in children and adults. J Neurodev Disord. 2018;10(1):28. https://doi.org/10.1186/s11689-018-9246-9.
16. Chen F, Zhang H, Ding H, Wang S, Peng G, Zhang Y. Neural coding of formant-exaggerated speech and nonspeech in children with and without autism spectrum disorders. Autism Res. 2021;14(7):1357-1374. https://doi.org/10.1002/aur.2509.
17. Chien YL, Hsieh MH, Gau SS. Mismatch Negativity and P3a in Adolescents and Young Adults with Autism Spectrum Disorders: Behavioral Correlates and Clinical Implications. J Autism Dev Disord. 2018;48(5):1684-1697. https://doi.org/10.1007/s10803-017-3426-4.
18. Chien YL, Hsieh MH, Gau SS. P50-N100-P200 sensory gating deficits in adolescents and young adults with autism spectrum disorders. Prog Neuropsychopharmacol Biol Psychiatry. 2019;95:109683. https://doi.org/10.1016/j.pnpbp.2019.109683.
19. Edgar JC, Lanza MR, Daina AB, et al. Missing and delayed auditory responses in young and older children with autism spectrum disorders. Front Hum Neurosci. 2014;8:417. https://doi.org/10.3389/fnhum.2014.00417.
20. Churches O, Damiano C, Baron-Cohen S, Ring H. Getting to know you: the acquisition of new face representations in autism spectrum conditions. Neuroreport. 2012;23(11):668-72. https://doi.org/10.1097/WNR.0b013e3283556658.
21. Churches O, Baron-Cohen S, Ring H. The psychophysiology of narrower face processing in autism spectrum conditions. Neuroreport. 2012;23(6):395-9. https://doi.org/10.1097/WNR.0b013e3283525bc8.
22. Cléry H, Bonnet-Brilhault F, Lenoir P, Barthelemy C, Bruneau N, Gomot M. Atypical visual change processing in children with autism: an electrophysiological study. Psychophysiology. 2013;50(3):240-52. https://doi.org/10.1111/psyp.12006.
23. Cléry H, Roux S, Houy-Durand E, Bonnet-Brilhault F, Bruneau N, Gomot M. Electrophysiological evidence of atypical visual change detection in adults with autism. Front Hum Neurosci. 2013;7:62. https://doi.org/10.3389/fnhum.2013.00062.
24. Constable PA, Gaigg SB, Bowler DM, Thompson DA. Motion and pattern cortical potentials in adults with high-functioning autism spectrum disorder. Doc Ophthalmol. 2012;125(3):219-27. https://doi.org/10.1007/s10633-012-9349-7.
25. Cotter M, Reisli S, Francisco AA, et al. Neurophysiological measures of auditory sensory processing are associated with adaptive behavior in children with Autism Spectrum Disorder. J Neurodev Disord. 2023;15(1):11. https://doi.org/10.1186/s11689-023-09480-2.
26. Crasta JE, Gavin WJ, Davies PL. Expanding our understanding of sensory gating in children with autism spectrum disorders. Clin Neurophysiol. 2021;132(1):180-190. https://doi.org/10.1016/j.clinph.2020.09.020.
27. Crasta JE, Jacoby EC. The Effect of Attention on Auditory Processing in Adults on the Autism Spectrum. J Autism Dev Disord. 2024;54(9):3197-3210. https://doi.org/10.1007/s10803-023-06040-4.
28. Dawson G, Webb SJ, Carver L, Panagiotides H, McPartland J. Young children with autism show atypical brain responses to fearful versus neutral facial expressions of emotion. Dev Sci. 2004;7(3):340-59. https://doi.org/10.1111/j.1467-7687.2004.00352.x.
29. Day TC, Malik I, Boateng S, Hauschild KM, Lerner MD. Vocal Emotion Recognition in Autism: Behavioral Performance and Event-Related Potential (ERP) Response. J Autism Dev Disord. 2024;54(4):1235-1248. https://doi.org/10.1007/s10803-023-05898-8.
30. Demopoulos C, Hopkins J, Kopald BE, et al. Deficits in auditory processing contribute to impairments in vocal affect recognition in autism spectrum disorders: A MEG study. Neuropsychology. 2015;29(6):895-908. https://doi.org/10.1037/neu0000209.
31. Demopoulos C, Yu N, Tripp J, et al. Magnetoencephalographic Imaging of Auditory and Somatosensory Cortical Responses in Children with Autism and Sensory Processing Dysfunction. Front Hum Neurosci. 2017;11:259. https://doi.org/10.3389/fnhum.2017.00259.
32. Donkers FC, Schipul SE, Baranek GT, et al. Attenuated auditory event-related potentials and associations with atypical sensory response patterns in children with autism. J Autism Dev Disord. 2015;45(2):506-23. https://doi.org/10.1007/s10803-013-1948-y.
33. Donkers FC, Carlson M, Schipul SE, Belger A, Baranek GT. Auditory event-related potentials and associations with sensory patterns in children with autism spectrum disorder, developmental delay, and typical development. Autism. 2020;24(5):1093-1110. https://doi.org/10.1177/1362361319893196.
34. Dunham-Carr K, Feldman JI, Simon DM, et al. The Processing of Audiovisual Speech Is Linked with Vocabulary in Autistic and Nonautistic Children: An ERP Study. Brain Sci. 2023;13(7):1043. https://doi.org/10.3390/brainsci13071043.
35. Dunn MA, Gomes H, Gravel J. Mismatch negativity in children with autism and typical development. J Autism Dev Disord. 2008;38(1):52-71. https://doi.org/10.1007/s10803-007-0359-3.
36. Dwyer P, De Meo-Monteil R, Saron CD, Rivera SM. Effects of age on loudness-dependent auditory ERPs in young autistic and typically-developing children. Neuropsychologia. 2021;156:107837. https://doi.org/10.1016/j.neuropsychologia.2021.107837.
37. Edgar JC, Fisk Iv CL, Berman JI, et al. Auditory encoding abnormalities in children with autism spectrum disorder suggest delayed development of auditory cortex. Mol Autism. 2015;6:69. https://doi.org/10.1186/s13229-015-0065-5.
38. Falter CM, Braeutigam S, Nathan R, Carrington S, Bailey AJ. Enhanced access to early visual processing of perceptual simultaneity in autism spectrum disorders. J Autism Dev Disord. 2013;43(8):1857-66. https://doi.org/10.1007/s10803-012-1735-1.
39. Falter-Wagner CM, Kiefer CM, Bailey AJ, et al. Perceptual grouping in autism spectrum disorder: an exploratory magnetoencephalography study. J Autism Dev Disord. 2024;54(3):1101–12. https://doi.org/10.1007/s10803-022-05844-0.
40. Fan YT, Cheng Y. Atypical mismatch negativity in response to emotional voices in people with autism spectrum conditions. PLoS One. 2014;9(7):e102471. https://doi.org/10.1371/journal.pone.0102471.
41. Fanghella M, Gaigg SB, Candidi M, Forster B, Calvo-Merino B. Somatosensory Evoked Potentials Reveal Reduced Embodiment of Emotions in Autism. J Neurosci. 2022;42(11):2298-2312. https://doi.org/10.1523/JNEUROSCI.0706-21.2022.
42. Ferri R, Elia M, Agarwal N, Lanuzza B, Musumeci SA, Pennisi G. The mismatch negativity and the P3a components of the auditory event-related potentials in autistic low-functioning subjects. Clin Neurophysiol. 2003;114(9):1671-80. https://doi.org/10.1016/s1388-2457(03)00153-6.
43. Frey HP, Molholm S, Lalor EC, Russo NN, Foxe JJ. Atypical cortical representation of peripheral visual space in children with an autism spectrum disorder. Eur J Neurosci. 2013;38(1):2125-38. https://doi.org/10.1111/ejn.12243.
44. Fujita T, Yamasaki T, Kamio Y, Hirose S, Tobimatsu S.. Parvocellular pathway impairment in autism spectrum disorder: Evidence from visual evoked potentials. Res Autism Spectr Disord. 2011;5:277-285.
45. Fujita T, Kamio Y, Yamasaki T, Yasumoto S, Hirose S, Tobimatsu S. Altered automatic face processing in individuals with high-functioning autism spectrum disorders: evidence from visual evoked potentials. Res Autism Spectr Disord. 2013;7(6):710–20. https://doi.org/10.1016/j.rasd.2013.03.001.
46. Gaetz W, Jurkiewicz MT, Kessler SK, Blaskey L, Schwartz ES, Roberts TPL. Neuromagnetic responses to tactile stimulation of the fingers: Evidence for reduced cortical inhibition for children with Autism Spectrum Disorder and children with epilepsy. Neuroimage Clin. 2017;16:624-633. https://doi.org/10.1016/j.nicl.2017.06.026.
47. Gage NM, Siegel B, Roberts TP. Cortical auditory system maturational abnormalities in children with autism disorder: an MEG investigation. Brain Res Dev Brain Res. 2003;144(2):201-9. https://doi.org/10.1016/s0165-3806(03)00172-x.
48. Gomot M, Giard MH, Adrien JL, Barthelemy C, Bruneau N. Hypersensitivity to acoustic change in children with autism: electrophysiological evidence of left frontal cortex dysfunctioning. Psychophysiology. 2002;39(5):577-84. PMID: 12236323.
49. Gomot M, Blanc R, Clery H, Roux S, Barthelemy C, Bruneau N. Candidate electrophysiological endophenotypes of hyper-reactivity to change in autism. J Autism Dev Disord. 2011;41(6):705-14. https://doi.org/10.1007/s10803-010-1091-y.
50. Green HL, Shuffrey LC, Levinson L, et al. Evaluation of mismatch negativity as a marker for language impairment in autism spectrum disorder. J Commun Disord. 2020;87:105997. https://doi.org/10.1016/j.jcomdis.2020.105997.
51. Green HL, Shen G, Franzen RE, et al. Differential Maturation of Auditory Cortex Activity in Young Children with Autism and Typical Development. J Autism Dev Disord. 2023;53(10):4076-4089. https://doi.org/10.1007/s10803-022-05696-8.
52. Grisoni L, Moseley RL, Motlagh S, et al. Prediction and Mismatch Negativity Responses Reflect Impairments in Action Semantic Processing in Adults With Autism Spectrum Disorders. Front Hum Neurosci. 2019;13:395. https://doi.org/10.3389/fnhum.2019.00395.
53. Gunji A, Inagaki M, Inoue Y, Takeshima Y, Kaga M. Event-related potentials of self-face recognition in children with pervasive developmental disorders. Brain Dev. 2009;31(2):139-47. https://doi.org/10.1016/j.braindev.2008.04.011.
54. Gunji A, Goto T, Kita Y, Sakuma R, Kokubo N, Koike T, Sakihara K, Kaga M, Inagaki M. Facial identity recognition in children with autism spectrum disorders revealed by P300 analysis: a preliminary study. Brain Dev. 2013;35(4):293-8. https://doi.org/10.1016/j.braindev.2012.12.008.
55. Haigh SM, Brosseau P, Eack SM, Leitman DI, Salisbury DF, Behrmann M. Hyper-Sensitivity to Pitch and Poorer Prosody Processing in Adults With Autism: An ERP Study. Front Psychiatry. 2022;13:844830. https://doi.org/10.3389/fpsyt.2022.844830.
56. Hileman CM, Henderson H, Mundy P, Newell L, Jaime M. Developmental and individual differences on the P1 and N170 ERP components in children with and without autism. Dev Neuropsychol. 2011;36(2):214-36. https://doi.org/10.1080/87565641.2010.549870.
57. Høyland AL, Nærland T, Engstrøm M, Torske T, Lydersen S, Andreassen OA. Atypical event-related potentials revealed during the passive parts of a Go-NoGo task in autism spectrum disorder: a case-control study. Mol Autism. 2019;10:10. https://doi.org/10.1186/s13229-019-0259-3.
58. Huang D, Yu L, Wang X, Fan Y, Wang S, Zhang Y. Distinct patterns of discrimination and orienting for temporal processing of speech and nonspeech in Chinese children with autism: an event-related potential study. Eur J Neurosci. 2018;47(6):662-668. https://doi.org/10.1111/ejn.13657.
59. Hudac CM, DesChamps TD, Arnett AB, et al. Early enhanced processing and delayed habituation to deviance sounds in autism spectrum disorder. Brain Cogn. 2018;123:110-119. https://doi.org/10.1016/j.bandc.2018.03.004.
60. Isenstein EL, Grosman HE, Guillory SB, et al. Neural Markers of Auditory Response and Habituation in Phelan-McDermid Syndrome. Front Neurosci. 2022;16:815933. https://doi.org/10.3389/fnins.2022.815933.
61. Ji Y, Liu J, Zhu XQ, et al. Atypical N170 lateralization of face and word recognition in Chinese children with autism spectrum disorder. J Neurolinguistics. 2019;52:100858. https://doi.org/10.1016/j.jneuroling.2019.100858.
62. Jones EJH, Dawson G, Webb SJ. Sensory hypersensitivity predicts enhanced attention capture by faces in the early development of ASD. Dev Cogn Neurosci. 2018;29:11-20. https://doi.org/10.1016/j.dcn.2017.04.001.
63. Kadlaskar G, Bergmann S, McNally Keehn R, Seidl A, Keehn B. Electrophysiological Measures of Tactile and Auditory Processing in Children With Autism Spectrum Disorder. Front Hum Neurosci. 2021 Dec;15:729270. https://doi.org/10.3389/fnhum.2021.729270.
64. Kamita MK, Silva LAF, Magliaro FCL, Fernandes FD, Matas CG. Auditory Event Related Potentials in children with autism spectrum disorder. Int J Pediatr Otorhinolaryngol. 2021;148:110826. https://doi.org/10.1016/j.ijporl.2021.110826.
65. Kasai K, Hashimoto O, Kawakubo Y, et al. Delayed automatic detection of change in speech sounds in adults with autism: a magnetoencephalographic study. Clin Neurophysiol. 2005;116(7):1655-64. https://doi.org/10.1016/j.clinph.2005.03.007.
66. Kemner C, Oranje B, Verbaten MN, van Engeland H. Normal P50 gating in children with autism. J Clin Psychiatry. 2002;63(3):214-7. https://doi.org/10.4088/jcp.v63n0307.
67. Key AP, Corbett BA. ERP responses to face repetition during passive viewing: a nonverbal measure of social motivation in children with autism and typical development. Dev Neuropsychol. 2014;39(6):474-95. https://doi.org/10.1080/87565641.2014.940620.
68. Key AP, Thompson EC, Benítez-Barrera C, et al. Electrophysiological Measures of Listening-in-Noise With and Without Remote Microphone System Use in Autistic and Non-Autistic Youth. Ear Hear. 2024 May-Jun 01;45(3):710-720. https://doi.org/10.1097/AUD.0000000000001465.
69. Knight EJ, Oakes L, Hyman SL, Freedman EG, Foxe JJ. Individuals With Autism Have No Detectable Deficit in Neural Markers of Prediction Error When Presented With Auditory Rhythms of Varied Temporal Complexity. Autism Res. 2020;13(12):2058-2072. https://doi.org/10.1002/aur.2362.
70. Korpilahti P, Jansson-Verkasalo E, Mattila ML, et al. Processing of affective speech prosody is impaired in Asperger syndrome. J Autism Dev Disord. 2007;37(8):1539-49. https://doi.org/10.1007/s10803-006-0271-2.
71. Kovarski K, Thillay A, Houy-Durand E, et al. Brief Report: Early VEPs to Pattern-Reversal in Adolescents and Adults with Autism. J Autism Dev Disord. 2016 Oct;46(10):3377-86. https://doi.org/10.1007/s10803-016-2880-8.
72. Kovarski K, Malvy J, Khanna RK, Arsène S, Batty M, Latinus M. Reduced visual evoked potential amplitude in autism spectrum disorder, a variability effect? Transl Psychiatry. 2019;9,341.
73. Kröger A, Bletsch A, Krick C, et al. Visual event-related potentials to biological motion stimuli in autism spectrum disorders. Soc Cogn Affect Neurosci. 2014;9(8):1214-22. https://doi.org/10.1093/scan/nst103.
74. Kujala T, Lepistö T, Nieminen-von Wendt T, Näätänen P, Näätänen R. Neurophysiological evidence for cortical discrimination impairment of prosody in Asperger syndrome. Neurosci Lett. 2005;383(3):260-5. https://doi.org/10.1016/j.neulet.2005.04.048.
75. Kujala T, Aho E, Lepistö T, et al. Atypical pattern of discriminating sound features in adults with Asperger syndrome as reflected by the mismatch negativity. Biol Psychol. 2007;75(1):109-14. https://doi.org/10.1016/j.biopsycho.2006.12.007.
76. Kujala T, Kuuluvainen S, Saalasti S, Jansson-Verkasalo E, Wendt LV, Lepistö T. Speech-feature discrimination in children with Asperger syndrome as determined with the multi-feature mismatch negativity paradigm. Clin Neurophysiol. 2010;121(9):1410-1419. https://doi.org/10.1016/j.clinph.2010.03.017.
77. Lacroix, A., Harquel, S., Mermillod, M. et al. Sex modulation of faces prediction error in the autistic brain. Commun Biol. 2024;7,127.
78. Lambrechts A, Falter-Wagner CM, van Wassenhove V. Diminished neural resources allocation to time processing in Autism Spectrum Disorders. Neuroimage Clin. 2017;17:124-136. https://doi.org/10.1016/j.nicl.2017.09.023.
79. Lepistö T, Kujala T, Vanhala R, Alku P, Huotilainen M, Näätänen R. The discrimination of and orienting to speech and non-speech sounds in children with autism. Brain Res. 2005;1066(1-2):147-57. https://doi.org/10.1016/j.brainres.2005.10.052.
80. Lepistö T, Silokallio S, Nieminen-von Wendt T, Alku P, Näätänen R, Kujala T. Auditory perception and attention as reflected by the brain event-related potentials in children with Asperger syndrome. Clin Neurophysiol. 2006;117(10):2161-71. https://doi.org/10.1016/j.clinph.2006.06.709.
81. Lepistö T, Nieminen-von Wendt T, von Wendt L, Näätänen R, Kujala T. Auditory cortical change detection in adults with Asperger syndrome. Neurosci Lett. 2007;414(2):136-40. https://doi.org/10.1016/j.neulet.2006.12.009
82. Lepistö T, Kajander M, Vanhala R, et al. The perception of invariant speech features in children with autism. Biol Psychol. 2008;77(1):25-31. https://doi.org/10.1016/j.biopsycho.2007.08.010.
83. Lepistö T, Kuitunen A, Sussman E, et al. Auditory stream segregation in children with Asperger syndrome. Biol Psychol. 2009;82(3):301-7. https://doi.org/10.1016/j.biopsycho.2009.09.004. Erratum in: Biol Psychol. 2011;87(2):317.
84. Lincoln AJ, Courchesne E, Harms L, Allen M. Contextual probability evaluation in autistic, receptive developmental language disorder, and control children: event-related brain potential evidence. J Autism Dev Disord. 1993;23(1):37-58. https://doi.org/10.1007/BF01066417.
85. Lincoln AJ, Courchesne E, Harms L, Allen M. Sensory modulation of auditory stimuli in children with autism and receptive developmental language disorder: event-related brain potential evidence. J Autism Dev Disord. 1995;25(5):521-39. https://doi.org/10.1007/BF02178298.
86. Lindström R, Lepistö-Paisley T, Vanhala R, Alén R, Kujala T. Impaired neural discrimination of emotional speech prosody in children with autism spectrum disorder and language impairment. Neurosci Lett. 2016;628:47-51. https://doi.org/10.1016/j.neulet.2016.06.016.
87. Lindström R, Lepistö-Paisley T, Makkonen T, Reinvall O, Nieminen-von Wendt T, Alén R, Kujala T. Atypical perceptual and neural processing of emotional prosodic changes in children with autism spectrum disorders. Clin Neurophysiol. 2018;129(11):2411-2420. https://doi.org/10.1016/j.clinph.2018.08.018.
88. Luckhardt C, Kröger A, Cholemkery H, Bender S, Freitag CM. Neural Correlates of Explicit Versus Implicit Facial Emotion Processing in ASD. J Autism Dev Disord. 2017;47(7):1944-1955. https://doi.org/10.1007/s10803-017-3141-1.
89. Ludlow A, Mohr B, Whitmore A, Garagnani M, Pulvermüller F, Gutierrez R. Auditory processing and sensory behaviours in children with autism spectrum disorders as revealed by mismatch negativity. Brain Cogn. 2014;86:55-63. https://doi.org/10.1016/j.bandc.2014.01.016.
90. Lv M, Liu Y, Du Y, et al. Study on P50 Sensory Gating in Children with Autism Spectrum Disorders in Shanghai. N A J Med Sci. 2014;7(3). Retrieved from
91. Maekawa T, Tobimatsu S, Inada N, et al. Top-down and bottom-up visual information processing of non-social stimuli in high-functioning autism spectrum disorder. Res Autism Spectr Disord. 2011;5(1):201–9. https://doi.org/.
92. C M Magnée MJ, de Gelder B, van Engeland H, Chantal Kemner. Atypical processing of fearful face-voice pairs in Pervasive Developmental Disorder: an ERP study. Clin Neurophysiol. 2008;119(9):2004-10. https://doi.org/10.1016/j.clinph.2008.05.005.
93. Marco EJ, Khatibi K, Hill SS, et al. Children with autism show reduced somatosensory response: an MEG study. Autism Res. 2012;5(5):340-51. https://doi.org/10.1002/aur.1247.
94. Marsicano G, Casartelli L, Federici A, et al. Prolonged neural encoding of visual information in autism. Autism Res. 2024 Jan;17(1):37-54. https://doi.org/10.1002/aur.3062.
95. Mason L, Moessnang C, Chatham C, et al. Stratifying the autistic phenotype using electrophysiological indices of social perception. Sci Transl Med. 2022;14(658):eabf8987. https://doi.org/10.1126/scitranslmed.abf8987.
96. Matsuzaki J, Kagitani-Shimono K, Goto T, et al. Differential responses of primary auditory cortex in autistic spectrum disorder with auditory hypersensitivity. Neuroreport. 2012;23(2):113-8. https://doi.org/10.1097/WNR.0b013e32834ebf44. Erratum in: Neuroreport. 2012;23(11):698.
97. Matsuzaki J, Ku M, Dipiero M, et al. Delayed Auditory Evoked Responses in Autism Spectrum Disorder across the Life Span. Dev Neurosci. 2019;41(3-4):223-233. https://doi.org/10.1159/000504960.
98. Matsuzaki J, Kuschner ES, Blaskey L, et al. Abnormal auditory mismatch fields are associated with communication impairment in both verbal and minimally verbal/nonverbal children who have autism spectrum disorder. Autism Res. 2019;12(8):1225-1235. https://doi.org/10.1002/aur.2136.
99. Matsuzaki J, Ku M, Berman JI, et al. Abnormal auditory mismatch fields in adults with autism spectrum disorder. Neurosci Lett. 2019;698:140-145. https://doi.org/10.1016/j.neulet.2018.12.043.
100. McPartland J, Dawson G, Webb SJ, Panagiotides H, Carver LJ. Event-related brain potentials reveal anomalies in temporal processing of faces in autism spectrum disorder. J Child Psychol Psychiatry. 2004;45(7):1235-45. https://doi.org/10.1111/j.1469-7610.2004.00318.x.
101. McPartland JC, Wu J, Bailey CA, Mayes LC, Schultz RT, Klin A. Atypical neural specialization for social percepts in autism spectrum disorder. Soc Neurosci. 2011;6(5-6):436-51. https://doi.org/10.1080/17470919.2011.586880.
102. Megnin O, Flitton A, Jones CR, de Haan M, Baldeweg T, Charman T. Audiovisual speech integration in autism spectrum disorders: ERP evidence for atypicalities in lexical-semantic processing. Autism Res. 2012;5(1):39-48. https://doi.org/10.1002/aur.231.
103. Molholm S, Murphy JW, Bates J, Ridgway EM, Foxe JJ. Multisensory Audiovisual Processing in Children With a Sensory Processing Disorder (I): Behavioral and Electrophysiological Indices Under Speeded Response Conditions. Front Integr Neurosci. 2020;14:4. https://doi.org/10.3389/fnint.2020.00004.
104. Neuhaus E, Kresse A, Faja S, Bernier RA, Webb SJ. Face processing among twins with and without autism: social correlates and twin concordance. Soc Cogn Affect Neurosci. 2016;11(1):44-54. https://doi.org/10.1093/scan/nsv085.
105. O'Connor K, Hamm JP, Kirk IJ. The neurophysiological correlates of face processing in adults and children with Asperger's syndrome. Brain Cogn. 2005;59(1):82-95. https://doi.org/10.1016/j.bandc.2005.05.004.
106. O'Connor K, Hamm JP, Kirk IJ. Neurophysiological responses to face, facial regions and objects in adults with Asperger's syndrome: an ERP investigation. Int J Psychophysiol. 2007;63(3):283-93. https://doi.org/10.1016/j.ijpsycho.2006.12.001.
107. Oram Cardy JE, Ferrari P, Flagg EJ, Roberts W, Roberts TP. Prominence of M50 auditory evoked response over M100 in childhood and autism. Neuroreport. 2004;15(12):1867-70. https://doi.org/10.1097/00001756-200408260-00006.
108. Oram Cardy JE, Flagg EJ, Roberts W, Roberts TP. Auditory evoked fields predict language ability and impairment in children. Int J Psychophysiol. 2008;68(2):170-5. https://doi.org/10.1016/j.ijpsycho.2007.10.015.
109. Orekhova EV, Stroganova TA, Prokofyev AO, Nygren G, Gillberg C, Elam M. Sensory gating in young children with autism: relation to age, IQ, and EEG gamma oscillations. Neurosci Lett. 2008;434(2):218-23. https://doi.org/10.1016/j.neulet.2008.01.066.
110. Parker TC, Crowley MJ, Naples AJ, et al. The N170 event-related potential reflects delayed neural response to faces when visual attention is directed to the eyes in youths with ASD. Autism Res. 2021;14(7):1347-1356. https://doi.org/10.1002/aur.2505.
111. Peristeri E, Andreou M, Ketseridou SN, et al. Animacy Processing in Autism: Event-Related Potentials Reflect Social Functioning Skills. Brain Sci. 2023;13(12):1656. https://doi.org/10.3390/brainsci13121656.
112. Piatti A, Van der Paelt S, Warreyn P, Roeyers H. Atypical attention to voice in toddlers and pre-schoolers with autism spectrum disorder is related to unimpaired cognitive abilities: an ERP study. Res Autism Spectr Disord. 2021;86:101805. https://doi.org/10.1016/j.rasd.2021.101805.
113. Portnova GV, Skorokhodov IV, Mayorova LA. The Levels of Auditory Processing during Emotional Perception in Children with Autism. J Integr Neurosci. 2023;22(5):112. https://doi.org/10.31083/j.jin2205112.
114. Richards JE, Guy MW, Hogan AL, Roberts JE. Neural correlates of face processing among preschoolers with fragile X syndrome, autism spectrum disorder, autism siblings, and typical development. Autism Res. 2024;17(1):89-108. https://doi.org/10.1002/aur.3045.
115. Roberts TP, Khan SY, Rey M, et al. MEG detection of delayed auditory evoked responses in autism spectrum disorders: towards an imaging biomarker for autism. Autism Res. 2010;3(1):8-18. https://doi.org/10.1002/aur.111.
116. Roberts TP, Cannon KM, Tavabi K, et al. Auditory magnetic mismatch field latency: a biomarker for language impairment in autism. Biol Psychiatry. 2011;70(3):263-9. https://doi.org/10.1016/j.biopsych.2011.01.015.
117. Roberts TPL, Matsuzaki J, Blaskey L, et al. Delayed M50/M100 evoked response component latency in minimally verbal/nonverbal children who have autism spectrum disorder. Mol Autism. 2019;10:34. https://doi.org/10.1186/s13229-019-0283-3.
118. Randeniya R, Mattingley JB, Garrido MI. Increased context adjustment is associated with auditory sensitivities but not with autistic traits. Autism Res. 2022;15(8):1457-1468. https://doi.org/10.1002/aur.2759.
119. Ruiz-Martínez FJ, Rodríguez-Martínez EI, Wilson CE, Yau S, Saldaña D, Gómez CM. Impaired P1 Habituation and Mismatch Negativity in Children with Autism Spectrum Disorder. J Autism Dev Disord. 2020;50(2):603-616. https://doi.org/10.1007/s10803-019-04299-0.
120. Sakihara K, Kita Y, Suzuki K, Inagaki M. Modulation effects of the intact motor skills on the relationship between social skills and motion perceptions in children with autism spectrum disorder: A pilot study. Brain Dev. 2023;45(1):39-48. https://doi.org/10.1016/j.braindev.2022.09.001.
121. Schwartz S, Wang L, Uribe S, Shinn-Cunningham BG, Tager-Flusberg H. Auditory evoked potentials in adolescents with autism: An investigation of brain development, intellectual impairment, and neural encoding. Autism Res. 2023;16(10):1859-1876. https://doi.org/10.1002/aur.3003.
122. Senju A, Tojo Y, Yaguchi K, Hasegawa T. Deviant gaze processing in children with autism: an ERP study. Neuropsychologia. 2005;43(9):1297-306. https://doi.org/10.1016/j.neuropsychologia.2004.12.002.
123. Shen IH, Lin SC, Wu YY, Chen CL. An Event-Related Potential Study on the Perception and the Recognition of Face, Facial Features, and Objects in Children With Autism Spectrum Disorders. Percept Mot Skills. 2017;124(1):145-165. https://doi.org/10.1177/0031512516681694.
124. Shuffrey LC, Levinson L, Becerra A, et al. Visually Evoked Response Differences to Contrast and Motion in Children with Autism Spectrum Disorder. Brain Sci. 2018;8(9):160. https://doi.org/10.3390/brainsci8090160.
125. Sokhadze E, Baruth J, Tasman A, et al. Event-related potential study of novelty processing abnormalities in autism. Appl Psychophysiol Biofeedback. 2009;34(1):37-51. https://doi.org/10.1007/s10484-009-9074-5.
126. Sokhadze EM, Baruth JM, Sears L, et al. Event-related potential study of attention regulation during illusory figure categorization task in ADHS, autism spectrum disorder and typical children. J Neurother. 2012;16(1):12-31. https://doi.org/10.1080/10874208.2012.650119.
127. Sokhadze EM, Tasman A, Sokhadze GE, El-Baz AS, Casanova MF. Behavioral, Cognitive, and Motor Preparation Deficits in a Visual Cued Spatial Attention Task in Autism Spectrum Disorder. Appl Psychophysiol Biofeedback. 2016;41(1):81-92. https://doi.org/10.1007/s10484-015-9313-x.
128. Sokhadze EM, Casanova MF, Tasman A, Brockett S. Electrophysiological and Behavioral Outcomes of Berard Auditory Integration Training (AIT) in Children with Autism Spectrum Disorder. Appl Psychophysiol Biofeedback. 2016;41(4):405-420. https://doi.org/10.1007/s10484-016-9343-z.
129. Tanaka M, Yamada E, Yamasaki T, et al. Asynchronous neural oscillations associated with subliminal affective face priming in autism spectrum disorder. Neuroreport. 2023;34(3):150-155. https://doi.org/10.1097/WNR.0000000000001871.
130. Tavares PP, Mouga SS, Oliveira GG, Castelo-Branco M. Preserved face inversion effects in adults with autism spectrum disorder: an event-related potential study. Neuroreport. 2016;27(8):587-92. https://doi.org/10.1097/WNR.0000000000000576.
131. Toffoli L, Scerif G, Snowling MJ, Norcia AM, Manning C. Global motion evoked potentials in autistic and dyslexic children: A cross-syndrome approach. Cortex. 2021;143:109-126. https://doi.org/10.1016/j.cortex.2021.06.018.
132. van Laarhoven T, Stekelenburg JJ, Eussen MLJM, Vroomen J. Electrophysiological alterations in motor-auditory predictive coding in autism spectrum disorder. Autism Res. 2019;12(4):589-599. https://doi.org/10.1002/aur.2087.
133. Vlaskamp C, Oranje B, Madsen GF, et al. Auditory processing in autism spectrum disorder: Mismatch negativity deficits. Autism Res. 2017;10(11):1857-1865. https://doi.org/10.1002/aur.1821.
134. Wagner JB, Hirsch SB, Vogel-Farley VK, Redcay E, Nelson CA. Eye-tracking, autonomic, and electrophysiological correlates of emotional face processing in adolescents with autism spectrum disorder. J Autism Dev Disord. 2013;43(1):188-99. https://doi.org/10.1007/s10803-012-1565-1.
135. Wang X, Wang S, Fan Y, Huang D, Zhang Y. Speech-specific categorical perception deficit in autism: An Event-Related Potential study of lexical tone processing in Mandarin-speaking children. Sci Rep. 2017;7:43254. https://doi.org/10.1038/srep43254.
136. Webb SJ, Dawson G, Bernier R, Panagiotides H. ERP evidence of atypical face processing in young children with autism. J Autism Dev Disord. 2006;36(7):881-90. https://doi.org/10.1007/s10803-006-0126-x.
137. Weismüller B, Thienel R, Youlden AM, Fulham R, Koch M, Schall U. Psychophysiological Correlates of Developmental Changes in Healthy and Autistic Boys. J Autism Dev Disord. 2015;45(7):2168-75. https://doi.org/10.1007/s10803-015-2385-x.
138. Whitehouse AJ, Bishop DV. Do children with autism 'switch off' to speech sounds? An investigation using event-related potentials. Dev Sci. 2008;11(4):516-24. https://doi.org/10.1111/j.1467-7687.2008.00697.x.
139. Yamasaki T, Fujita T, Ogata K, et al. Electrophysiological evidence for selective impairment of optic flow perception in autism spectrum disorder. Res Autism Spectr Disord. 2011;5:400-7.
140. Yamasaki T, Maekawa T, Miyanaga Y, et al. Enhanced Fine-Form Perception Does Not Contribute to Gestalt Face Perception in Autism Spectrum Disorder. PLoS One. 2017;12(2):e0170239. https://doi.org/10.1371/journal.pone.0170239.
141. Yoshimura Y, Kikuchi M, Hiraishi H, et al. Atypical development of the central auditory system in young children with Autism spectrum disorder. Autism Res. 2016;9(11):1216-1226. https://doi.org/10.1002/aur.1604.
142. Yoshimura Y, Ikeda T, Hasegawa C, et al. Shorter P1m Response in Children with Autism Spectrum Disorder without Intellectual Disabilities. Int J Mol Sci. 2021;22(5):2611. https://doi.org/10.3390/ijms22052611.
143. Yu L, Fan Y, Deng Z, Huang D, Wang S, Zhang Y. Pitch Processing in Tonal-Language-Speaking Children with Autism: An Event-Related Potential Study. J Autism Dev Disord. 2015;45(11):3656-67. https://doi.org/10.1007/s10803-015-2510-x.
144. Yu L, Wang S, Huang D, Wu X, Zhang Y. Role of inter-trial phase coherence in atypical auditory evoked potentials to speech and nonspeech stimuli in children with autism. Clin Neurophysiol. 2018;129(7):1374-1382. https://doi.org/10.1016/j.clinph.2018.04.599.
145. Yu L, Huang D, Wang S, Wu X, Chen Y, Zhang Y. Evidence of Altered Cortical Processing of Dynamic Lexical Tone Pitch Contour in Chinese Children with Autism. Neurosci Bull. 2021;37(11):1605-1608. https://doi.org/10.1007/s12264-021-00752-2.

1. A study quality rating was implemented based on Newcastle-Ottawa quality assessment scale for case-control studies plus self-constructed rating scale for EEG/MEG signal quality. [↑](#footnote-ref-1)
